# Decoded fingerprints of hyperresponsive, expanding product space: polyether cascade cyclizations as tools to elucidate supramolecular catalysis†

**DOI:** 10.1039/d2sc03991e

**Published:** 2022-08-31

**Authors:** Hao Chen, Tian-Ren Li, Naomi Sakai, Celine Besnard, Laure Guénée, Marion Pupier, Jasmine Viger-Gravel, Konrad Tiefenbacher, Stefan Matile

**Affiliations:** National Centre of Competence in Research (NCCR) Molecular Systems Engineering BPR 1095 Basel Switzerland; School of Chemistry and Biochemistry University of Geneva Geneva Switzerland stefan.matile@unige.ch https://www.unige.ch/sciences/chiorg/matile/ +41 22 379 6523; Department of Chemistry, University of Basel Basel Switzerland; Department of Biosystems Science and Engineering, ETH Zurich Basel Switzerland

## Abstract

Simple enough to be understood and complex enough to be revealing, cascade cyclizations of diepoxides are introduced as new tools to characterize supramolecular catalysis. Decoded product fingerprints are provided for a consistent set of substrate stereoisomers, and shown to report on chemo-, diastereo- and enantioselectivity, mechanism and even autocatalysis. Application of the new tool to representative supramolecular systems reveals, for instance, that pnictogen-bonding catalysis is not only best in breaking the Baldwin rules but also converts substrate diastereomers into completely different products. Within supramolecular capsules, new cyclic hemiacetals from House–Meinwald rearrangements are identified, and autocatalysis on anion–π catalysts is found to be independent of substrate stereochemistry. Decoded product fingerprints further support that the involved epoxide-opening polyether cascade cyclizations are directional, racemization-free, and interconnected, at least partially. The discovery of unique characteristics for all catalysts tested would not have been possible without decoded cascade cyclization fingerprints, thus validating the existence and significance of privileged platforms to elucidate supramolecular catalysis. Once decoded, cascade cyclization fingerprints are easily and broadly applicable, ready for use in the community.

## Abstract 2

Hyperresponsive XL product space identifies polyether cascade fingerprinting as an attractive tool to elucidate supramolecular catalysis, including pnictogen-bonding, capsule and anion–π catalysts.

## Graphical abstract

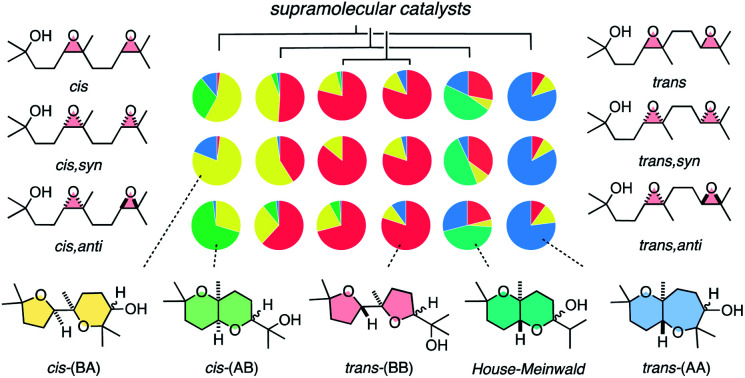

One general expectation from supramolecular catalysis^1–10^ is that new ways to interact will provide new ways to transform on the molecular level. This translates to access to new reactivity and products, at best contributing to new solutions for otherwise persistent challenges in science and society. While these high expectations are attracting attention to the development of supramolecular catalysts, their systematic characterization is much less advanced. Most classical and modern benchmark reactions^1,9^ are limited to one mechanism and cover little product space, also concerning chemo- and stereochemistry. To maximize the comparability of supramolecular catalysts, the ideal reaction would respond to as many parameters as possible at still manageable complexity. Epoxide opening polyether cascade cyclizations^10–13^ promise to meet these requirements for a privileged platform to evaluate supramolecular catalysts. Charismatic in chemistry and biology, they have attracted the attention of many giants in the field.^11^ They afford the largest polycyclic natural products, regularly featuring more than 10 rings made in one cascade. While product diversity of longer cascades is too complex and single cyclizations are too simple, minimalist cascades from diepoxide substrates such as 1 cover large structural space at tractable complexity (Fig. 1). In substrate 1, supramolecular catalysts can activate nucleophile, electrophile and leaving group, and stabilize cationic and anionic transition states and reactive intermediates (Fig. 1a). Cyclizations can follow either the 5-*exo-tet* selectivity predicted by the Baldwin rules (B) or anti-Baldwin (A) 6-*endo-tet* selectivity, leading to the four constitutional isomers 2–5 (Fig. 1a and 2). They can occur with normal or reverse directionally,^12^ forming ring 1 or ring 2 first, respectively (Fig. 1b). They can operate with pseudo S_N_2, S_N_1, or mixed mechanisms, and can integrate contributions from autocatalysis.^10,13^ The stereochemistry covers *cis*–*trans* isomers at epoxide 1 and *syn*–*anti* isomers with regard to the two epoxides (Fig. 1c). This translates to the stereochemistry of products such as 2–5 at the ring junction and the exocyclic substituents of ring 2. Besides this expected diversity, the product space of the privileged substrate 1 further expands into structures that remain to be discovered, as demonstrated with two new products reported in this study.

**Fig. 1:**
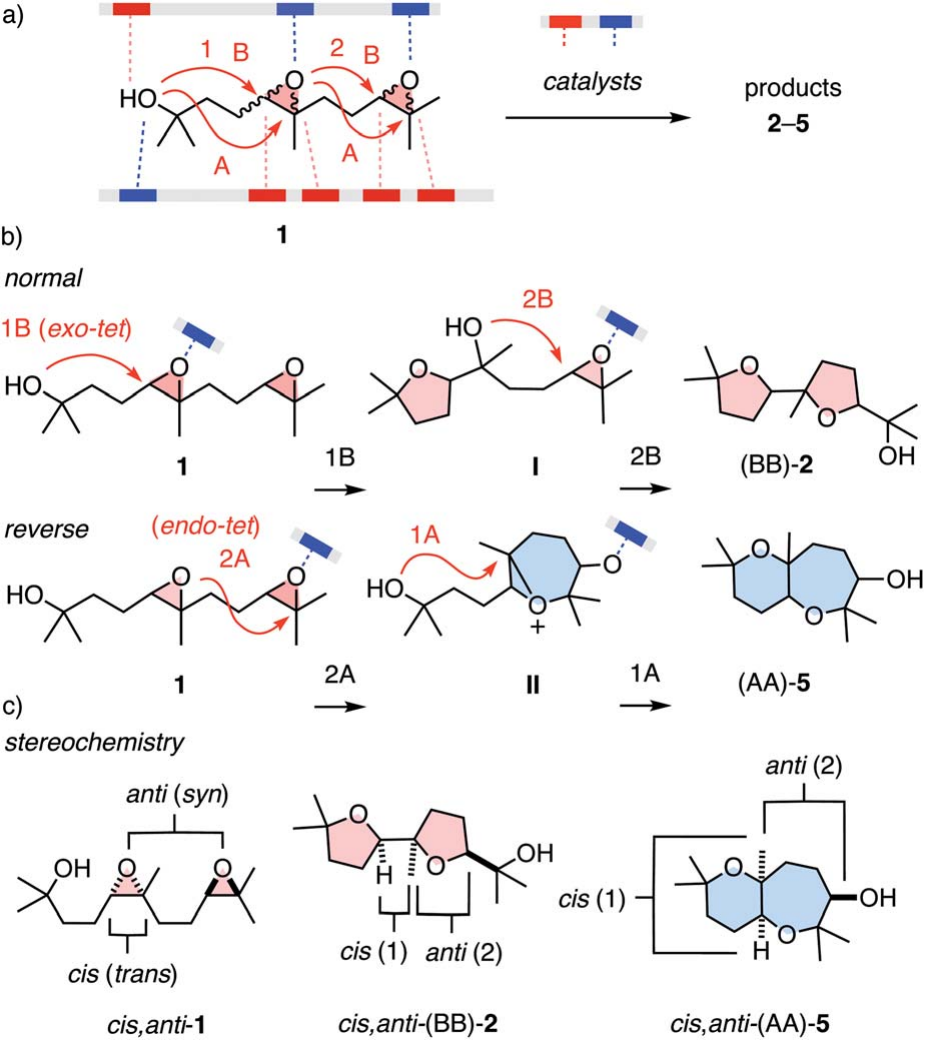
(a) Epoxide-opening ether cascade cyclizations from diepoxide 1 as privileged platform to elaborate on supramolecular catalysis, with indication of possible contributions from electron-donating (red) and electron-accepting catalyst motifs (blue), *exo-tet* Baldwin (B) or *endo-tet* anti-Baldwin (A) chemoselectivity, (b) normal and reverse directionality, and (c) stereochemistry in selected substrates and products.

**Fig. 2:**
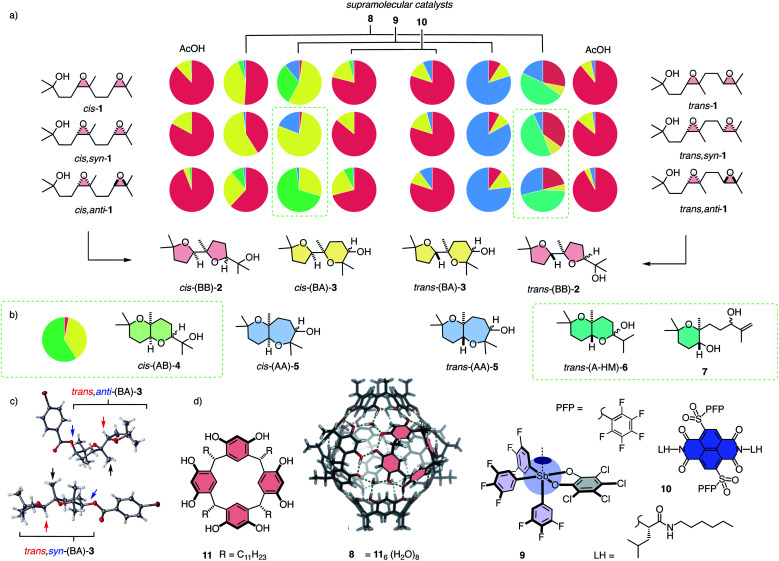
(a) Decoded product fingerprints for selected catalysts: Color-coded pie charts for products 2 (red), 3 (yellow), 4 (green), 5 (blue), 6 and 7 (teal) obtained from stereoisomers of *cis* and *trans* substrate isomers 1 with representative supramolecular catalysts 8–10 compared to general Brønsted acid (AcOH); results for *cis*,*anti* and *trans*,*syn* isomers of 1 are calculated (from data for the other diastereomer and the mixture of diastereomers in the respective series); estimated errors ± 5%. (b) Experimental results for *cis*,*anti*-1 cyclized with catalyst 9. (c) Selected X-ray structures from the BA series (*p*-bromobenzoyl derivatives). (d) Structure of catalysts, with indication of selected π-basic surfaces and hydrogen-bond donors on capsule 8 assembled from monomers 11, the cyclopean σ hole of pnictogen-bonding catalyst 9, and the π-acidic surface on anion–π catalyst 10.

So far, substrate 1 has been used as a mixture of stereoisomers to characterize supramolecular catalysts.^13^ While results were intriguing, they could not be rationalized. Overlap of different trends obscured the key information and made product fingerprints dependent on the composition of the substrate mixtures. However, the observed hyperresponsiveness of the large product space suggested that decoded product fingerprints could provide a general tool to elucidate supramolecular catalysis.

To assess the possibly privileged nature of diepoxide 1 as unifying substrate for supramolecular catalysis, we decided to synthesize and evaluate the necessary stereoisomers separately. The stereoisomers *cis*-1 and *trans*-1 were prepared by oxidation of the respective silyl protected *cis*- and *trans*-olefins with *m*-CPBA (meta-chloroperoxybenzoic acid), followed by deprotection (Fig. 2, Schemes S1 and S2†). They were obtained as roughly equimolar mixtures of *syn*- and *anti*-diastereomers (*cis*-1: dr 54 : 46, *trans*-1: dr 50 : 50). Shi epoxidation^14^ in place of *m*-CPBA afforded enantioenriched *cis*,*syn*-1 (dr 89 : 11; dr 20 : 1 after purification) and *trans*,*anti*-1 (dr 82 : 18; dr 20 : 1 after purification) accordingly with unknown absolute configuration. These four substrates were sufficient to realize the complete analysis of the system because the product fingerprints for the remaining diastereomers *cis*,*anti*-1 and *trans*,*syn*-1 could be obtained from the difference of *cis*,*syn*-1 and *trans*,*anti*-1 and the respective mixture of diastereomers *cis*-1 and *trans*-1 (Fig. 2).

To decode product fingerprints from different catalysts in their respective color-coded pie charts, all individual products were isolated and the diagnostic regions of their ^1^H NMR spectra and chiral GC traces were assembled for direct comparison (Fig. 3). In most GC traces, the two peaks were well resolved for each pair of enantiomers, confirming access to nearly all stereochemical information. The resulting unified fingerprint of the complete system then allowed to rapidly assign products obtained from different catalysts down to the level of enantiomers. The validity of most structures was confirmed by X-ray crystallography (Fig. 2c and S78–S83†). If necessary, derivatives were prepared to facilitate the growth of single crystals.

**Fig. 3:**
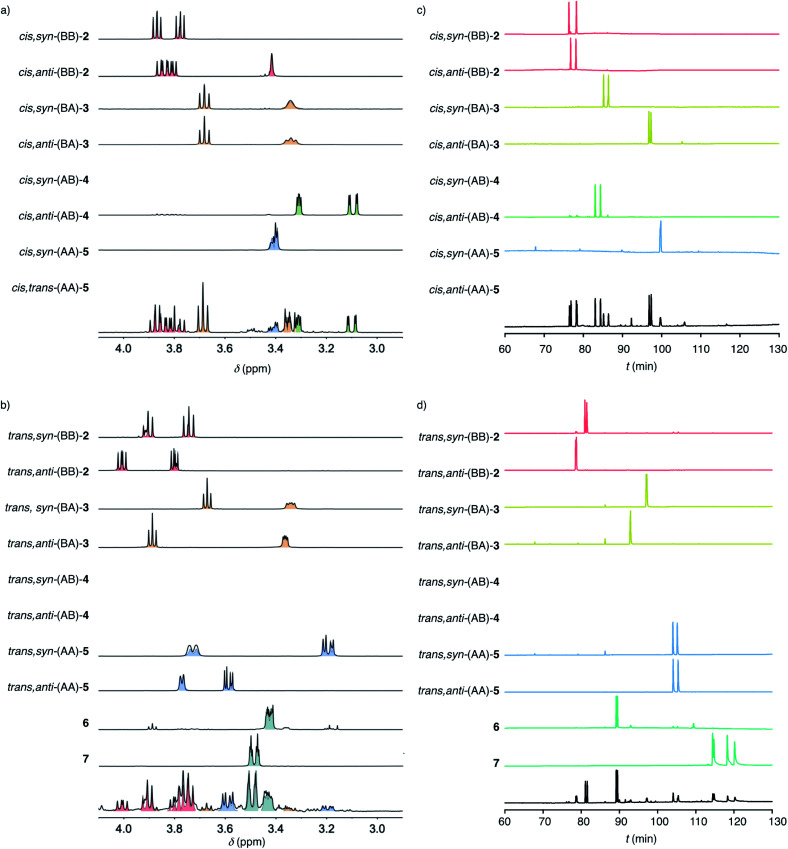
Decoded product fingerprints: Diagnostic regions of ^1^H NMR spectra (a and b) and chiral GC (c and d) of purified cascade cyclization products from *cis* (a and c) and *trans* (b and d) substrate isomers 1 above representative examples of mixtures produced by AcOH and 9 combined (a and c), and by 8 (b and d).

With the analytics in place, product fingerprints were recorded for representative supramolecular catalysts 8–10 in comparison to general Brønsted acid catalysis (Fig. 2). In the *cis* series, the product mixtures obtained from different catalysts contained all four constitutional isomers expected from Baldwin and anti-Baldwin cyclizations, that is *cis*-(BB)-2, *cis*-(BA)-3, *cis*-(AB)-4 and *cis*-(AA)-5 (Fig. 2a and 3a). In contrast, *trans*-(AB)-4 was absent in the *trans* series, and two new products 6 and 7 were found instead (*vide infra*, Fig. 2a and 3b).

In both the *cis* and the *trans* series, general Brønsted acid catalysis with AcOH was confirmed to follow the Baldwin rules almost exclusively, affording mostly (BB)-2 (Fig. 2). In the *cis* series, the supramolecular capsules 8 violated the Baldwin rules significantly (Fig. 2a). Capsules 8 self-assemble from resorcinarenes 11 and water (Fig. 2d).^3,4^ Their internal surface offers hydrogen-bond donors and π-basic aromatic planes for catalysis within their confined interior.^3,4^ Unique selectivities have been reported, also for bioinspired terpene cyclizations, for instance.^4^ From cascade cyclization with the mixture of *cis*-1 diastereomers in capsules 8, *cis*-(BA)-3 was obtained as the main product besides the still preferred *cis*-(BB)-2 (Fig. 2a). The pure *cis*,*syn*-1 showed a clearly different product distribution, characterized by an increased power to violate the Baldwin rule in cycle 2, affording *cis*-(BA)-3 as the main product. The calculated fingerprint for the products of *cis*,*anti*-1 gave the complementary dominance of the Baldwin conformant *cis*-(BB)-2 instead.

Differences in selectivity for the *syn*- and *anti*-diastereomers in the *cis* series were most spectacular with the pnictogen-bonding catalyst 9 (Fig. 2a). Pnictogen-bonding catalysis has been introduced recently^5–7^ for consideration as the non-covalent counterpart of Lewis acid catalysis, analogous to hydrogen-bonding catalysis as non-covalent counterpart of Brønsted acid catalysis.^7^ Catalyst 9 is centered around an antimony V with one deep σ hole acting as pnictogen-bond donor to initiate catalysis.^7^ Catalyst 9 has been shown previously to efficiently break the Baldwin rules in polyether cyclizations.^7,13^ In the newly devised pie chart fingerprint, orthodox *cis*-(BB)-2 was indeed essentially absent (Fig. 2a). The mixture of diastereomers *cis*-1 afforded *cis*-(BA)-3 and *cis*-(AB)-4 as main products. In sharp contrast, diastereo-pure *cis*,*syn*-1 gave mostly *cis*,*syn*-(BA)-3. As a consequence, the calculated product fingerprint of *cis*,*anti*-1 showed the highly selective formation of *cis*,*anti*-(AB)-4.

Selective access to *cis*,*anti*-(AB)-4 with pnictogen-bonding catalyst 9 was remarkable because none of the other stereoisomers of (AB)-4 were observed throughout the study (Fig. 3a and 4a). Exclusive access to *cis*,*anti*-(AB)-4 from *cis*,*anti*-1 was understandable considering cascade cyclization with normal directionality (Fig. 1). Namely, the *endo-tet* cyclization of ring 1 will afford the reactive intermediate III (Fig. 4b). From this intermediate III, the *exo-tet* Baldwin conformant formation of ring 2 is possibly supported by an intramolecular hydrogen bond (Fig. 4b and c, arrows), which activates the nucleophile and places an epoxide in an equatorial position.

**Fig. 4:**
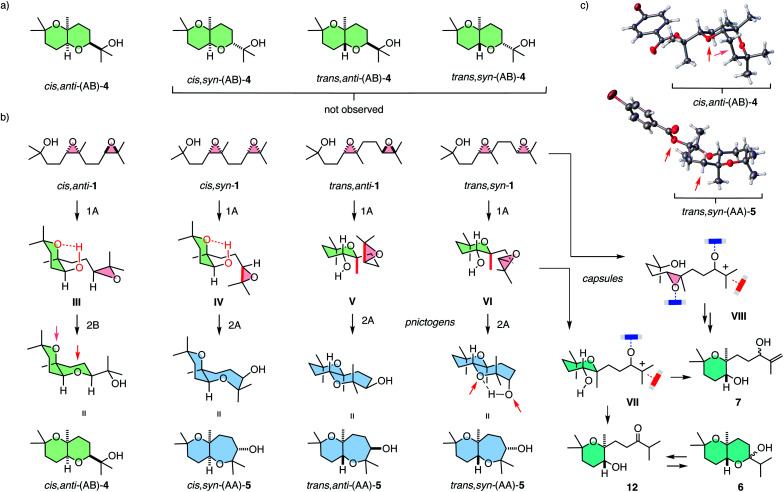
(a) The formation of only one out of four possible (AB) isomers 4 and (b) the origin of the selectivity and products found in the anti-Baldwin series with capsule 8 and pnictogen-bonding catalyst 9, with (c) selected X-ray structures.

These favorable conditions to access *cis*,*anti*-(AB)-4 from *cis*,*anti*-1 contrasted sharply with the situation with all other diastereomers. In the *cis* series, access to the complementary *cis*,*syn*-(AB)-4 from *cis*,*syn*-1 is disfavored although the nucleophile in the reactive intermediate IV remains possibly activated by intramolecular hydrogen bonding. However, the axial orientations of epoxide in intermediate IV and a very bulky tertiary alcohol in product 4 make this reaction unlikely.

With *cis*,*syn*-(AB)-4 from *cis*,*syn*-1 unfavorable, reactive intermediate IV obtained from an anti-Baldwin cyclization of ring 1 needs an alternative solution. An obvious choice is continuation with another *endo-tet* anti-Baldwin cyclization for ring 2 to result in *cis*,*syn*-(AA)-5 with a more flexible *cis*-fused oxepane ring. This *cis*,*syn*-(AA)-5 was indeed part of the product fingerprint of *cis*,*syn*-1 cyclized with pnictogen-bonding catalyst 9 (Fig. 2a). The markedly different amounts of *cis*,*syn*-(AA)-5 and *cis*,*anti*-(AB)-4 obtained from *cis*,*syn*-1 and *cis*,*anti*-1, respectively (Fig. 2a), would then suggest that normal cascade cyclizations are interconnected, possibly concerted (Fig. 1b).

The differences of the selectivity of the cascade cyclization of *cis*,*syn*-1 and *cis*,*anti*-1 with pnictogen-bonding catalyst 9 (Fig. 2a) and the importance of the implications called for the experimental validation of the calculated results for *cis*,*anti*-1. Therefore, pure diastereomer *cis*,*anti*-1 was prepared and cyclized using catalyst 9. The experimental product fingerprint was very similar to the calculated one, confirming the unique *cis*,*anti*-(AB)-4 as the main product of the reaction (Fig. 2b). This results also validated the use of calculated data to decode complex product fingerprints completely.

In the *trans* series, pnictogen-bonding catalyst 9 again broke the Baldwin rules most efficiently (Fig. 2a). For all diastereomers, *trans*-(AA)-5 was observed as the main product with more than 75% yield. This exceptional selectivity was understandable considering the reactive intermediates V and VI after the *endo-tet* cyclization of ring 1 (Fig. 4b). Contrary to intermediates III and IV in the *cis* series, the methyl substituent at the ring junction is in axial position also with regard to ring 2. 1,3-Diaxial interactions of the approaching electrophile with this methyl thus hinder the formation of this ring 2 by an *exo-tet* cyclization. Presumably for this reason, the *trans*-fused bis-oxane products *trans*,*anti*-(AB)-4 and *trans*,*syn*-(AB)-4 were not observed. With Baldwin cyclizations hindered, *endo-tet* anti-Baldwin cyclizations occurred instead to afford the respective *trans*,*anti*-(AA)-5 and *trans*,*syn*-(AA)-5 with very high selectivity (Fig. 2a).

The supramolecular capsules 8 applied to the *trans* series yielded two new products 6 and 7 (Fig. 2). Product 6 was identified by 2D NMR spectroscopy to be a hemiacetal cyclized on an anti-Baldwin ring 1 (Fig. S72†). It exists in equilibrium with the open ketone form 12, which results in dynamic epimerization at the “anomeric center” (Fig. 2 and 4). Derivatization of hemiacetal 6 with aromatic hydrazines gave the respective hydrazones (Fig. S76 and S77†). Product 7 was identified as an acyclic allyl alcohol extending from an anti-Baldwin ring 1 (Fig. 2 and S73–S75†). Both new products might originate from intermediate VII, which is generated from substrate 1 by *endo-tet* cyclization of ring 1 and the opening of epoxide 2 to afford the tertiary carbocation (Fig. 4b). From intermediate VII, the formation of allyl alcohol 7 only requires a proton abstraction from one of the two adjacent methyl groups. Ketone 12 originates from the same intermediate VII*via* House-Meinwald rearrangement,^15^ that is a 1,2-hydride shift. Similar processes might occur with *trans*-diepoxide 1 to give an alternative cationic intermediate VIII, which can proceed through reverse cyclization (Fig. 1b) to give products 6 and 7. Stabilization of carbocations *via* cation–π interactions is a distinct feature of this type of capsules.^3,4^

The formation of these two new products in capsule 8 could be understood considering the inaccessibility of both AB products in the *trans* series, *i.e.*, *trans*,*syn*-(AB)-4 and *trans*,*anti*-(AB)-4, with the explored catalysts (Fig. 4a). As already mentioned, the anti-Baldwin cyclization from *trans*,*anti*-1 and *trans*,*syn*-1 into intermediates V and VI with ring 1 is unproblematic, whereas continuation with *exo-tet* Baldwin cyclization of ring 2 is hindered by an axial methyl and, compared to the *cis* series, missing intramolecular activation of the nucleophile (Fig. 4b). With pnictogen-bonding catalyst 9, the solution was an alternative *endo-tet* anti-Baldwin cyclization into the *trans*-fused AA products 5, as discussed above (Fig. 2a and 4b). In capsule 8, this *endo-tet* anti-Baldwin continuation of the cascade was not favorable. The reason for this distinctive selectivity within capsule 8 remains to be explored. In contrast to the other catalysts, the capsule may be able to stabilize cation VII better due to cation–π stabilization, making this pathway accessible.

While the new oxanes 6 and 7 were obtained as main products from *trans*,*anti*-1 and *trans*,*syn*-1 with similar yields, the composition of the side products differed in the respective fingerprints (Fig. 2a). Cyclization of *trans*,*syn*-1 gave *trans*-(BB)-2 as the main side product, while *trans*,*anti*-1 gave *trans*,*anti*-(AA)-5 as the main side product. This difference was of interest because it could support that the cascade cyclizations might be interconnected, possibly concerted, at least in the present context.

While capsules 8 excelled with access to new products in the *trans* series and pnictogen-bonding catalysts 9 with unique AB-BA selectivity on the level of diastereomers in the *cis* series, anion–π catalysts gave mostly Baldwin products like general Brønsted acid catalysis, independent of the stereochemistry of substrate 1 (Fig. 2). The largest deviation from Brønsted acid catalysis occurred with *cis*,*anti*-1, which gave a substantial percentage of *cis*-(BA)-3 and also a small amount of *cis*-(AB)-4 (Fig. 2a). The same trend, but less pronounced, was noted with the complementary *trans*,*anti*-1, which produced also small amounts of *trans*-(BA)-3 and *trans*-(AA)-5, formed instead of the inaccessible *trans*-(AB)-4 (see above, Fig. 2a).

After investigation for anion transport, anion–π interactions have been introduced to catalysis in stabilizing anionic transition states on π-acidic surfaces.^8,10^ Over the past decade, catalysts from hexafluorobenzene to π-stacked foldamers, fullerenes, carbon nanotubes, artificial enzymes have been applied to many reactions, including enolate, enamine, imine, Diels–Alder chemistry.^8^ Polyether cyclizations have been introduced as a cascade transformation that should benefit best from the delocalized nature of anion–π interactions.^10^ On π-acidic surfaces, polyether cyclizations were autocatalytic,^10^ a unique emergent property that has not been observed in the many studies with systems without anion–π interactions.^11^

With the privileged probe for supramolecular catalysis envisioned in this study, it was thus most interesting to assess the dependence of autocatalysis on the stereochemistry of the substrate. Significant dependence was conceivable considering the different products obtained from diastereomers of *cis*-1 with pnictogen-bonding catalyst 9 (Fig. 2a). Kinetics of all four test substrates converted with anion–π catalyst showed autocatalytic behavior (Fig. 5a and b). Moreover, autocatalysis was nearly independent of the stereochemistry of the substrate. This absence of diastereoselective autocatalysis was consistent with the computed model for transition-state stabilization by the product, and could explain why it is so difficult to achieve asymmetric autocatalysis on anion–π catalyst 10.^13^ Control experiments confirmed that general Brønsted acid catalysis does not show autocatalytic behavior, independent of the stereochemistry of substrate 1 (Fig. 5c and d).

**Fig. 5:**
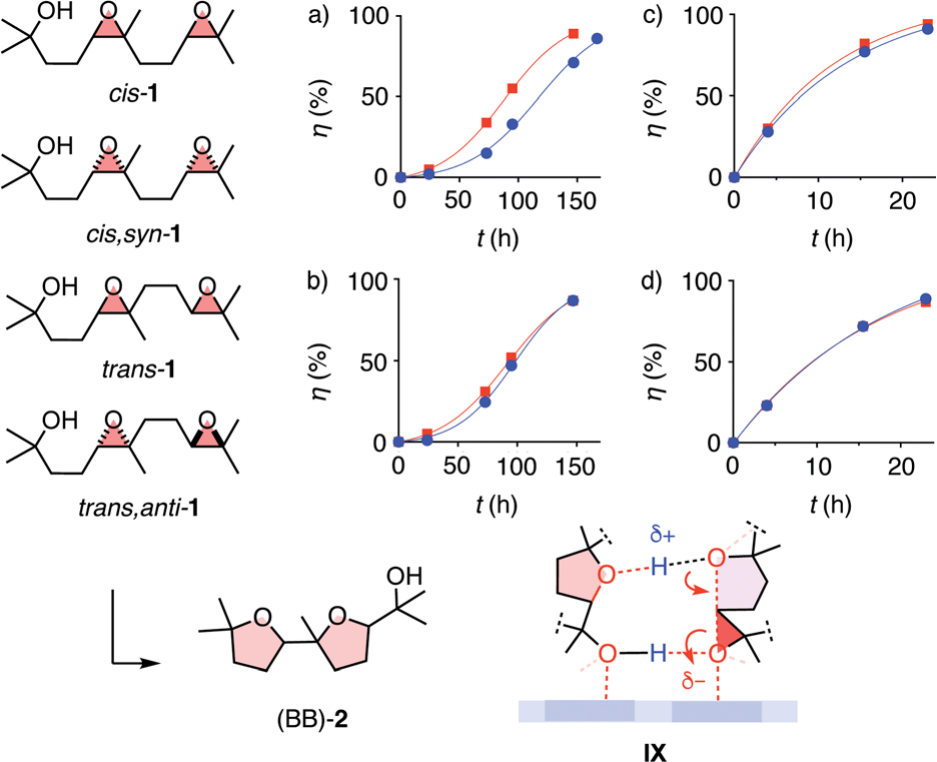
Kinetics of the conversion of *cis*-1 (a and c, circles), *cis*,*syn*-1 (a and c, squares), *trans*-1 (b and d, circles) and *trans*,*anti*-1 (b and d, squares) with (a and b) anion–π catalyst 10 (10 mol%, rt) and (c and d) AcOH (500 mol%, 40 °C) in CD_2_Cl_2_, with hypothetical intermediate IX for autocatalysis on π-acidic surfaces.

Taken together, the decoding of product fingerprints for cascade cyclizations that are simple enough to be tractable and complex enough to be interesting affords a privileged platform to characterize supramolecular catalysis. It is highly responsive to as many characteristics as possible, thus reporting on as many distinct advantages of the catalytic system as possible. The minimal substrate toolbox contains *cis* and *trans* di-epoxides as mixtures of *syn*–*anti* diastereomers, and at least one pure diastereomer. Most pairs of enantiomers are resolved in the chiral GC fingerprints. Applied to three model catalysts in comparison to general Brønsted acid catalysis, distinct fingerprints were found for all catalysts as well as for all different diastereomers of the substrate.

In the *cis* series, most significant selectivity was observed with pnictogen-bonding catalysts, which give the unique AB product for *anti* and the more frequent BA product for the *syn* diastereomer of the diepoxide substrate with remarkably high selectivity. In the *trans* series, pnictogen-bonding catalysts broke the Baldwin rules most efficiently and independent of substrate stereochemistry, while within supramolecular capsules, completely new products were formed, including an interesting House–Meinwald rearrangement leading to cyclic hemiacetals. These distinct selectivities can be understood from the nature of the reactive intermediates. Together with particularly revealing details in the decoded product fingerprints, experimental support is obtained that the cascades are interconnected, possibly concerted. In clear contrast, anion–π catalysts gave mostly Baldwin products with fingerprints similar to general Brønsted acids. However, they showed unique autocatalytic behavior, a distinct emergent property that was independent of the stereochemistry of the substrate. All these distinctive characteristics found for representative supramolecular catalysts would be missed without the availability of decoded product fingerprints.

These results thus validate the existence and significance of privileged substrate systems as general chemistry tools to characterize supramolecular catalysis. Once established, decoded polyether cascade fingerprints are very easy to use, ready to serve the community. For a new supramolecular catalyst to be characterized, the decoded fingerprints will reveal unique differences compared to controls. Importantly, because the system is hyperresponsive (Fig. 1a and 2a), differences will be magnified. Due to the complexity required for hyperresponsiveness, the correlation of the fingerprint with the reactivity of a new catalyst will be mostly tentative and empirical at this point. For instance, AcOH-like fingerprints should reflect activation of epoxide opening to release the intramolecular leaving group, possibly supported by activation of the nucleophile as for autocatalysis on 10 (Fig. 5, IX). Fingerprints with more or even mostly A products should correlate with increasing S_N_1-like behavior. However, the generation of mostly B products with AcOH implies that the activation of epoxide opening needs to be supported by stabilization of the resulting carbocation with, *e.g.*, cation–π interactions to afford A products. With pnictogen-bonding catalyst 9, this would be meaningful on the π-basic tetrachlorocatecholate plane next to the σ hole stabilizing the alcoholate (Fig. 2d). In fingerprints with the new HM-rearrangement products, so far unique for capsules 8, the existence of carbocation intermediates is experimentally confirmed and thus presumably most relevant, due to cation–π interactions, confinement effects, or both. From here, with the system trained with more and more fingerprints, the correlation of fingerprint with mechanism of a new catalyst should become increasingly informative. Sooner or later, this will enable high-level computational simulations at high confidence,^7^ which in turn will enhance the information on reactivity available from fingerprints of new catalysts. According to preliminary results on the difference between pnictogen-bonding and Lewis acid catalysis^7^ and the elucidation of more complex supramolecular systems,^16^ these future perspectives are most promising.

## Experimental section

See ESI.†

## Data availability

Data for this paper are available at Zenodo at https://doi.org/10.5281/zenodo.6719513.

## Author contributions

H. C. and T.-R. L. performed all synthesis and catalysis, H. C., T.-R. L., N. S., C. B., L. G., M. P. and J. V.-G. contributed to product identification, N. S., K. T. and S. M. directed the study, all authors contributed to manuscript writing.

## Conflicts of interest

There are no conflicts to declare.

## Supplementary Material

SC-013-D2SC03991E-s001

SC-013-D2SC03991E-s002
