# Supplementary material for: Decoded fingerprints of hyperresponsive, expanding product space: polyether cascade cyclizations as tools to elucidate supramolecular catalysis
Source: Chem Sci. 2022 Aug 31;13(35):10273–80. doi: 10.1039/d2sc03991e (PMC9473502; doi:10.1039/d2sc03991e)
Supplement: SC-013-D2SC03991E-s001 [file SC-013-D2SC03991E-s001.pdf]

Electronic Supplementary Material (ESI) for Chemical Science.
This journal is © The Royal Society of Chemistry 2022

Electronic Supplementary Material (ESI) for Chemical Science.
This journal is © The Royal Society of Chemistry 2022

Supplementary information

Decoded fingerprints of hyperresponsive, expanding product space: Polyether cascade cyclizations as tools to elucidate supramolecular catalysis

Hao Chen,[a,b] Tian-Ren Li,[a,c] Naomi Sakai,[a,b] Celine Besnard,[b] Laure Guénée,[b] Marion Pupier,[b] Jasmine Viger-Gravel,[b] Konrad Tiefenbacher[a,c,d] and Stefan Matile[*a,b]

[a]National Centre of Competence in Research (NCCR) Molecular Systems Engineering, BPR 1095, Basel, Switzerland.

[b]Department of Organic Chemistry University of Geneva, Geneva, Switzerland.

[c]Department of Chemistry, University of Basel, Basel, Switzerland.

[d]Department of Biosystems Science and Engineering, ETH, Zurich, Basel, Switzerland.

[*]E-mail: stefan.matile@unige.ch

Table of contents

1. Materials and methods S3

2. Synthesis S4

   2.1. Synthesis of *cis* diepoxide substrates S5

   2.2. Synthesis of *trans* diepoxide substrates S8

3. Product identification S11

   3.1. Identification of BB products S11

   3.2. Identification of A-containing products S13

   3.3. Identification of products with acyclic and rearrangement motifs S20

4. Catalysis S22

   4.1. Catalysis with *cis* diepoxide substrates S22

      4.1.1. Brønsted-acid catalyst AcOH S24

      4.1.2. π-Basic capsule catalyst **8** S26

      4.1.3. Pnictogen-bonding catalyst **9** S28

      4.1.4. π-Acidic catalyst **10** S31

   4.2. Catalysis with *trans* diepoxide substrates S33

      4.2.1. Brønsted-acid catalyst AcOH S35

      4.2.2. π-Basic capsule catalyst **8** S37

      4.2.3. Pnictogen-bonding catalyst **9** S39

      4.2.4. π-Acidic catalyst **10** S41

5. Kinetics analysis S43

6. NMR spectra S49

7. X-ray crystallography S77

8. Supplementary references S89

1. Materials and methods

As in reference S1, Supplementary Information. Reagents for synthesis were purchased from Fluka, Sigma-Aldrich, Apollo Scientific and Acros. All solvents used in this study were passed through a 3.0 cm ALOX basic column to remove acidic impurities (such as HCl). Column chromatography was carried out on silica gel (SiliaFlash® P60, SILICYCLE, 230–400 mesh). Analytical (TLC) and preparative thin layer chromatography (PTLC) were performed on silica gel 60 F254 (Merck) and silica gel (SiliCycle, 1000 μm), respectively. Chiral Gas chromatography (GC) was performed on Agilent 6850 Series gas chromatographs equipped with a split-mode capillary injection system and flame ionization detectors using chiral stationary Hydrodex Gamma DiMOM column (50 m x 0.25 mm ID). Separation parameters: 60 °C, 1 °C/min, until 170 °C, then hold at 170 °C for 20 min (Speed: 60 cm/s H_2, injector temperature: 170 °C). ^{1}H and ^{13}C NMR spectra were recorded (as indicated) either on a Bruker 400 MHz or 500 MHz spectrometer and are reported as chemical shifts (δ) in ppm relative to TMS (δ = 0). Spin multiplicities are reported as a singlet (s), doublet (d), triplet (t) and quartet (q), with coupling constants (J) given in Hz, or multiplet (m). Broad peaks are marked as br. ^{1}H and ^{13}C resonances were assigned with the aid of additional information from 1D and 2D NMR spectra.

Abbreviations. A: anti-Baldwin; B: Baldwin; *m*-CPBA: *meta*-Chloroperoxybenzoic acid; DMAP: 4-Dimethylaminopyridine; EDTA: Ethylenediaminetetraacetic acid; GC-FID: Gas chromatography flame ionization detector; HM: House-Meinwald; HPLC: High-performance liquid chromatography; HSQC: Heteronuclear single quantum coherence spectroscopy; NMR: Nuclear magnetic resonance; rt: Room temperature; TBAF: Tetra-*n*-butylammonium fluoride; THF: Tetrahydrofuran; TMS: Tetramethylsilane.

2. Synthesis

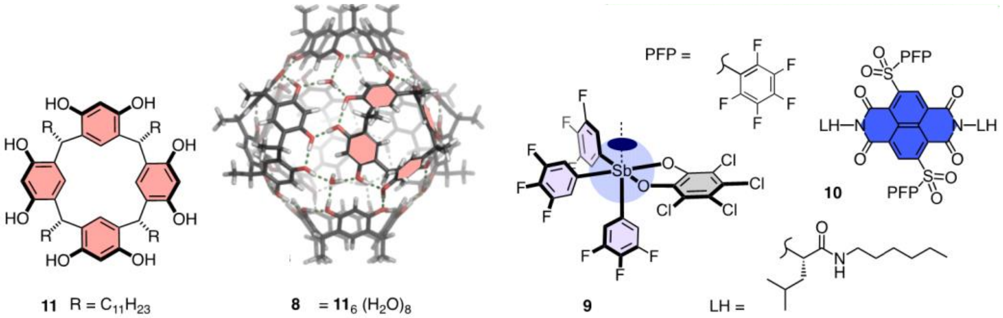

Figure S1. Structure of catalysts utilized in the di-epoxide opening.

Compound 9 was prepared following previously reported procedures.[S2]

Compound 10 was prepared following previously reported procedures.[S3]

Compound 11 was prepared following previously reported procedures.[S4]

Compound *cis*-14 was prepared following previously reported procedures.[S5]

Compounds *cis*-15, *cis*-16, *trans*-15 and ***trans*-16** were prepared following previously reported procedures.[S6]

Compounds *cis*-17 and ***trans*-17** were prepared following previously reported procedures.[S7]

Compound *cis*-1 was prepared following previously reported procedures.[S2]

Compound *trans*-1 was prepared following previously reported procedures.[S8]

2.1. Synthesis of *cis* diepoxide substrates

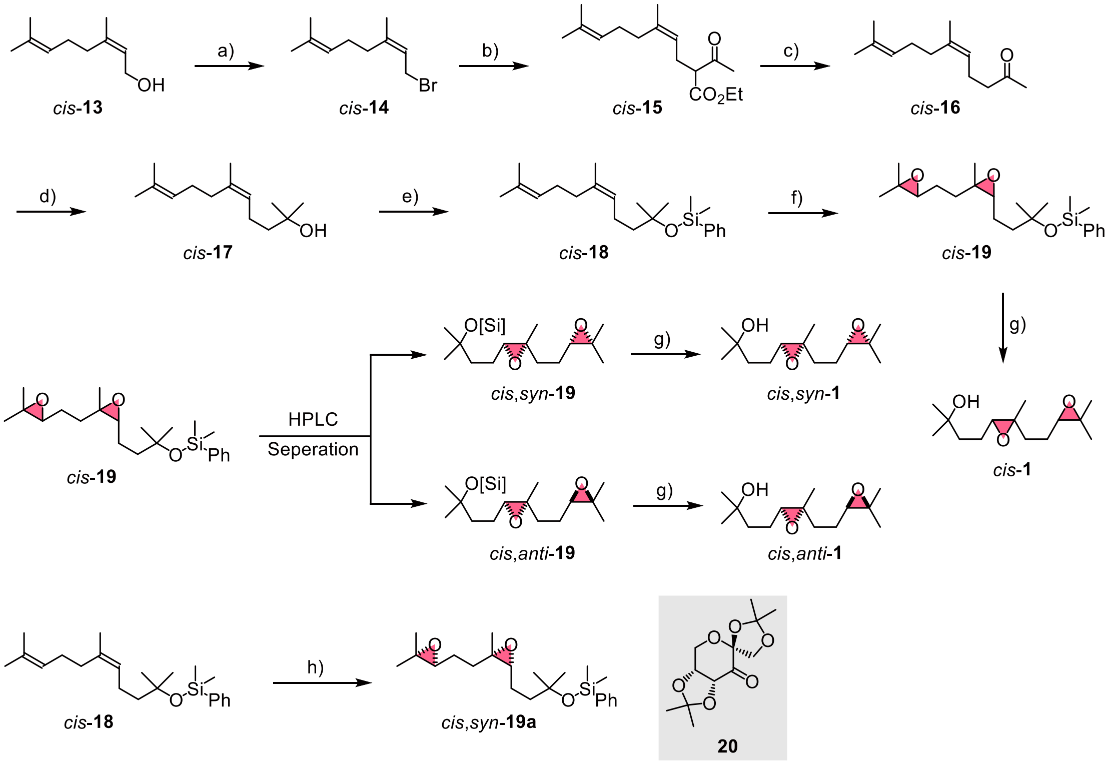

Scheme S1 (a) PBr_3, Et_2O, 0 ºC; (b) ethyl acetoacetate, NaH, THF, 0 ºC to rt, 46% (two steps); (c) NaOH (aq), EtOH, reflux, 52%; (d) MeMgBr, Et_2O, 0 ºC to rt, 98%; (e) $PhMe_2SiCl$, Et_3N, CH_2Cl_2, 0 ºC to rt, 73%; (f) *m*-CPBA, CH_2Cl_2, 0 ºC to rt, 85%; (g) TBAF, THF, 0 ºC to rt, 91% (***cis*-1**), 94% (***cis,syn*-1**), 91% (***cis,anti*-1**); (h) $Na_2B_4O_7$•$10H_2O$, Na_2EDTA, *n*-Bu_4NHSO_4, **20**, Oxone, K_2CO_3, $H_2O/MeOCH_2OMe/MeCN$ (10:6:3), *cis,syn*-**19a**, 82%, d.r. 89:11.

Compound *cis*-18. To a solution of *cis*-**17** (1.05 g, 5.00 mmol) in CH_2Cl_2 (20 mL) was added triethylamine (836 µL, 6.00 mmol) at 0 ºC, then chloro(dimethyl)phenylsilane (856 µL, 5.10 mmol) was slowly added into the solution and the resulting mixture was gradually warmed to rt after 10 min. The mixture was stirred at rt and stopped after 8 h when the complete

consumption of the starting material was observed. The crude mixture was purified by silica gel column chromatography directly (pentane/EtOAc 100:0 to 80:1) to give *cis*-**18** (1.25 g, 73%) as a light yellow oil. R_f (pentane/Et_2O 100:1): 0.8; ^{1}H NMR (500 MHz, $CDCl_3$): 7.60 – 7.59 (m, 2H), 7.35 – 7.34 (m, 3H), 5.12 – 5.09 (m, 2H), 2.08 – 2.04 (m, 6H), 1.68 (s, 6H), 1.60 (s, 3H), 1.48 – 1.45 (m, 2H), 1.21 (s, 6H), 0.37 (s, 6H); ^{13}C NMR (125 MHz, $CDCl_3$): 140.7 (C), 135.0 (C), 133.5 (CH), 131.6 (C), 129.2 (CH), 127.8 (CH), 125.7 (CH), 124.5 (CH), 74.7 (C), 45.2 (CH_3), 32.1 (CH_3), 30.0 ($2CH_2$), 26.8 (CH_3), 25.9 (CH_2), 23.6 (CH_2), 23.1 (CH_3), 1.6 (CH_3).

Compound *cis*-19. To a solution of *cis*-**18** (0.41 g, 1.2 mmol) in CH_2Cl_2 (15 mL) was added *m*-CPBA (70% purity, 1.3 g, 5.2 mmol) portionwisely at 0 ºC. The resulting mixture was gradually warmed to rt after 10 min. The mixture was stirred at rt and stopped after 2 h when the complete consumption of the starting material was observed. The crude mixture was purified by silica gel column chromatography directly (pentane/Et_2O 19:1 to 3:1) to give *cis*-**19** (0.38 g, 85%) as a light yellow oil. The two diastereomers *cis*,syn-**19** and *cis*,*anti*-**19** could be obtained by the separation of *cis*-**19** with preparative HPLC (CHIRALPAK® IA (20 mm ø x 250 mmL), 12.8 mL/min, hexane/Et_2O 4:1). R_f (pentane/Et_2O 3:1): 0.5; ^{1}H NMR (500 MHz, $CDCl_3$): 7.61 – 7.50 (m, 2H), 7.38 – 7.31 (m, 3H), 2.77 – 2.56 (m, 2H), 1.73 – 1.58 (m, 7H), 1.56 – 1.50 (m, 1H), 1.30 (s, 3H), 1.281 (s, 3H), 1.275 (s, 3H), 1.22 (s, 3H), 1.21 (s, 3H), 0.37 (s, 6H); ^{13}C NMR (125 MHz, $CDCl_3$): 141.1 (C), 133.4 (CH), 129.3 (CH), 127.8 (CH), 74.3 (C), 65.3 (CH), 64.2 (CH), 60.7 (C), 58.6 (C), 41.4 (CH_2), 30.2 (CH_3), 29.8 (CH_3), 29.6 (CH_2), 25.2 (CH_2), 25.0 (CH_3), 23.8 (CH_2), 22.4 (CH_3), 18.8 (CH_3), 1.54 (CH_3), 1.52 (CH_3).

Shi epoxidation. Compound *cis*-**18** (856 mg, 2.48 mmol) was dissolved in a mixture of $MeOCH_2OMe$/MeCN (2:1, 37.2 mL). A 0.05 M solution of $Na_2B_4O_7 \cdot 10H_2O$ (in 4×10^{-4} M

aqueous solution of Na_2EDTA, 17.4 mL), *n*-Bu_4NHSO_4 (55.6 mg, 0.160 mmol) and **20** (384 mg, 1.49 mmol) were sequentially added under vigorous stirring at 0 °C. To this mixtures solution of Oxone (3.66 g, 11.9 mmol, in 4×10^{-4} M aqueous solution of Na_2EDTA, 12.4 mL), and K_2CO_3 (3.43 g, 24.8 mmol, in water (12.4 mL)), were simultaneously added over 1 h via syringe pump. At this point, the mixture was diluted with water (20 mL), and extracted with EtOAc (3 x 30 mL). The combined organic layers were washed with brine, dried over Na_2SO_4, and purified by flash column chromatography (pentane/Et_2O 19:1 to 3:1) to give *cis*,*syn*-**19a** (0.77 g, 82%, d.r. 89:11) as a light yellow oil. The diastereoselectivity of compound *cis*,*syn*-**19a** could be improved to >20:1 after purification with preparative HPLC (CHIRALPAK® ID (10 mm ø x 250 mmL), 3.2 mL/min, hexane/Et_2O 6:1).

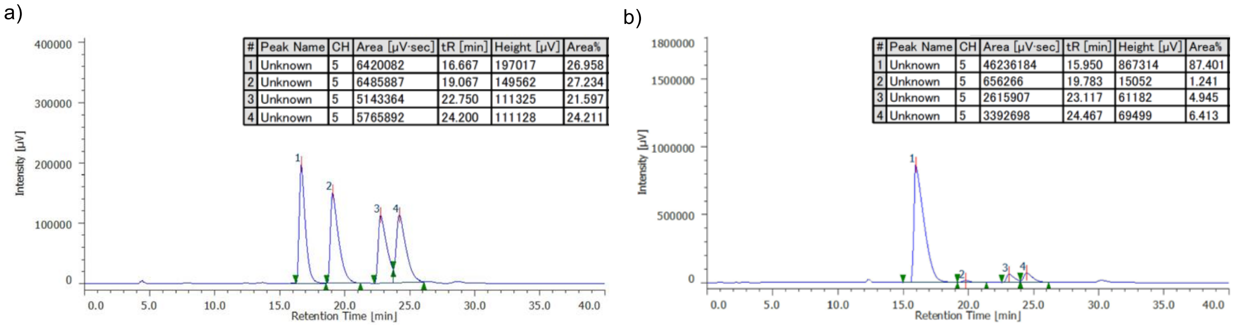

Figure S2. HPLC chromatograms of a) *cis*-**19** and b) *cis*,*syn*-**19a** (CHIRALPAK® ID (4.6 mm ø x 250 mmL), 0.8 mL/min, hexane/Et_2O 6:1).

2.2. Synthesis of *trans* diepoxide substrates

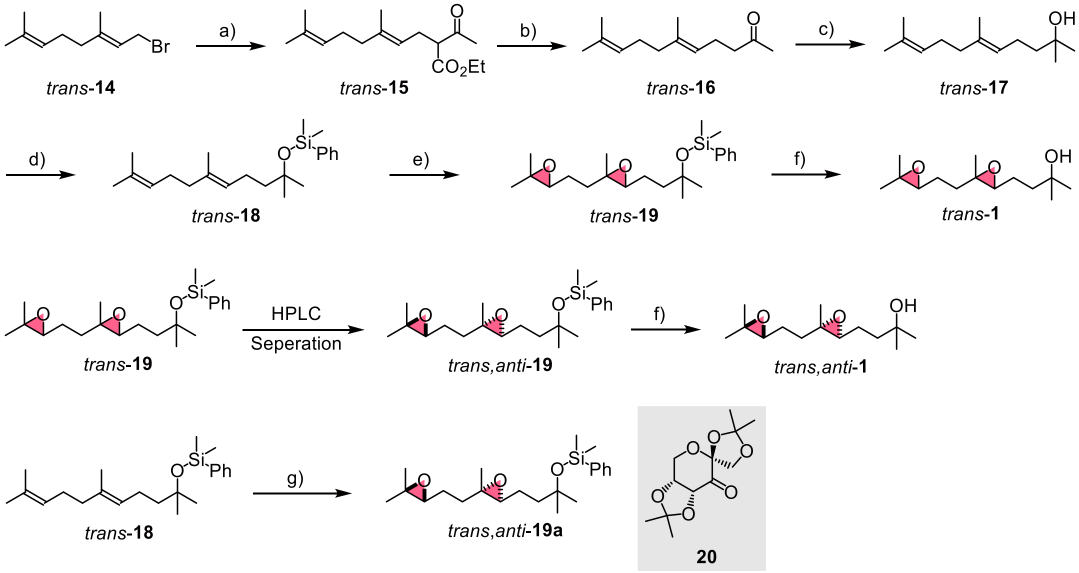

Scheme S2 (a) Ethyl acetoacetate, NaH, THF, 0 ºC to rt, 66%; (b) NaOH (aq), EtOH, reflux, 79%; (c) MeMgBr, Et_2O, 0 ºC to rt, 99%; (d) $PhMe_2SiCl$, Et_3N, CH_2Cl_2, 0 ºC to rt, 68%; (e) *m*-CPBA, CH_2Cl_2, 0 ºC to rt, 88%; (f) TBAF, THF, 0 ºC to rt, 91% (*trans*-**1**), 90% (*trans,anti*-**1**); (g) $Na_2B_4O_7$•$10H_2O$, Na_2EDTA, *n*-Bu_4NHSO_4, **20**, Oxone, K_2CO_3, $H_2O/MeOCH_2OMe/MeCN$ (10:6:3), *trans,anti*-**19a**, 67%, d.r. 82:18.

Compound *trans*-18. To a solution of *trans*-**17** (1.0 g, 4.9 mmol) in CH_2Cl_2 (20 mL) was added triethylamine (818 µL, 5.87 mmol) at 0 ºC, then chloro(dimethyl)phenylsilane (837 µL, 5.00 mmol) was slowly added into the solution and the resulting mixture was gradually warmed to rt after 10 min. The mixture was stirred at rt and stopped after 8 h when the complete consumption of the starting material was observed. The crude mixture was purified by silica gel column chromatography directly (pentane/EtOAc 100:0 to 80:1) to give *trans*-**18** (1.15 g, 68%) as a light yellow oil. R_f (pentane/Et_2O 100:1): 0.8; ^1H NMR (500 MHz, $CDCl_3$): 7.63 – 7.56 (m,

2H), 7.38 – 7.31 (m, 3H), 5.15 – 5.03 (m, 2H), 2.11 – 2.02 (m, 4H), 2.00 – 1.95 (m, 2H), 1.68 (d, $^4J_{H\text{-}H}$ = 1.4 Hz, 3H), 1.60(5) (s, 3H), 1.60(0) (s, 3H), 1.50 – 1.40 (m, 2H), 1.21 (s, 6H), 0.38 (s, 6H); ^{13}C NMR (125 MHz, $CDCl_3$): 140.8 (C), 134.9 (C), 133.5 (CH), 131.4 (C), 129.2 (CH), 127.7 (CH), 124.9 (CH), 124.6 (CH), 74.7 (C), 44.9 (CH_2), 39.9 (CH_2), 30.0 (CH_3), 26.9 (CH_2), 25.9 (CH_3), 23.2 (CH_2), 17.8 (CH_3), 16.1 (CH_3), 1.6 (CH_3).

Compound *trans*-19. To a solution of *trans*-**18** (187 mg, 0.543 mmol) in CH_2Cl_2 (5 mL) was added *m*-CPBA (70% purity, 589 mg, 2.39 mmol) portionwisely at 0 ºC. The resulting mixture was gradually warmed to rt after 10 min. The mixture was stirred at rt and stopped after 2 h when the complete consumption of the starting material was observed. The crude mixture was purified by silica gel column chromatography directly (pentane/Et_2O 19:1 to 3:1) to give *trans*-**19** (0.18 g, 88%) as a light yellow oil. The two diastereomers *trans*,syn-**19** and *trans*,*anti*-**19** could be obtained by the separation of *trans*-**19** with preparative HPLC (CHIRALPAK® IA (20 mm ø x 250 mmL), 12.8 mL/min, hexane/Et_2O 4:1). R_f (pentane/Et_2O 3:1): 0.5; ^{1}H NMR (500 MHz, $CDCl_3$): 7.60 – 7.52 (m, 2H), 7.38 – 7.32 (m, 3H), 2.75 – 2.65 (m, 2H), 1.82 – 1.74 (m, 1H), 1.70 – 1.57 (m, 5H), 1.55 – 1.46 (m, 2H), 1.31 (s, 3H), 1.27 (s, 3H), 1.26 (s, 3H), 1.22 (s, 3H), 1.21 (s, 3H); ^{13}C NMR (125 MHz, $CDCl_3$): 140.4 (C), 133.4 (CH), 129.3 (CH), 127.8 (CH), 74.2 (C), 64.0 (CH), 63.5 (CH), 60.5 (C), 58.6 (C), 41.3 (CH_2), 35.4 (CH_2), 30.2 (CH_3), 29.8 (CH_3), 25.0 (CH_3), 24.8 (CH_2), 24.0 (CH_2), 18.8 (CH_3), 16.7 (CH_3), 1.5 (CH_3).

Shi epoxidation. Compound *trans*-**18** (856 mg, 2.48 mmol) was dissolved in a mixture of $MeOCH_2OMe$/MeCN (2:1, 37.2 mL). A 0.05 M solution of $Na_2B_4O_7\cdot10H_2O$ (in 4×10^{-4} M aqueous solution of Na_2EDTA, 17.4 mL), *n*-Bu_4NHSO_4 (55.6 mg, 0.160 mmol) and **20** (384 mg, 1.49 mmol) were sequentially added under vigorous stirring at 0 °C. To this mixtures solution of

Oxone (3.66 g, 11.9 mmol, in 4×10^{-4} M aqueous solution of Na_2EDTA, 12.4 mL), and K_2CO_3 (3.43 g, 24.8 mmol, in water (12.4 mL)), were simultaneously added over 1 h via syringe pump. At this point, the mixture was diluted with water (20 mL), and extracted with EtOAc (3 x 30 mL). The combined organic layers were washed with brine, and dried over Na_2SO_4, purified by flash column chromatography (pentane/Et_2O 19:1 to 3:1) to give *trans*,*anti*-**19a** (0.63 g, 67%, d.r. 82:18) as a light yellow oil. The diastereoselectivity of compound *trans*,*anti*-**19a** could be improved to >20:1 after purification with preparative HPLC (CHIRALPAK® ID (10 mm ø x 250 mmL), 3.2 mL/min, hexane/Et_2O 6:1).

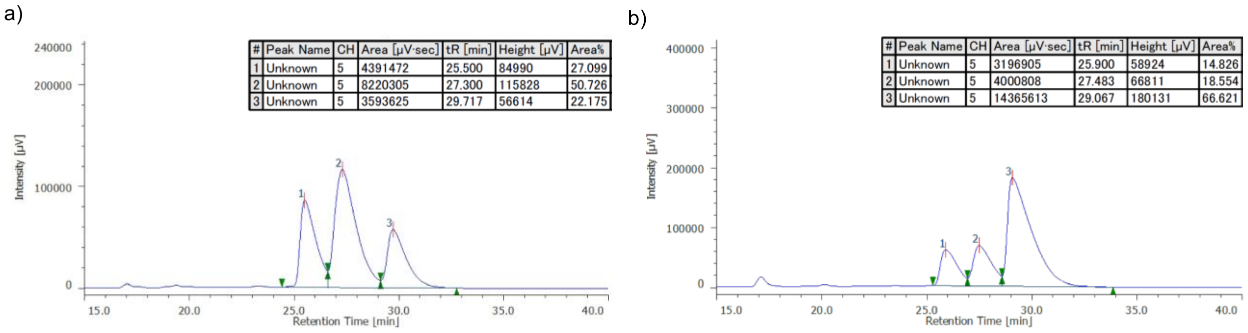

Figure S3. HPLC chromatograms of a) *trans*-**19** and b) *trans*,*anti*-**19a** (CHIRALPAK® ID (4.6 mm ø x 250 mmL), 0.8 mL/min, hexane/Et_2O 6:1).

3. Product identification

3.1. Identification of BB products

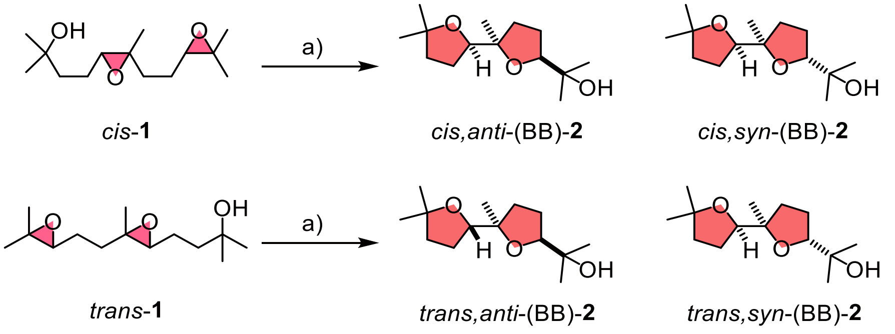

Scheme S3 (a) AcOH, CH_2Cl_2, 40 °C, 48 h.

Compounds *cis,anti*-(BB)-2 and *cis,syn*-(BB)-2.[S7] To a solution of *cis*-**1** (243 mg, 1.00 mmol) in CH_2Cl_2 (4.0 mL) was added AcOH (57.5 µL, 1.00 mmol), then the solution was heated to 40 °C. The reaction was monitored by ^{1}H NMR spectroscopy and stopped after 48 h when the complete consumption of the starting material was observed. The crude mixture was purified by silica gel column chromatography directly (pentane/Et_2O 19:1 to 4:1) to give *cis,anti*-(BB)-**2** *and cis-syn*-(BB)-**2** as light yellow oils. ***cis,anti*-(BB)-2.** R_f (pentane/Et_2O 1:1): 0.6; ^{1}H NMR (500 MHz, CD_2Cl_2): 3.86 – 3.80 (m, 2H), 2.15 – 2.10 (m, 1H), 2.00 – 1.85 (m, 4H), 1.74 – 1.71 (m, 2H), 1.56 – 1.51 (m, 1H), 1.25 (s, 3H), 1.20 (s, 3H), 1.19 (s, 3H), 1.11 (s, 3H), 1.02 (s, 3H); ^{13}C NMR (125 MHz, CD_2Cl_2): 85.9 (CH), 83.9 (C), 83.6 (CH), 81.3 (C), 72.1 (C), 38.9 (CH_2), 34.8 (CH_2), 28.9 (CH_3), 28.1 (CH_3), 27.9 (CH_3), 27.6 (CH_2), 26.1 (CH_2), 25.2 (CH_3), 24.1 (CH_3). ***cis,syn*-(BB)-2.** R_f (pentane/Et_2O 1:1): 0.57; ^{1}H NMR (500 MHz, CD_2Cl_2): 3.88 – 3.85 (m, 1H), 3.79 – 3.76 (m, 1H), 2.12 (s, 1H), 2.05 – 1.99 (m, 1H), 1.94 – 1.84 (m, 2H), 1.80 – 1.75 (m, 2H), 1.72 – 1.68 (m, 2H), 1.62 – 1.58 (m, 1H), 1.21(2) (s, 3H), 1.20(6) (s, 3H), 1.17 (s, 3H), 1.10 (s,

3H), 1.07 (s, 3H); ^{13}C NMR (125 MHz, CD_2Cl_2): 87.5 (CH), 84.9 (CH), 84.4 (C), 81.3 (C), 70.6 (C), 39.0 (CH_2), 35.0 (CH_2), 28.6 (CH_3), 28.2 (CH_3), 28.0 (CH_3), 27.8 (CH_2), 26.7 (CH_2), 24.4 (CH_3), 23.8 (CH_3).

Compounds *trans,anti*-(BB)-2 and *trans,syn*-(BB)-2.[S7] To a solution of *trans*-**1** (243 mg, 1.00 mmol) in CH_2Cl_2 (4.0 mL) was added AcOH (57.5 µL, 1.00 mmol), then the solution was heated to 40 °C. The reaction was monitored by ^1H NMR spectroscopy and stopped after 48 h when the complete consumption of the starting material was observed. The crude mixture was purified by silica gel column chromatography directly (pentane/Et_2O 19:1 to 4:1) to give *trans,anti*-(BB)-**2** *and trans-syn*-(BB)-**2** as light yellow oils. ***trans,anti*-(BB)-2.** R_f (pentane/Et_2O 1:1): 0.59; ^1H NMR (500 MHz, CD_2Cl_2): 4.00 (dd, $^3J_{H\text{-}H}$ = 8.9, 6.5 Hz, 1H), 3.80 (dd, $^3J_{H\text{-}H}$ = 7.7, 5.2 Hz, 1H), 3.77 (brs, 1H), 2.12 – 1.99 (m, 2H), 1.97 – 1.85 (m, 2H), 1.79 – 1.67 (m, 2H), 1.63 – 1.56 (m, 1H), 1.55 – 1.47 (m, 1H), 1.23 – 1.21 (m, 6H), 1.16 (s, 3H), 1.11 (s, 3H), 1.01 (s, 3H); ^{13}C NMR (125 MHz, CD_2Cl_2): 86.0 (CH), 85.7 (C), 84.5 (CH), 81.4 (C), 71.9 (C), 38.9 (CH_2), 31.6 (CH_2), 29.0 (CH_2), 28.6 (CH_3), 28.2 (CH_3), 28.1 (CH_3), 26.7 (CH_2), 25.4 (CH_3), 24.8 (CH_3). ***trans,syn*-(BB)-2.** R_f (pentane/Et_2O 1:1): 0.61; ^1H NMR (400 MHz, CD_2Cl_2): 3.90 (t, $^3J_{H\text{-}H}$ = 7.1 Hz, 1H), 3.74 (t, $^3J_{H\text{-}H}$ = 7.8 Hz, 1H), 2.02 – 1.88 (m, 2H), 1.83 – 1.77 (m, 2H), 1.76 – 1.66 (m, 3H), 1.65 – 1.58 (m, 1H), 1.55 (brs, 1H), 1.22 (s, 3H), 1.21 (s, 3H), 1.16 (s, 3H), 1.12 (s, 3H), 1.07 (s, 3H); ^{13}C NMR (100 MHz, CD_2Cl_2): 87.3 (CH), 85.0 (C), 84.7 (CH), 81.2 (C), 70.8 (C), 38.9 (CH_2), 34.2 (CH_2), 28.8 (CH_3), 28.2 (CH_2), 28.1 (CH_3), 27.8 (CH_3), 26.8 (CH_2), 24.3 (CH_3), 23.8 (CH_3).

3.2. Identification of A-containing products

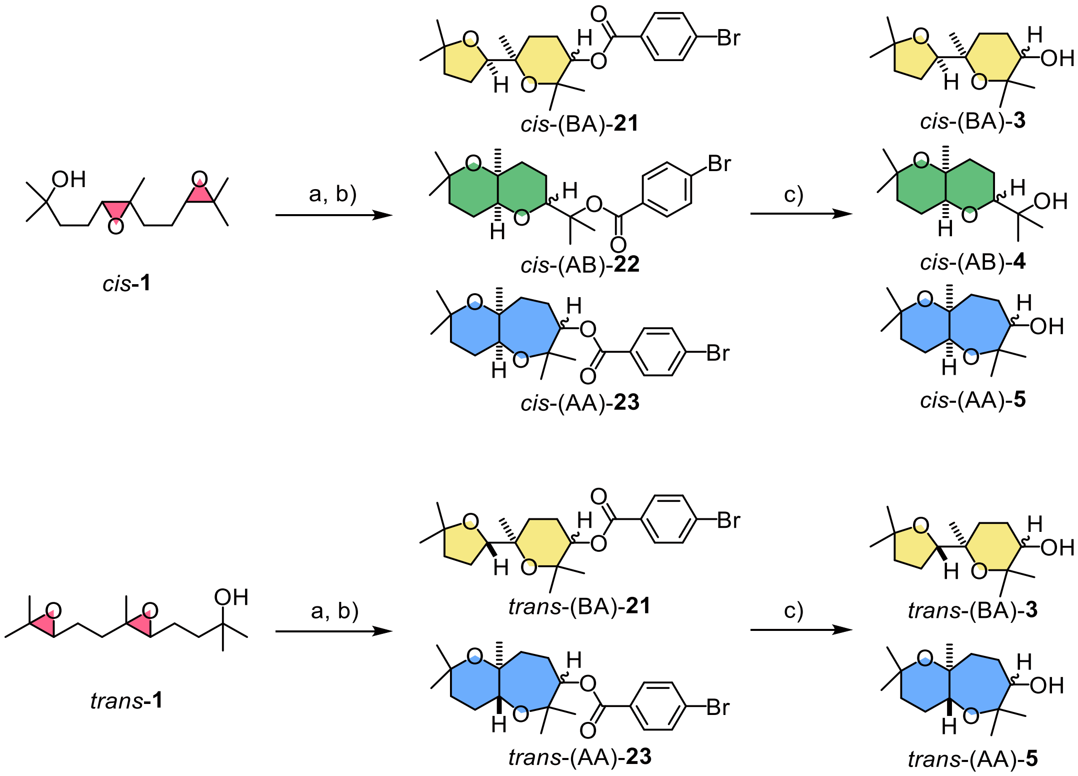

Scheme S4 (a) **9**, CH_2Cl_2, rt, 1 h; (b) 4-bromobenzoyl chloride, DMAP, Et_3N, CH_2Cl_2, 0 ºC to rt, 72 h; (c) K_2CO_3, MeOH/CH_2Cl_2 (1:1), rt, 48 h, 68% (*cis*,*anti*-(BA)-**3**), 75% (*cis*,*syn*-(BA)-**3**), 76% (*cis*,*anti*-(AB)-**4**), 51% (*cis*,*syn*-(AA)-**5**), 76% (*trans*,*anti*-(BA)-**3**), 74% (*trans*,*syn*-(BA)-**3**) and 79% (*trans*,*syn*-(AA)-**5**).

Compounds *cis*-(BA)-21, *cis*-(AB)-22 and *cis*-(AA)-23. To a solution of *cis*-**1** (0.10 g, 0.41 mmol) in CH_2Cl_2 (3.33 mL) was added catalyst **9** (7.85 mg, 10.3 µmol) at rt. The reaction was monitored by ^{1}H NMR spectra and stopped until full consumption of the starting material after 1 h. The resulting solution was cooled down to 0 ºC, then Et_3N (345 µL, 2.48 mmol) and DMAP (302 mg, 2.48 mmol) were added to the solution successively, followed by the addition

of 4-bromobenzoyl chloride (906 mg, 4.13 mmol). The reaction was stirred for 72 h, the crude mixture was first purified by silica gel column chromatography (pentane/Et_2O 19:1 to 3:2) and then further purified with preparative HPLC (CHIRALPAK® IA (20 mm ø x 250 mmL), 12.8 mL/min, pentane/Et_2O 19:1) to give *cis,anti*-(BA)-**21**, *cis,syn*-(BA)-**21**, *cis,anti*-(AB)-**22** *and* *cis,syn*-(AA)-**23** as colorless solids. Structures of *cis,anti*-(AB)-**22** and *cis,syn*-(AA)-**23** were determined by X-ray crystallography (crystal growth conditions: hexane/Et_2O 10:1, rt). ***cis,anti*-(BA)-21.** R_f (pentane/Et_2O 6:1): 0.6; ^{1}H NMR (400 MHz, CD_2Cl_2): 7.99 – 7.96 (m, 2H), 7.63 – 7.60 (m, 2H), 4.94 – 4.92 (m, 1H), 3.81 (t, $^3J_{H\text{-}H}$ = 7.3 Hz, 1H), 2.22 – 2.14 (m, 1H), 1.99 – 1.84 (m, 4H), 1.71 – 1.67 (m, 2H), 1.34 (s, 3H), 1.24(2) (s, 3H), 1.23(5) (s, 3H), 1.21 (s, 3H), 1.16 (s, 3H); ^{13}C NMR (100 MHz, CD_2Cl_2): 165.3 (C), 132.1 (CH), 131.6 (CH), 130.2 (C), 128.2 (C), 87.2 (CH), 81.2 (C), 75.0 (C), 73.5 (CH), 73.3 (C), 38.8 (CH_2), 28.9 (CH_3), 28.1 (CH_3), 27.9 (CH_3), 27.7 (CH_3), 26.9 (CH_2), 24.8 (CH_2), 23.0 (CH_3), 21.3 (CH_2). ***cis,syn*-(BA)-21.** R_f (pentane/Et_2O 6:1): 0.6; ^{1}H NMR (500 MHz, CD_2Cl_2): 7.89 – 7.86 (m, 2H), 7.62 – 7.59 (m, 2H), 4.80 (dd, $^3J_{H\text{-}H}$ = 10.8, 4.6 Hz, 1H), 3.75 (t, $^3J_{H\text{-}H}$ = 7.4 Hz, 1H), 1.98 – 1.77 (m, 5H), 1.69 – 1.66 (m, 2H), 1.53 – 1.47 (m, 1H), 1.39 (s, 3H), 1.23 (s, 3H), 1.22 (s, 3H), 1.20 (s, 3H), 1.18 (s, 3H); ^{13}C NMR (125 MHz, CD_2Cl_2): 164.9 (C), 132.1 (CH), 131.4 (CH), 130.0 (C), 128.2 (C), 86.3 (CH), 81.2 (C), 77.9 (CH), 75.3 (C), 73.6 (C), 38.8 (CH_2), 30.6 (CH_2), 30.1 (CH_3), 28.8 (CH_3), 28.0 (CH_3), 27.0 (CH_2), 23.4 (CH_3), 22.5 (CH_3), 22.0 (CH_2). ***cis,anti*-(AB)-22.** R_f (pentane/Et_2O 6:1): 0.65; ^{1}H NMR (500 MHz, CD_2Cl_2): 7.86 – 7.83 (m, 2H), 7.57 – 7.55 (m, 2H), 3.70 – 3.58 (m, 1H), 3.32 (dd, $^3J_{H\text{-}H}$ = 3.7, 2.2 Hz, 1H), 2.05 – 1.95 (m, 1H), 1.93 – 1.85 (m, 1H), 1.82 – 1.71 (m, 2H), 1.66 – 1.62 (m, 1H), 1.60 (s, 3H), 1.57 (s, 3H), 1.44 – 1.39 (m, 2H), 1.25 – 1.21 (m, 4H), 1.18(4) (s, 3H), 1.17(9) (s, 3H); ^{13}C NMR (125 MHz, CD_2Cl_2): 165.0 (C), 131.9 (CH),

131.6 (C), 131.5 (CH), 127.6 (C), 84.8 (C), 81.0 (CH), 74.8 (CH), 71.4 (C), 69.3 (C), 39.2 (CH_2), 33.6 (CH_3), 30.5 (CH_2), 28.5 (CH_3), 27.0 (CH_3), 23.1 (CH_3), 22.9 (CH_2), 21.8 (CH_3), 21.7 (CH_2). ***cis,syn*-(AA)-23.** R_f (pentane/Et_2O 6:1): 0.55; ^1H NMR (500 MHz, CD_2Cl_2): 7.87 – 7.84 (m, 2H), 7.61 – 7.59 (m, 2H), 4.85 (dd, $^3J_{H-H}$ = 10.8, 3.4 Hz, 1H), 3.54 (t, $^3J_{H-H}$ = 3.6 Hz, 1H), 2.06 – 1.97 (m, 1H), 1.87 – 1.82 (m, 1H), 1.79 – 1.62 (m, 3H), 1.61 – 1.54 (m, 2H), 1.31 – 1.27 (m, 4H), 1.23 (s, 3H), 1.20 (s, 6H), 1.17 (m, 3H); ^{13}C NMR (125 MHz, CD_2Cl_2): 165.1 (C), 132.8 (CH), 131.4 (CH), 130.1 (C), 128.2 (C), 81.7 (CH), 76.8 (C), 76.4 (C), 71.2 (C), 69.4 (CH), 38.7 (CH_2), 33.3 (CH_3), 30.2 (CH_3), 27.8 (CH_3), 27.1 (CH_3), 24.9 (CH_2), 24.7 (CH_2), 19.3 (CH_3).

Compounds *cis*-(BA)-3, *cis*-(AB)-4 and *cis*-(AA)-5. The above obtained compounds *cis,anti*-(BA)-**21**, *cis,syn*-(BA)-**21**, *cis,anti*-(AB)-**22** and *cis,syn*-(AA)-**23** were dissolved in MeOH/CH_2Cl_2 (1:1, 25 mM) in separated vials, then corresponding amounts of K_2CO_3 (5 equiv) were added to each vials and the reactions were stirred at rt. The mixture was stirred at rt and stopped after 48 h when the complete consumption of the starting material was observed. The crude mixture was purified by silica gel column chromatography directly (pentane/Et_2O 19:1 to 2:1) to give *cis,anti*-(BA)-**3** (1.55 mg, 68%), *cis,syn*-(BA)-**3** (3.03 mg, 75%), *cis,anti*-(AB)-**4** (2.84 mg, 76%) *and cis,syn*-(AA)-**5** (1.29 mg, 51%) as light yellow oils. ***cis,anti*-(BA)-3.** R_f (pentane/Et_2O 1:1): 0.5; ^1H NMR (500 MHz, CD_2Cl_2): 3.68 (t, $^3J_{H-H}$ = 7.5 Hz, 1H), 3.38 – 3.25 (m, 1H), 2.06 – 1.93 (m, 3H), 1.84 – 1.75 (m, 1H), 1.74 – 1.64 (m, 4H), 1.24 (s, 3H), 1.22 (s, 3H), 1.20 (s, 3H), 1.16 (s, 3H), 1.11 (s, 3H); ^{13}C NMR (125 MHz, CD_2Cl_2): 87.6 (CH), 81.1 (C), 75.8 (C), 73.7 (C), 70.3 (CH), 39.0 (CH_2), 28.7 (CH_3), 28.1 (CH_3), 27.5 (CH_3), 27.4 (CH_3), 26.1 (CH_2), 24.7 (CH_3), 24.7 (CH_2), 23.5 (CH_2). ***cis,syn*-(BA)-3.** R_f (pentane/Et_2O 1:1): 0.5; ^1H NMR (400 MHz, CD_2Cl_2): 3.68 (t, $^3J_{H-H}$ = 7.3 Hz, 1H), 3.34 (t, $^3J_{H-H}$ = 7.9 Hz, 1H), 1.86 – 1.77 (m,

2H), 1.73 – 1.67 (m, 2H), 1.66 – 1.63 (m, 3H), 1.47 – 1.37 (m, 2H), 1.20 – 1.17 (m, 12H), 1.15 (s, 3H); ^{13}C NMR (100 MHz, CD_2Cl_2): 86.6 (CH), 81.1 (C), 75.6 (CH), 75.1 (C), 74.9 (C), 38.8 (CH_2), 31.2 (CH_2), 30.1 (CH_3), 28.8 (CH_3), 28.0 (CH_3), 27.0 (CH_2), 25.4 (CH_2), 22.3 (CH_3), 21.8 (CH_3). ***cis,anti*-(AB)-4.** R_f (pentane/Et_2O 1:1): 0.6; ^{1}H NMR (400 MHz, CD_2Cl_2): 3.31 (dd, $^3J_{H\text{-}H}$ = 3.7, 2.2 Hz, 1H), 3.09 (dd, $^3J_{H\text{-}H}$ = 11.5, 1.8 Hz, 1H), 2.61 (brs, 1H), 2.09 – 1.95 (m, 1H), 1.91 – 1.81 (m, 1H), 1.79 – 1.72 (m, 1H), 1.72 – 1.64 (m, 1H), 1.62 – 1.56 (m, 1H), 1.39 – 1.28 (m, 3H), 1.23 (s, 3H), 1.17 (s, 3H), 1.15 (s, 3H), 1.14 (s, 3H), 1.11 (s, 3H); ^{13}C NMR (100 MHz, CD_2Cl_2): 83.6 (CH), 74.6 (CH), 72.2 (C), 71.4 (C), 69.3 (C), 39.2 (CH_2), 33.6 (CH_3), 30.5 (CH_2), 28.5 (CH_3), 27.0 (CH_3), 25.8 (CH_3), 24.0 (CH_3), 22.9 (CH_2), 22.3 (CH_2). ***cis,syn*-(AA)-5.** R_f (pentane/Et_2O 1:1): 0.4; ^{1}H NMR (400 MHz, CD_2Cl_2): 3.44 – 3.35 (m, 2H), 2.04 – 1.89 (m, 1H), 1.74 – 1.63 (m, 2H), 1.63 – 1.56 (m, 3H), 1.53 – 1.38 (m, 3H), 1.25 (s, 3H), 1.18 – 1.15 (m, 6H), 1.13 (s, 3H), 1.11 (s, 3H); ^{13}C NMR (100 MHz, CD_2Cl_2): 80.5 (CH), 77.6 (C), 77.0 (C), 71.9 (C), 68.9 (CH), 39.2 (CH_2), 33.3 (CH_3), 30.4 (CH_3), 30.3 (CH_2), 28.6 (CH_2), 27.6 (CH_3), 27.0 (CH_3), 24.9 (CH_2), 17.8 (CH_3).

Compounds *trans,anti*-(AA)-5,[S8] ***trans*-(BA)-21 and *trans*-(AA)-23.** To a solution of *trans*-**1** (0.10 g, 0.41 mmol) in CH_2Cl_2 (3.33 mL) was added catalyst **9** (7.85 mg, 10.3 μmol) at rt. The reaction was monitored by ^{1}H NMR spectroscopy and stopped after 1 h when the complete consumption of the starting material was observed. The crude mixture was first purified by silica gel column chromatography (pentane/Et_2O 19:1 to 3:2) to give *trans,anti*-(AA)-**5** as colorless solid and inseparable mixture of *trans*-(BA)-**3** and *trans*-(AA)-**4**. The mixture was dissolved in CH_2Cl_2 (3 mL) and cooled down to 0 ºC, then Et_3N (345 μL, 2.48 mmol) and DMAP (302 mg, 2.48 mmol) were added to the solution successively, followed by

the addition of 4-bromobenzoyl chloride (906 mg, 4.13 mmol). The reaction was stirred for 72 h, the crude mixture was first purified by silica gel column chromatography (pentane/Et_2O 19:1 to 3:2) and then was further purified with preparative HPLC (CHIRALPAK® IA (20 mm ø x 250 mmL), 12.8 mL/min, pentane/Et_2O 19:1) to give *trans,anti*-(BA)-**21**, *trans,syn*-(BA)-**21** and *trans,syn*-(AA)-**23** as colorless solids. Structures of *trans,anti*-(AA)-**5**, *trans,anti*-(BA)-**21**, *trans,syn*-(BA)-**21** and *trans,syn*-(AA)-**23** were determined by X-ray crystallography (crystal growth conditions: hexane/Et_2O = 10:1, rt). ***trans,anti*-(AA)-5.** R_f (pentane/Et_2O 1:1): 0.4; ^{1}H NMR (500 MHz, CD_2Cl_2): 3.77 (d, $^3J_{H-H}$ = 6.3 Hz, 1H), 3.59 (dd, $^3J_{H-H}$ = 11.6, 4.5 Hz, 1H), 1.81 – 1.71 (m, 3H), 1.67 (brs, 1H), 1.62 – 1.56 (m, 2H), 1.54 – 1.44 (m, 2H), 1.39 – 1.35 (m, 1H), 1.23 (s, 3H), 1.22 (s, 3H), 1.18 (s, 3H), 1.11 (s, 3H), 1.06 (s, 3H); ^{13}C NMR (125 MHz, CD_2Cl_2): 78.4 (C), 77.0 (C), 76.7 (CH), 73.8 (CH), 71.2 (C), 37.7 (CH_2), 36.7 (CH_2), 33.4 (CH_3), 28.9 (CH_3), 27.4 (CH_3), 25.9 (CH_2), 25.5 (CH_2), 22.3 (CH_3), 20.2(CH_3). ***trans,anti*-(BA)-21.** R_f (pentane/Et_2O 6:1): 0.6; ^{1}H NMR (500 MHz, CD_2Cl_2): 7.95 – 7.93 (m, 2H), 7.63 – 7.60 (m, 2H), 4.93 – 4.91 (m, 1H), 3.89 (t, $^3J_{H-H}$ = 7.2 Hz, 1H), 2.15 – 2.08 (m, 1H), 2.02 – 1.93 (m, 2H), 1.92 – 1.84 (m, 2H), 1.73 – 1.63 (m, 2H), 1.42 – 1.37 (m, 1H), 1.31 (s, 3H), 1.23 (s, 3H), 1.21 (s, 3H), 1.18 (s, 3H), 1.16 (s, 3H); ^{13}C NMR (125 MHz, CD_2Cl_2): 165.3 (C), 132.1 (CH), 131.5 (CH), 130.1 (C), 128.2 (C), 85.6 (CH), 81.3 (C), 75.0 (C), 74.1 (CH), 73.3 (C), 38.8 (CH_2), 28.7 (CH_3), 28.3 (CH_3), 27.8 (CH_3), 27.6 (CH_2), 27.1 (CH_3), 27.0 (CH_2), 22.3 (CH_3), 21.3 (CH_2). ***trans,syn*-(BA)-21.** R_f (pentane/Et_2O 6:1): 0.6; ^{1}H NMR (500 MHz, CD_2Cl_2): 7.89 – 7.87 (m, 2H), 7.62 – 7.59 (m, 2H), 4.82 (dd, $^3J_{H-H}$ = 10.2, 4.3 Hz, 1H), 3.79 (t, $^3J_{H-H}$ = 7.1 Hz, 1H), 2.00 – 1.84 (m, 4H), 1.71 – 1.61 (m, 4H), 1.35 (s, 3H), 1.23 (s, 3H), 1.20 (s, 3H), 1.18 (s, 3H), 1.17 (s, 3H); ^{13}C NMR (125 MHz, CD_2Cl_2): 165.4 (C), 132.8 (CH), 131.4 (CH), 130.0 (C), 128.2 (C), 85.7 (CH),

81.3 (C), 77.3 (CH), 75.0 (C), 73.5 (C), 38.8 (CH_2), 32.2 (CH_2), 29.9 (CH_3), 28.7 (CH_3), 27.8 (CH_3), 27.0 (CH_2), 24.1 (CH_3), 21.9 (CH_2), 21.5 (CH_3). ***trans,syn*-(AA)-23.** R_f (pentane/Et_2O 6:1): 0.55; ^{1}H NMR (400 MHz, CD_2Cl_2): 7.88 – 7.85 (m, 2H), 7.62 – 7.59 (m, 2H), 5.13 (dd, $^3J_{H-H}$ = 10.8, 1.2 Hz, 1H), 3.36 (dd, $^3J_{H-H}$ = 11.6, 4.5 Hz, 1H), 2.02 – 1.92 (m, 1H), 1.79 – 1.55 (m, 7H), 1.25 (s, 3H), 1.24 (s, 3H), 1.22 (s, 6H), 1.13 (s, 3H); ^{13}C NMR (100 MHz, CD_2Cl_2): 165.3 (C), 132.1 (CH), 131.4 (CH), 129.9 (C), 128.3 (C), 80.9 (CH), 76.3 (C), 76.3 (C), 73.7 (CH), 71.4 (C), 42.6 (CH_2), 37.8 (CH_2), 33.4 (CH_3), 27.8 (CH_3), 26.8 (CH_2), 25.2 (CH_2), 25.0 (CH_3), 23.3 (CH_3), 20.6 (CH_3).

Compounds *trans*-(BA)-3, and *trans*-(AA)-5. The above obtained compounds *trans,anti*-(BA)-**21**, *trans,syn*-(BA)-**21** and *trans,syn*-(AA)-**23** were dissolved in MeOH/CH_2Cl_2 (1:1, 25 mM) in separated vials, then corresponding amounts of K_2CO_3 (5 equiv) were added to each vials and the reactions were stirred at rt. The mixture was stirred at rt and stopped after 48 h when the complete consumption of the starting material was observed. The crude mixture was purified by silica gel column chromatography directly (pentane/Et_2O 19:1 to 2:1) to give *trans,anti*-(BA)-**3** (3.26 mg, 76%), *trans,syn*-(BA)-**3** (3.95 mg, 74%) and *trans,syn*-(AA)-**5** (4.5 mg, 79%) as light yellow oils. ***trans,anti*-(BA)-3.** R_f (pentane/Et_2O 1:1): 0.4; ^{1}H NMR (500 MHz, CD_2Cl_2): 3.89 (t, $^3J_{H-H}$ = 7.4 Hz, 1H), 3.36 (dd, $^3J_{H-H}$ = 5.8, 3.0 Hz, 1H), 1.97 – 1.78 (m, 4H), 1.73 – 1.55 (m, 4H), 1.35 – 1.30 (m, 1H), 1.23 (s, 3H), 1.22 (s, 3H), 1.19 (s, 3H), 1.13 (s, 3H), 1.10 (s, 3H); ^{13}C NMR (125 MHz, CD_2Cl_2): 85.0 (CH), 81.3 (C), 74.8 (C), 74.7 (C), 71.4 (CH), 38.8 (CH_2), 28.6 (CH_3), 28.3 (CH_3), 27.8 (CH_3), 27.0 (CH_2), 26.4 (CH_2), 26.2 (CH_2), 24.1 (CH_3), 24.0 (CH_3). ***trans,syn*-(BA)-3.** R_f (pentane/Et_2O 1:1): 0.4; ^{1}H NMR (500 MHz, CD_2Cl_2): 3.67 (t, $^3J_{H-H}$ = 7.1 Hz, 1H), 3.34 (dd, $^3J_{H-H}$ = 10.2, 5.2 Hz, 1H), 1.92 – 1.83 (m, 2H), 1.76 – 1.67

(m, 2H), 1.66 – 1.58 (m, 3H), 1.55 – 1.49 (m, 1H), 1.46 (brs, 1H), 1.21 (s, 3H), 1.18 (s, 3H), 1.16 (s, 6H), 1.13 (s, 3H); ^{13}C NMR (125 MHz, CD_2Cl_2): 86.4 (CH), 81.1 (C), 75.3 (CH), 75.0 (C), 74.6 (C), 38.7 (CH_2), 33.2 (CH_2), 30.0 (CH_3), 28.7 (CH_3), 27.8 (CH_3), 27.0 (CH_2), 25.3 (CH_2), 22.4 (CH_3), 21.1 (CH_3). ***trans,syn*-(AA)-5.** R_f (pentane/Et_2O 1:1): 0.4; ^{1}H NMR (500 MHz, CD_2Cl_2): 3.73 (d, $^3J_{H-H}$ = 9.8 Hz, 1H), 3.19 (dd, $^3J_{H-H}$ = 11.6, 4.5 Hz, 1H), 1.92 – 1.80 (m, 1H), 1.73 – 1.57 (m, 3H), 1.54 – 1.42 (m, 4H), 1.25 (s, 3H), 1.21 (s, 3H), 1.17 (d, $^4J_{H-H}$ = 1.2 Hz, 3H), 1.10 (s, 3H), 1.04 (s, 3H); ^{13}C NMR (125 MHz, CD_2Cl_2): 79.2 (CH), 77.4 (C), 76.4 (C), 73.2 (CH), 71.3 (C), 44.2 (CH_2), 37.8 (CH_2), 33.4 (CH_3), 30.3 (CH_3), 27.3 (CH_3), 25.3 (CH_2), 25.2 (CH_2), 22.0 (CH_3), 20.6 (CH_3).

3.3. Identification of products with acyclic and rearrangement motifs

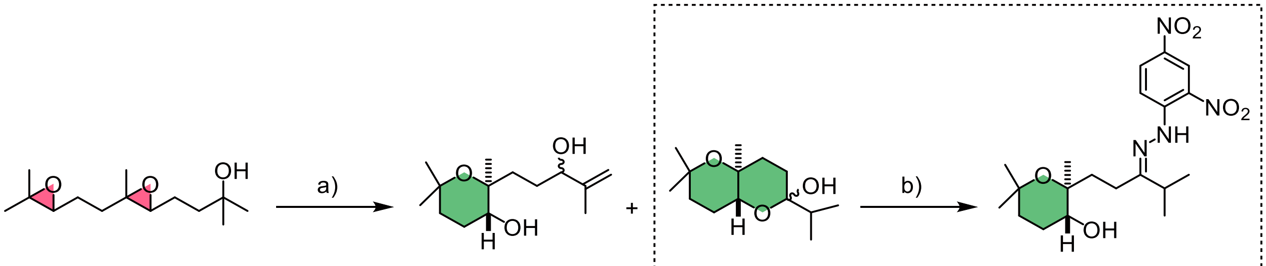

Scheme S5 (a) **8**, $CHCl_3$, rt, 7 days; (b) 2,4-dinitrophenylhydrazine, AcOH, EtOH, reflux, 12 h.

Compounds *trans*-(A-HM)-6, 7 and *trans*-(A-HM)-24. To a solution of *trans*-**1** (0.10 g, 0.41 mmol) in $CHCl_3$ (12.4 mL) was added capsule monomer **11** (274 mg, 0.250 mmol, capsule catalyst **8** was self-assembled from six molecules of **11** in the solution) at rt. The mixture was stirred at rt and stopped after 7 days when the complete consumption of the starting material was observed. The crude mixture was purified by silica gel column chromatography directly (pentane/Et_2O 19:1 to 1:2) to give *trans*-(A-HM)-**6** and **7** as light yellow oils. The isolated *trans*-(A-HM)-**6** was dissolved in EtOH (6.0 mL), then 2,4-dinitrophenylhydrazine (818 mg, 4.13 mmol) and AcOH (236 µL, 4.13 mmol) were added to the solution successively. The reaction mixture was refluxed for 12 h, the crude mixture was purified by silica gel column chromatography (pentane/Et_2O 19:1 to 2:3) to give *trans*-(A-HM)-**24** as yellow oil. ***trans*-(A-HM)-6.** R_f (pentane/Et_2O 1:1): 0.35; ^{1}H NMR (500 MHz, CD_2Cl_2): 3.44 – 3.41 (m, 1H), 2.24 (brs, 1H), 2.07 – 1.97 (m, 2H), 1.77 – 1.73 (m, 2H), 1.66 – 1.63 (m, 1H), 1.55 – 1.44 (m, 4H), 1.39 (s, 3H), 1.20 – 1.18 (m, 6H), 0.98 (d, $^3J_{H-H}$ = 7.0 Hz, 3H), 0.97(6) (d, $^3J_{H-H}$ = 7.0 Hz, 3H); ^{13}C NMR (125 MHz, CD_2Cl_2): 112.4 (C), 86.5 (C), 85.9 (CH), 69.5 (C), 41.9 (CH_2), 33.6 (CH_2),

31.7 (CH), 30.2 (CH_3), 29.7 (CH_2), 29.0 (CH_3), 26.5 (CH_2), 17.9 (CH_3), 17.8 (CH_3), 17.5 (CH_3). **7** (d.r. 59:41). R_f (pentane/Et_2O 1:1): 0.2; ^1H NMR (400 MHz, CD_2Cl_2): 5.00 – 4.96 (m, 1H), 4.79 – 4.77 (m, 1H), 4.38 – 4.31 (m, 1H), 3.50 – 3.47 (m, 1H), 2.15 – 2.02 (m, 2H), 1.84 – 1.50 (m, 8H), 1.42 – 1.32 (m, 1H), 1.20 (s, 6H), 1.18 – 1.16 (m, 3H); ^{13}C NMR (100 MHz, CD_2Cl_2): 146.7 (C), 146.1 (C), 110.1 (CH_2), 86.7 (C), 86.5 (C), 84.3 (CH), 81.1 (CH), 77.7(0) (CH), 77.6(8) (CH), 70.6(2) (C), 70.5(7) (C), 41.5 (CH_2), 41.4 (CH_2), 32.1 (CH_2), 32.0 (CH_2), 31.9 (CH_2), 31.8 (CH_2), 30.0 (CH_3), 29.3(3) (CH_3), 29.3(0) (CH_3), 26.6 (CH_2), 24.1 (CH_3), 22.9 (CH_3), 18.4 (CH_3), 18.0 (CH_3). ***trans*-(A-HM)-24.** R_f (Et_2O/pentane 2:1): 0.4; ^1H NMR (400 MHz, CD_2Cl_2): 11.16 (s, 1H), 9.07 (d, $^4J_{H\text{-}H}$ = 2.6 Hz, 1H), 8.27 (dd, $^3J_{H\text{-}H}$ = 9.7 Hz, $^4J_{H\text{-}H}$ = 2.7 Hz, 1H), 7.99 (d, $^3J_{H\text{-}H}$ = 9.7 Hz, 1H), 3.45 (d, $^3J_{H\text{-}H}$ = 10.3 Hz, 1H), 2.73 – 2.65 (m, 2H), 2.53 – 2.45 (m, 1H), 1.93 – 1.80 (m, 1H), 1.72 – 1.56 (m, 4H), 1.48 – 1.39 (m, 1H), 1.25 – 1.21 (m, 15H); ^{13}C NMR (100 MHz, CD_2Cl_2): 167.1 (C), 146.0 (C), 137.9 (C), 130.2 (CH), 129.4 (C), 123.8 (CH), 117.0 (CH), 79.6 (CH), 74.3 (C), 71.3 (C), 41.2 (CH_2), 36.9 (CH), 31.2 (CH_2), 30.6 (CH_3), 29.0 (CH_3), 26.6 (CH_2), 23.4 (CH_2), 22.9 (CH_3), 20.4 (CH_3), 20.3 (CH_3).

4. Catalysis

4.1. Catalysis with *cis* diepoxide substrates

Table S1 Catalyst comparison on the diepoxide substrates *cis*-**1**, *cis*,*syn*-**1** and *cis*,*anti*-**1**[a]

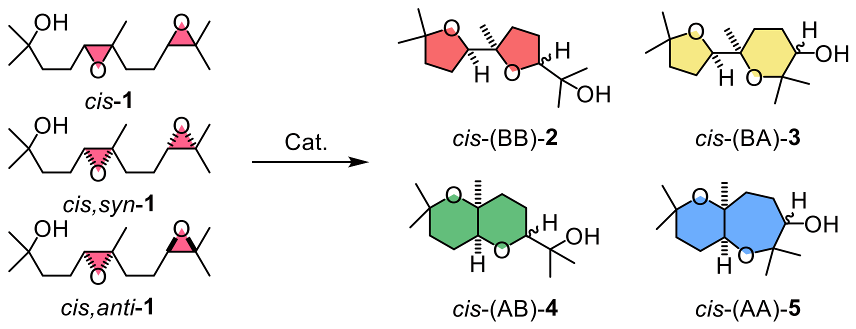

Entry	Sub[b]	Cat (mol%)[c]	*T* (°C)[d]	*t*[e]	η_t (%)[f]	BB:BA:AB:AA[g]
1	*cis*-**1**	AcOH (500)	40	2 d	>95	88:11:1:0
2	*cis*,*syn*-**1**	AcOH (500)	40	2 d	>95	83:16:1:0
3	*cis*,*anti*-**1**	AcOH (500)	40	2 d	>95	94:5:1:0[h]
4	*cis*-**1**	**8** (10)	30	7 d	>95	51:43:4:2
5	*cis*,*syn*-**1**	**8** (10)	30	7 d	>95	41:57:0:2
6	*cis*,*anti*-**1**	**8** (10)	30	7 d	>95	62:27:9:2[h]
7	*cis*-**1**	**9** (2.5)	rt	1 h	>95	2:56:31:11
8	*cis*,*syn*-**1**	**9** (2.5)	rt	1 h	>95	2:79:0:19
9	*cis*,*anti*-**1**	**9** (2.5)	rt	1 h	>95	0:29:67:2[h]
10	*cis*,*anti*-**1**	**9** (2.5)	rt	1 h	>95	3:39:58:0

Table S1 (continued) Catalyst comparison on the diepoxide substrates *cis*-**1**, *cis,syn*-**1** and *cis,anti*-**1**[a]

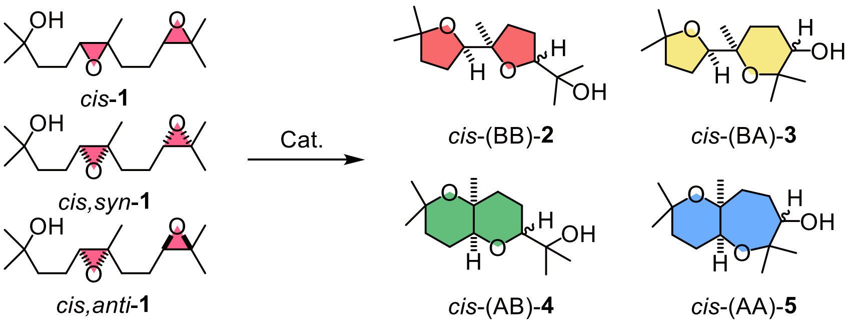

Entry	Sub[b]	Cat (mol%)[c]	T (°C)[d]	t[e]	η_t (%)[f]	BB:BA:AB:AA[g]
11	*cis*-**1**	**10** (10)	rt	8 d	>95	79:17:3:1
12	*cis,syn*-**1**	**10** (10)	rt	8 d	>95	86:14:0:0
13	*cis,anti*-**1**	**10** (10)	rt	8 d	>95	71:21:7:1[h]

[a]Reaction conditions and data are indicated in the table. [b]Substrates. [c]Catalysts (Figure S1). In parethesis, catalyst concentration in mol% relative to concentration of di-epoxide substrates. [d]Reaction temperature; rt: room temperature. [e]Reaction time. [f]Substrate conversion, in percent, from ^{1}H NMR spectra of product mixtures. [g]Selectivities were obtained from GC-FID analysis and the corresponding substrate was indicated in parenthesis. [h]Results for *cis,anti* isomer **1** are calculated (from data for the other diastereomer and the mixture of diastereomers in the respective series)

4.1.1. Brønsted-acid catalyst AcOH

To a solution of *cis* diepoxide substrates (125 mM) in CD_2Cl_2 was added AcOH (500 mol%), then the mixture was stirred at 40 °C. The reaction was monitored by ^{1}H NMR spectroscopy and stopped after 2 days when full consumption of the starting material was observed. An aliquot (50 µL) of the resulting solution was filtered over a filter tip using diethyl ether as the eluent and the resulting sample was subjected to GC-FID analysis (Conditions are described in the Materials and methods section).

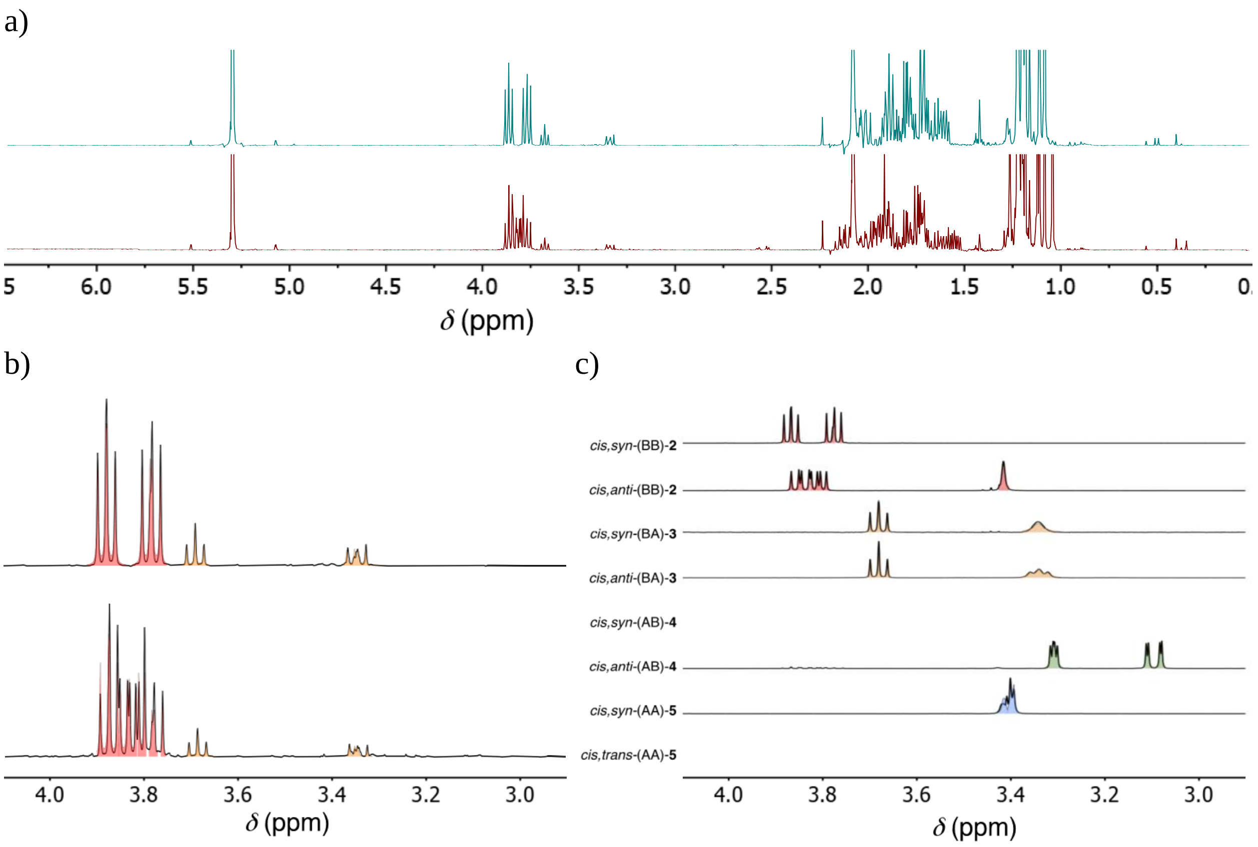

Figure S4. Crude ^{1}H NMR spectra analysis of the epoxide-opening cascade cyclization of *cis*-**1** (bottom) and *cis*,*syn*-**1** (top) catalyzed by AcOH. a) Full ^{1}H NMR spectra. b) Zoomed ^{1}H NMR spectra. c) Decoded NMR fingerprint region for products of *cis* diepoxide substrates.

a)

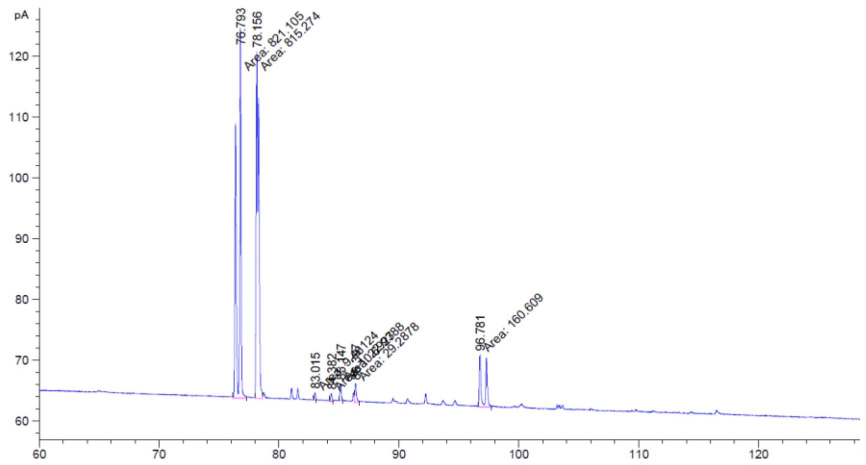

Product	Ret. time (min)	Area (pA*s)	Area (%)
BB	76.793	821.1	43.9
BB	78.156	815.3	43.6
AB	83.015	9.5	0.5
AB	84.382	10.7	0.6
BA	85.147	22.9	1.2
BA	86.397	29.3	1.6
BA	96.781	160.6	8.6

b)

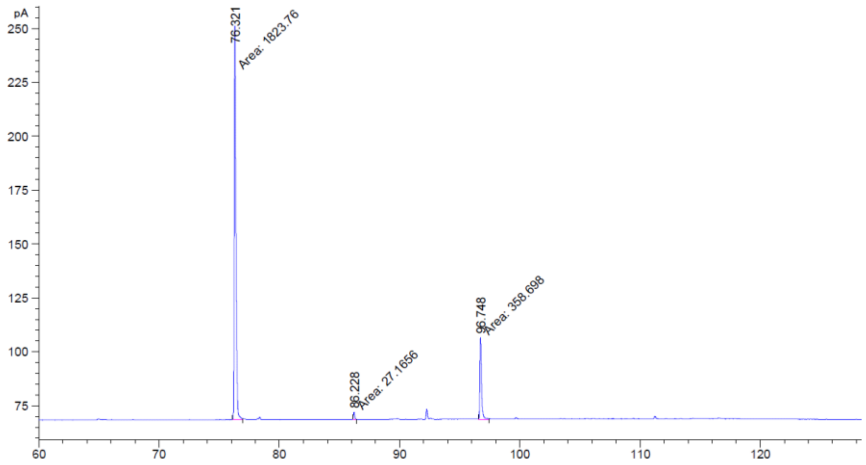

Product	Ret. time (min)	Area (pA*s)	Area (%)
BB	76.321	1823.8	82.5
BA	86.228	27.2	1.2
BA	96.748	358.7	16.2

Figure S5. GC analysis of epoxides a) *cis*-**1** and b) *cis*,*syn*-**1** opening catalyzed by AcOH.

4.1.2. π-Basic capsule catalyst 8

To a solution of *cis* diepoxide substrates (33.3 mM) in $CHCl_3$ (Pre-treated as described in the Materials and methods section) was added capsule monomer **11** (60 mol%, capsule catalyst **8** was self-assembled from six molecules of **11** in the solution). The mixture was stirred at rt and stopped after 7 days when the complete consumption of the starting material was observed. An aliquot (100 µL) of the resulting solution was filtered over a filter tip using diethyl ether as the eluent and the resulting sample was subjected to GC-FID analysis (Conditions are described in the Materials and methods section). Then the resulting GC sample was subjected to ^{1}H NMR spectroscopy analysis.

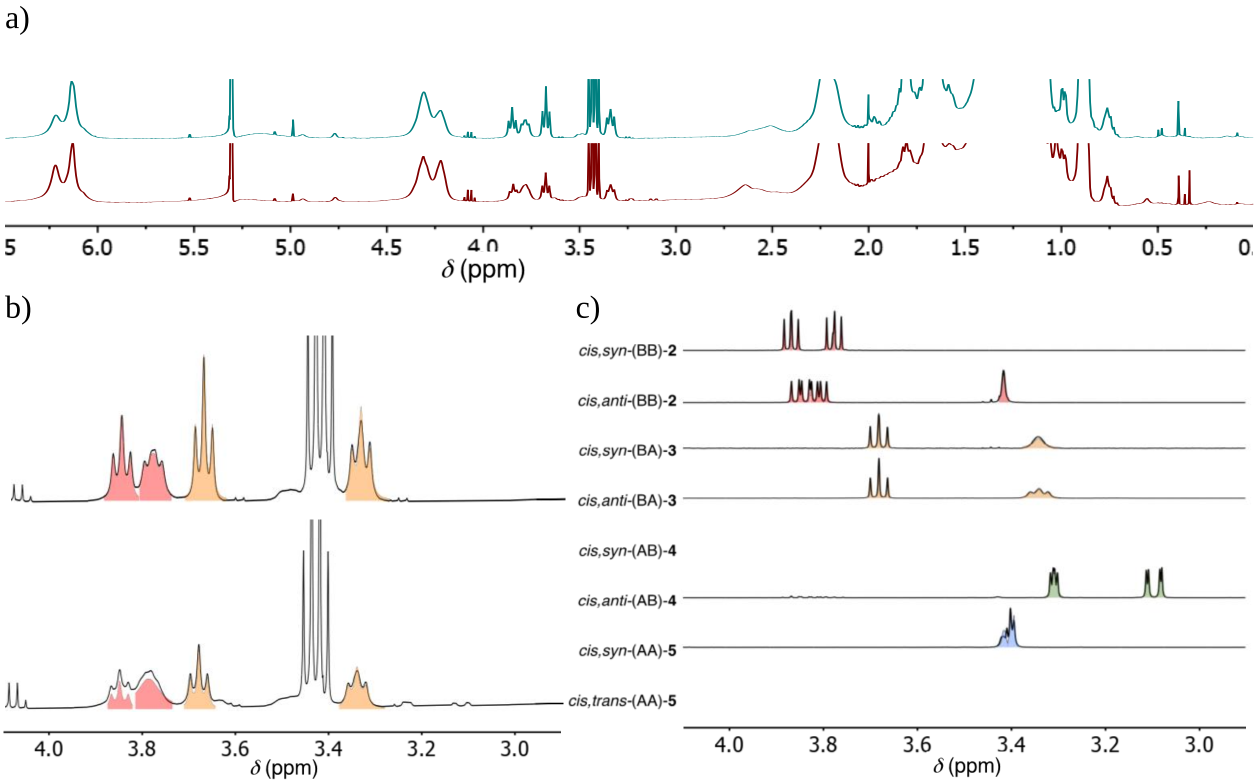

Figure S6. Crude ^{1}H NMR spectra analysis of the epoxide-opening cascade cyclization of *cis*-**1** (bottom) and *cis,syn*-**1** (top) catalyzed by capsule catalyst **8**. a) Full ^{1}H NMR spectra. b) Zoomed ^{1}H NMR spectra. c) Decoded NMR fingerprint region for products of *cis* diepoxide substrates.

a)

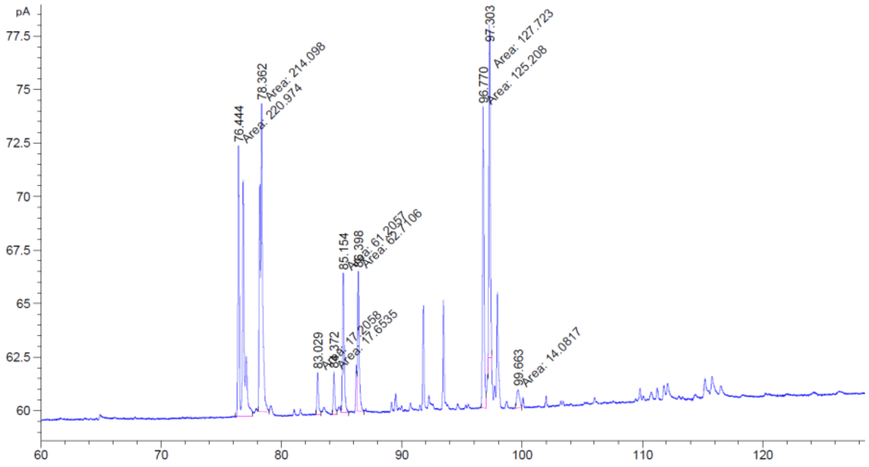

Product	Ret. time (min)	Area (pA*s)	Area (%)
BB	76.444	221.0	25.7
BB	78.362	214.1	24.9
AB	83.029	17.2	2.0
AB	84.372	17.7	2.1
BA	85.154	61.2	7.1
BA	86.398	62.7	7.3
BA	96.770	125.2	14.5
BA	97.303	127.7	14.8
AA	99.663	14.1	1.6

b)

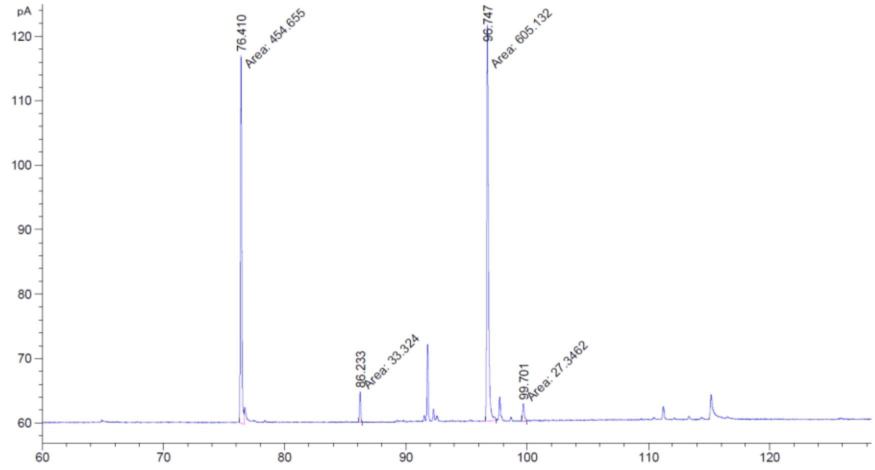

Product	Ret. time (min)	Area (pA*s)	Area (%)
BB	76.410	454.7	40.6
BA	86.233	33.3	3.0
BA	96.747	605.1	54.0
AA	99.701	27.3	2.4

Figure S7. GC analysis of epoxides a) *cis*-**1** and b) *cis*,*syn*-**1** opening catalyzed by capsule catalyst **8**.

4.1.3. Pnictogen-bonding catalyst 9

To a solution of *cis* diepoxide substrates (125 mM) in CD_2Cl_2 was added catalyst **9** (2.5 mol%), then the mixture was stirred at rt. The reaction was monitored by ^{1}H NMR spectroscopy and stopped after 1 h when the complete consumption of the starting material was observed. An aliquot (50 µL) of the resulting solution was filtered over a filter tip using diethyl ether as the eluent and the resulting sample was subjected to GC-FID analysis (Conditions are described in the Materials and methods section).

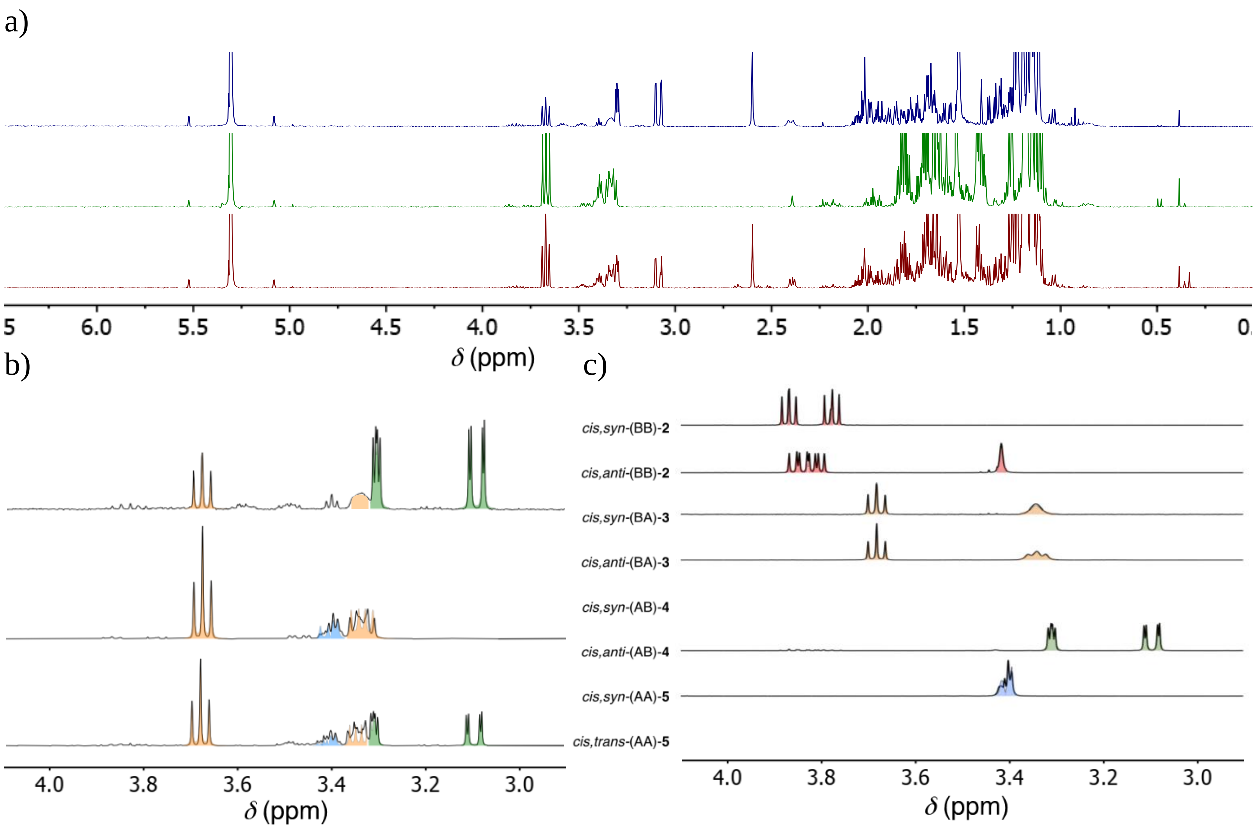

Figure S8. Crude ^{1}H NMR spectra analysis of the epoxide-opening cascade cyclization of *cis*-**1** (bottom), *cis*,*syn*-**1** (middle) and *cis*,*anti*-**1** (top) catalyzed by pnictogen-bonding catalyst **9**. a) Full ^{1}H NMR spectra. b) Zoomed ^{1}H NMR spectra. c) Decoded NMR fingerprint region for products of *cis* diepoxide substrates.

a)

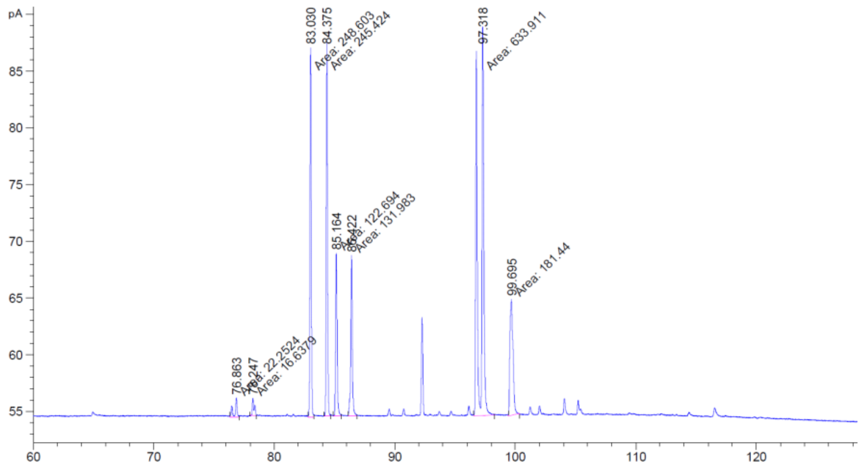

Product	Ret. time (min)	Area (pA*s)	Area (%)
BB	76.863	22.3	1.4
BB	78.247	16.6	1.0
AB	83.030	248.6	15.5
AB	84.375	245.4	15.3
BA	85.164	122.7	7.7
BA	86.422	132.0	8.2
BA	97.318	633.9	39.5
AA	99.695	181.4	11.3

b)

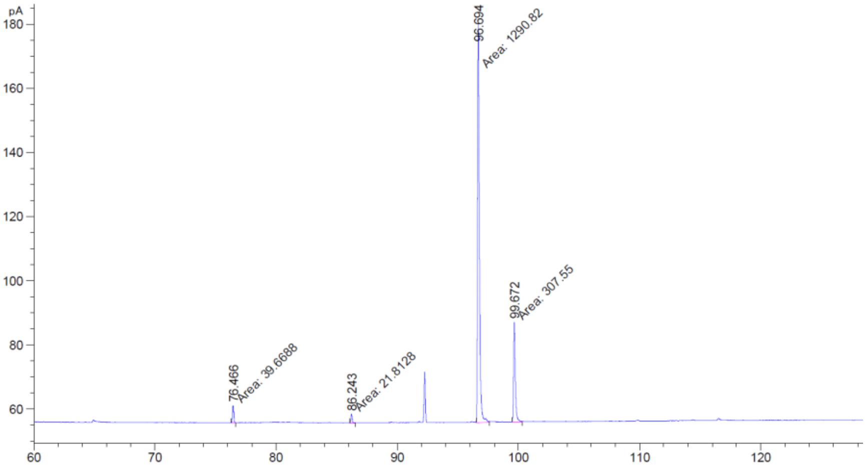

Product	Ret. time (min)	Area (pA*s)	Area (%)
BB	76.466	39.7	2.4
BA	86.243	21.8	1.3
BA	96.694	1290.8	77.8
AA	99.672	307.6	18.5

Figure S9. GC analysis of epoxides a) *cis*-**1** and b) *cis*,*syn*-**1** opening catalyzed by pnictogen-bonding catalyst **9**.

c)

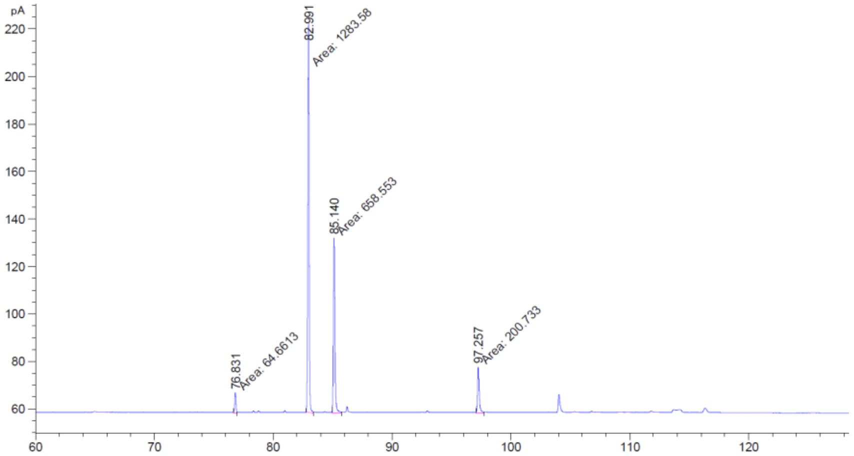

Product	Ret. time (min)	Area (pA*s)	Area (%)
BB	76.831	64.7	2.9
AB	82.991	1283.6	58.1
BA	85.140	658.6	29.8
BA	97.257	200.7	9.1

Figure S9 (continued). GC analysis of epoxide c) *cis*,*anti*-**1** opening catalyzed by pnictogen-bonding catalyst **9**.

4.1.4. π-Acidic catalyst 10

To a solution of *cis* diepoxide substrates (1.5 M) in CD_2Cl_2 was added catalyst **10** (10 mol%), then the mixture was stirred at rt. The reaction was monitored by ^{1}H NMR spectroscopy and stopped after 8 days when the complete consumption of the starting material was observed. An aliquot (50 µL) of the resulting solution was filtered over a filter tip using diethyl ether as the eluent and the resulting sample was subjected to GC-FID analysis (Conditions are described in the Materials and methods section).

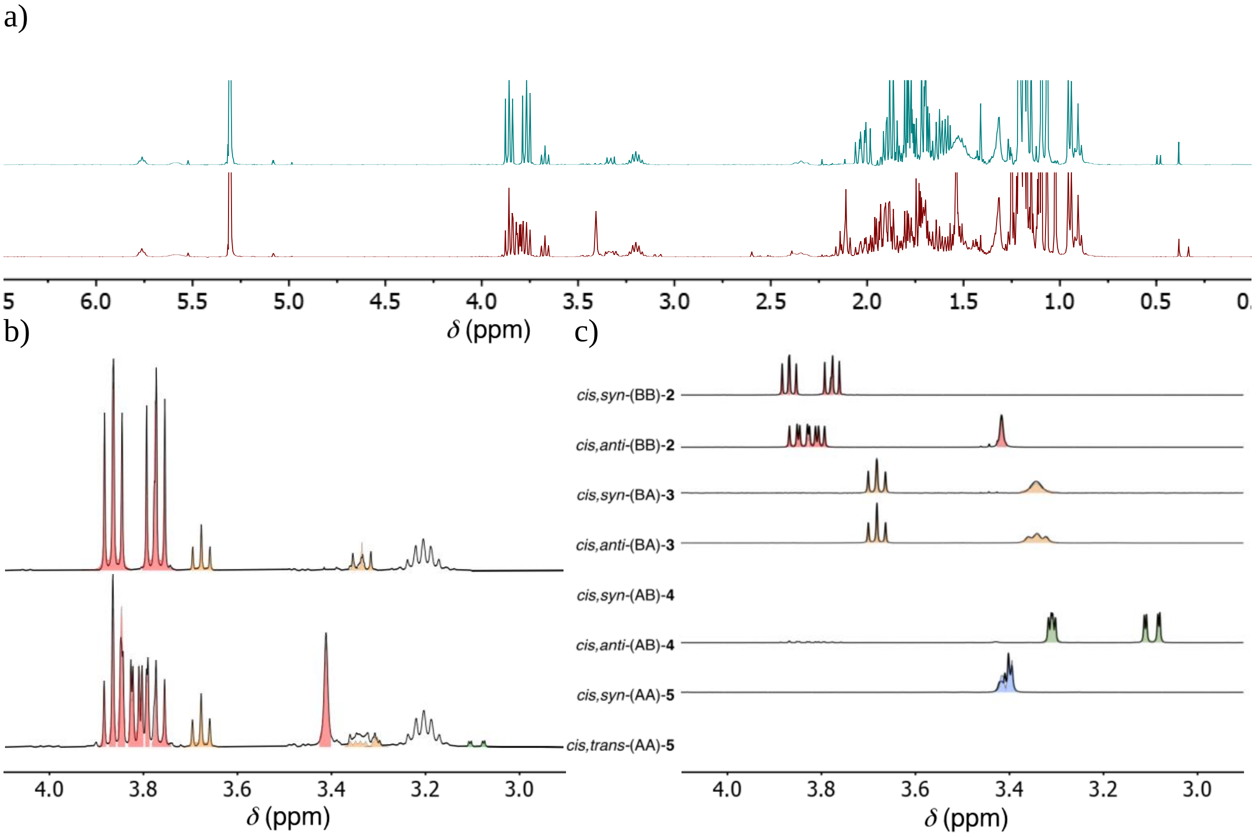

Figure S10. Crude ^{1}H NMR spectra analysis of the epoxide-opening cascade cyclization of *cis*-**1** (bottom) and *cis,syn*-**1** (top) catalyzed by π-acidic catalyst **10**. a) Full ^{1}H NMR spectra. b) Zoomed ^{1}H NMR spectra. c) Decoded NMR fingerprint region for products of *cis* diepoxide substrates.

a)

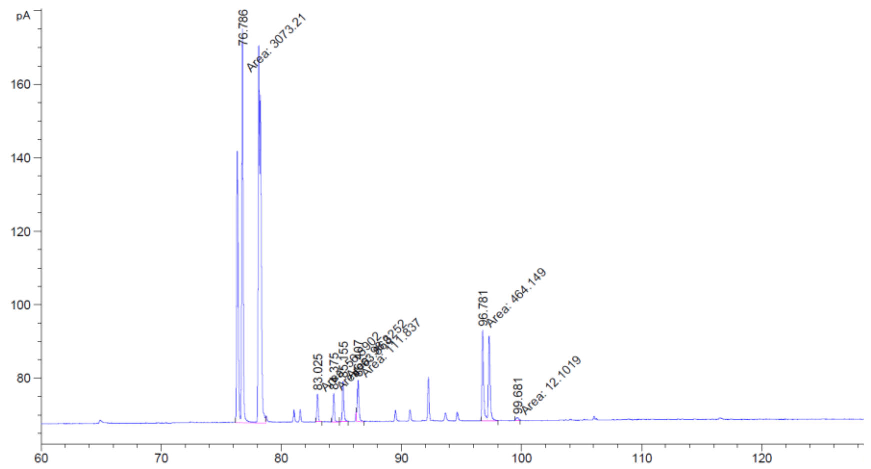

Product	Ret. time (min)	Area (pA*s)	Area (%)
BB	76.786	3073.2	79.2
AB	83.025	56.6	1.5
AB	84.375	63.9	1.6
BA	85.155	98.3	2.5
BA	86.407	111.8	2.9
BA	96.781	464.1	12.0
AA	99.681	12.1	0.3

b)

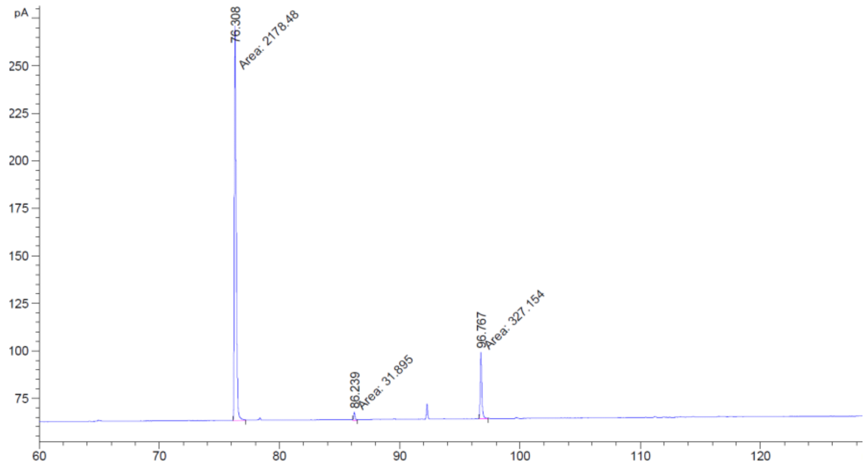

Product	Ret. time (min)	Area (pA*s)	Area (%)
BB	76.308	2178.5	85.9
BA	86.239	31.9	1.3
BA	96.767	327.2	12.9

Figure S11. GC analysis of epoxides a) *cis*-**1** and b) *cis*,*syn*-**1** opening catalyzed by anion-π catalyst **10**.

4.2. Catalysis with *trans* diepoxide substrates

Table S2 Catalyst comparison on the diepoxide substrates *trans*-**1**, *trans,syn*-**1** and *trans,anti*-**1**[a]

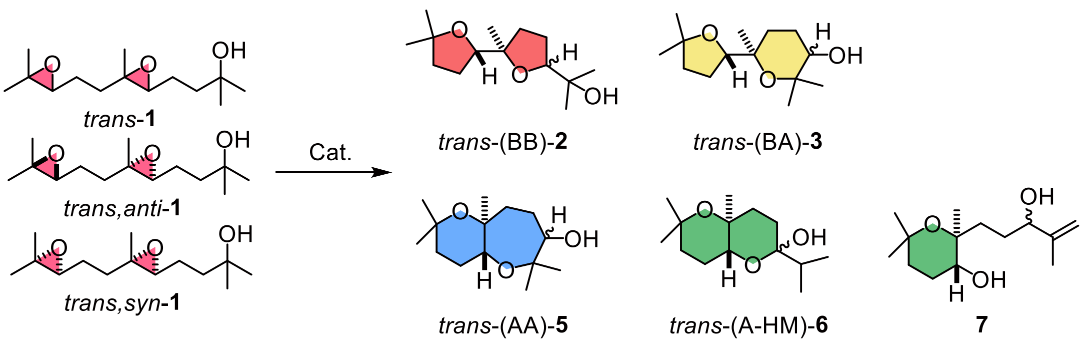

Entry	Sub[b]	Cat (mol%)[c]	*T* (°C)[d]	*t*[e]	η_t (%)[f]	BB:BA:(**6**+**7**):AA[g]
1	*trans*-**1**	AcOH (500)	40	2 d	>95	89:8:0:3
2	*trans,anti*-**1**	AcOH (500)	40	2 d	>95	92:4:0:4
3	*trans,syn*-**1**	AcOH (500)	40	2 d	>95	86:12:0:2[h]
4	*trans*-**1**	**8** (10)	30	7 d	>95	28:6:47:18
5	*trans,anti*-**1**	**8** (10)	30	7 d	>95	20:4:48:28
6	*trans,syn*-**1**	**8** (10)	30	7 d	>95	37:8:47:8[h]
7	*trans*-**1**	**9** (2.5)	rt	1 h	>95	9:11:0:80
8	*trans,anti*-**1**	**9** (2.5)	rt	1 h	>95	10:13:0:77
9	*trans,syn*-**1**	**9** (2.5)	rt	1 h	>95	8:9:0:83[h]

Table S2 (continued) Catalyst comparison on the diepoxide substrates *trans*-**1**, *trans,syn*-**1** and *trans,anti*-**1**[a]

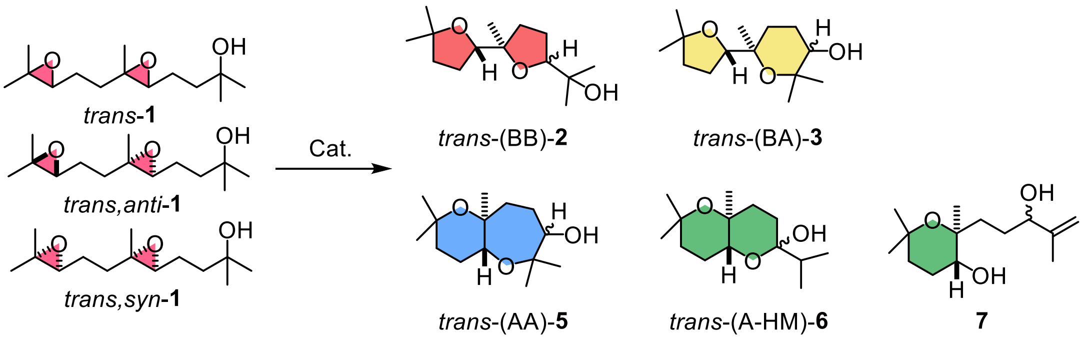

Entry	Sub[b]	Cat (mol%)[c]	T (°C)[d]	t[e]	η_t (%)[f]	BB:BA:AB:AA[g]
10	*trans*-**1**	**10** (10)	rt	8 d	>95	80:13:0:7
11	*trans,anti*-**1**	**10** (10)	rt	8 d	>95	82:10:0:8
12	*trans,syn*-**1**	**10** (10)	rt	8 d	>95	78:16:0:6[h]

[a]Reaction conditions and data are indicated in the table. [b]Substrates. [c]Catalysts (Figure S1). In parethesis, catalyst concentration in mol% relative to concentration of di-epoxide substrates. [d]Reaction temperature; rt: room temperature. [e]Reaction time. [f]Substrate conversion, in percent, from ^{1}H NMR spectra of product mixtures. [g]Selectivities were obtained from GC-FID analysis and the corresponding substrate was indicated in parenthesis. [h]Results for *trans,syn* isomer **1** are calculated (from data for the other diastereomer and the mixture of diastereomers in the respective series).

4.2.1. Brønsted-acid catalyst AcOH

To a solution of *trans* diepoxide substrates (125 mM) in CD_2Cl_2 was added AcOH (500 mol%), then the mixture was stirred at 40 °C. The reaction was monitored by ^{1}H NMR spectroscopy and stopped after 2 days when the complete consumption of the starting material was observed. An aliquot (50 µL) of the resulting solution was filtered over a filter tip using diethyl ether as the eluent and the resulting sample was subjected to GC-FID analysis (Conditions are described in the Materials and methods section).

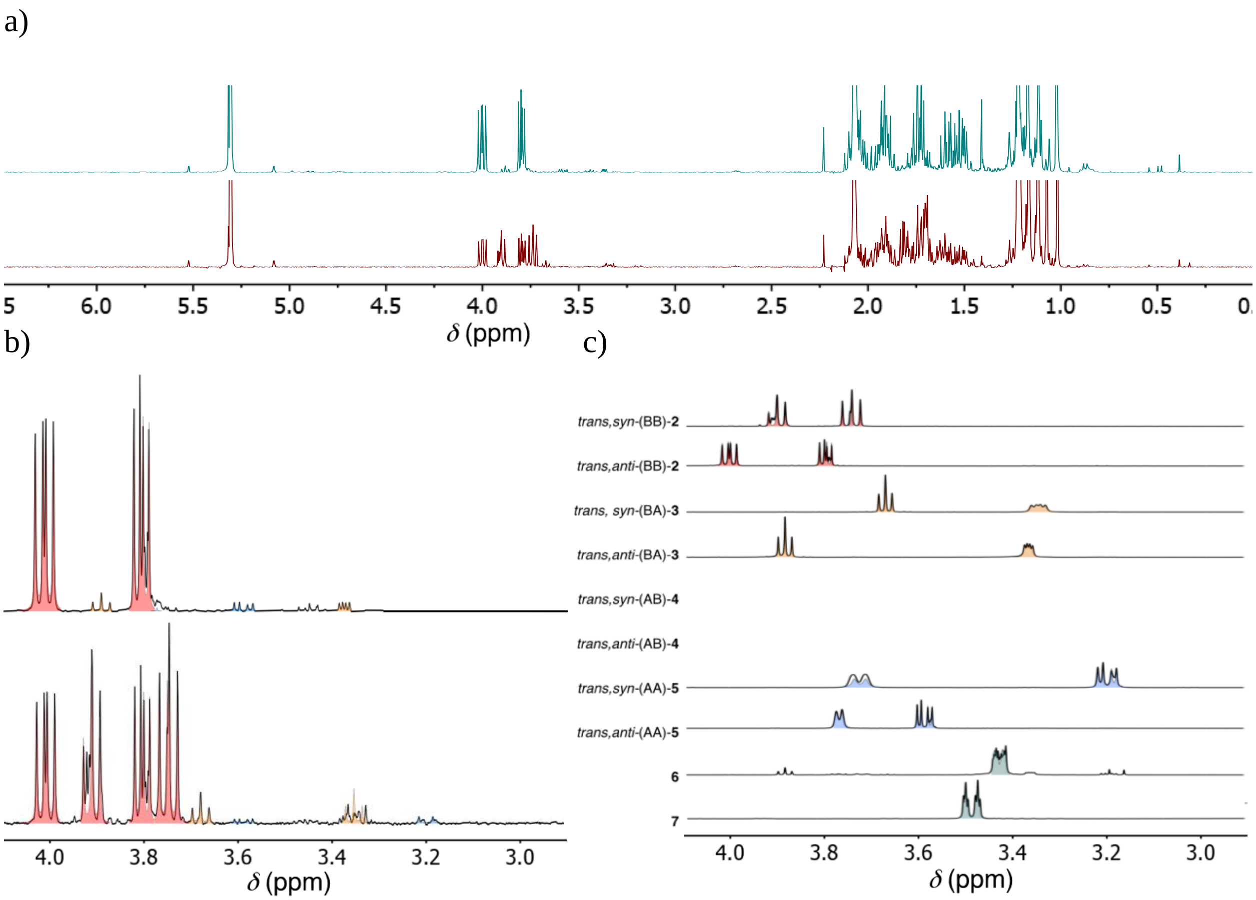

Figure S12. Crude ^{1}H NMR spectra analysis of the epoxide-opening cascade cyclization of *trans*-**1** (bottom) and *trans,anti*-**1** (top) catalyzed by AcOH. a) Full ^{1}H NMR spectra. b) Zoomed ^{1}H NMR spectra. c) Decoded NMR fingerprint region for products of *trans* diepoxide substrates.

a)

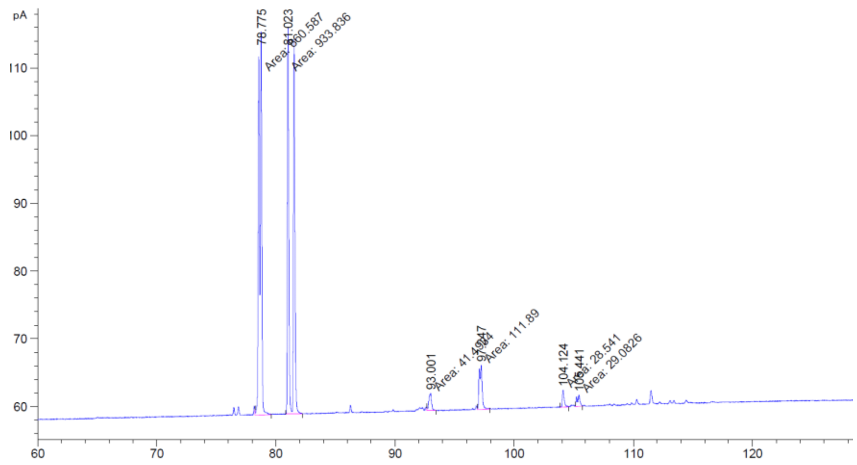

Product	Ret. time (min)	Area (pA*s)	Area (%)
BB	78.775	860.6	42.9
BB	81.023	933.8	46.6
BA	93.001	41.5	2.1
BA	97.247	111.9	5.6
AA	104.124	28.5	1.4
AA	105.441	29.1	1.5

b)

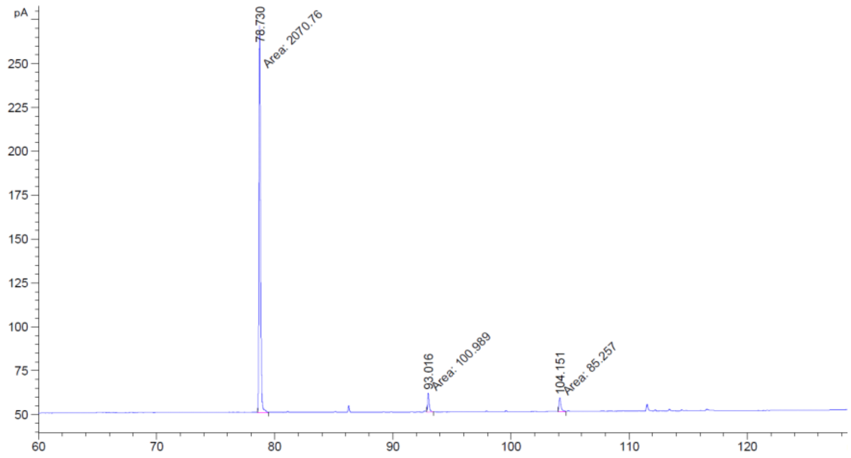

Product	Ret. time (min)	Area (pA*s)	Area (%)
BB	78.730	2070.8	91.7
BA	93.016	101.0	4.5
AA	104.151	85.3	3.8

Figure S13. GC analysis of epoxides a) *trans*-**1** and b) *trans*,*anti*-**1** opening catalyzed by AcOH.

4.2.2. π-Basic capsule catalyst 8

To a solution of *trans* diepoxide substrates (33.3 mM) in $CHCl_3$ (Pre-treated as described in the Materials and methods section) was added capsule monomer **11** (60 mol%, capsule catalyst **8** was self-assembled from six molecules of **11** in the solution), then the mixture was stirred at rt. The mixture was stirred at rt and stopped after 7 days when the complete consumption of the starting material was observed. An aliquot (100 µL) of the resulting solution was filtered over a filter tip using diethyl ether as the eluent and the resulting sample was subjected to GC-FID analysis (Conditions are described in the Materials and methods section). Another aliquot (500 µL) of the reaction mixture was chromatographed by a silica gel column using hexane/diethyl ether (9:1 to 1:1) as the eluent to remove capsule monomer **11** and the resulting sample was subjected to ^{1}H NMR spectroscopy analysis, GC analysis of the mixture showed the same products distribution as the crude.

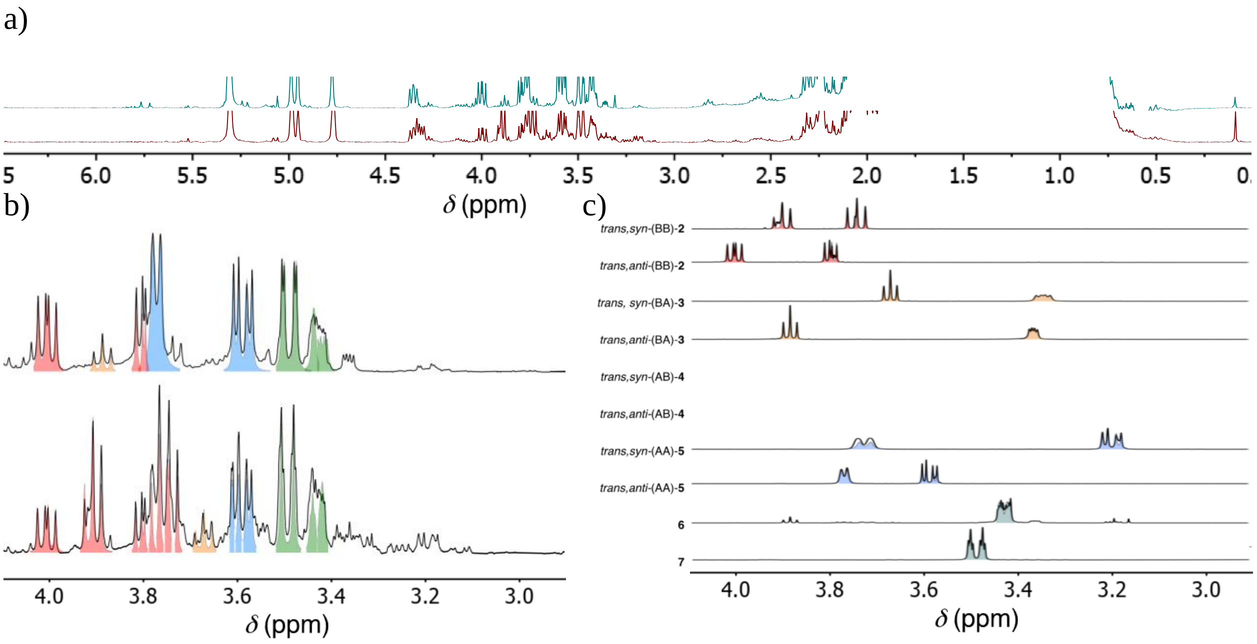

Figure S14. Crude ^{1}H NMR spectra analysis of the epoxide-opening cascade cyclization of *trans*-**1** (bottom) and *trans,anti*-**1** (top) catalyzed by capsule catalyst **8**. a) Full ^{1}H NMR spectra. b) Zoomed ^{1}H NMR spectra. c) Decoded NMR fingerprint region for products of *trans* diepoxide substrates.

a)

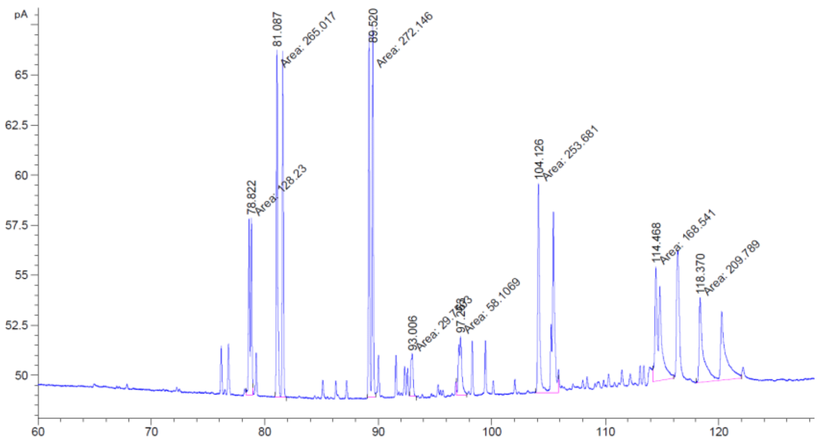

Product	Ret. time (min)	Area (pA*s)	Area (%)
BB	78.822	128.2	9.3
BB	81.087	265.0	19.1
**6**	89.520	272.1	19.6
BA	93.006	29.8	2.1
BA	97.263	58.1	4.2
AA	104.126	253.7	18.3
**7**	114.468	168.5	12.2
**7**	118.370	209.8	15.1

b)

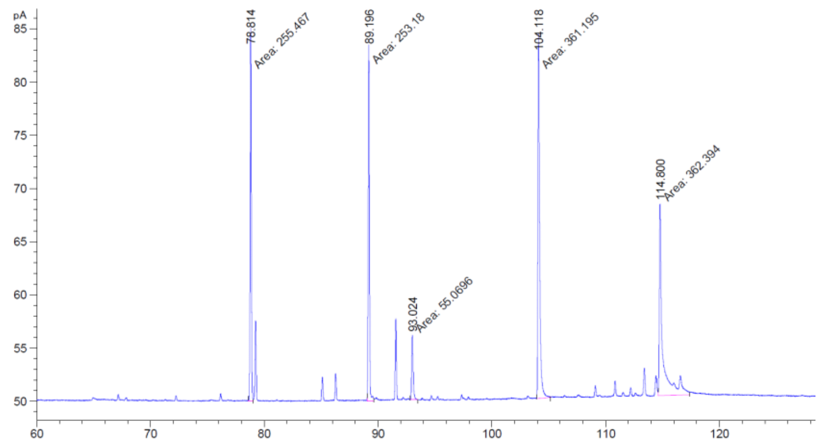

Product	Ret. time (min)	Area (pA*s)	Area (%)
BB	78.814	255.5	19.8
**6**	89.196	253.2	19.7
BA	93.024	55.1	4.3
AA	104.118	361.2	28.1
**7**	114.800	362.4	28.2

Figure S15. GC analysis of epoxides a) *trans*-**1** and b) *trans*,*anti*-**1** opening catalyzed by capsule catalyst **8**.

4.2.3. Pnictogen-bonding catalyst 9

To a solution of *trans* diepoxide substrates (125 mM) in CD_2Cl_2 was added catalyst **9** (2.5 mol%), then the mixture was stirred at rt. The reaction was monitored by ^{1}H NMR spectroscopy and stopped after 1 h when the complete consumption of the starting material was observed. An aliquot (50 µL) of the resulting solution was filtered over a filter tip using diethyl ether as the eluent and the resulting sample was subjected to GC-FID analysis (Conditions are described in the Materials and methods section).

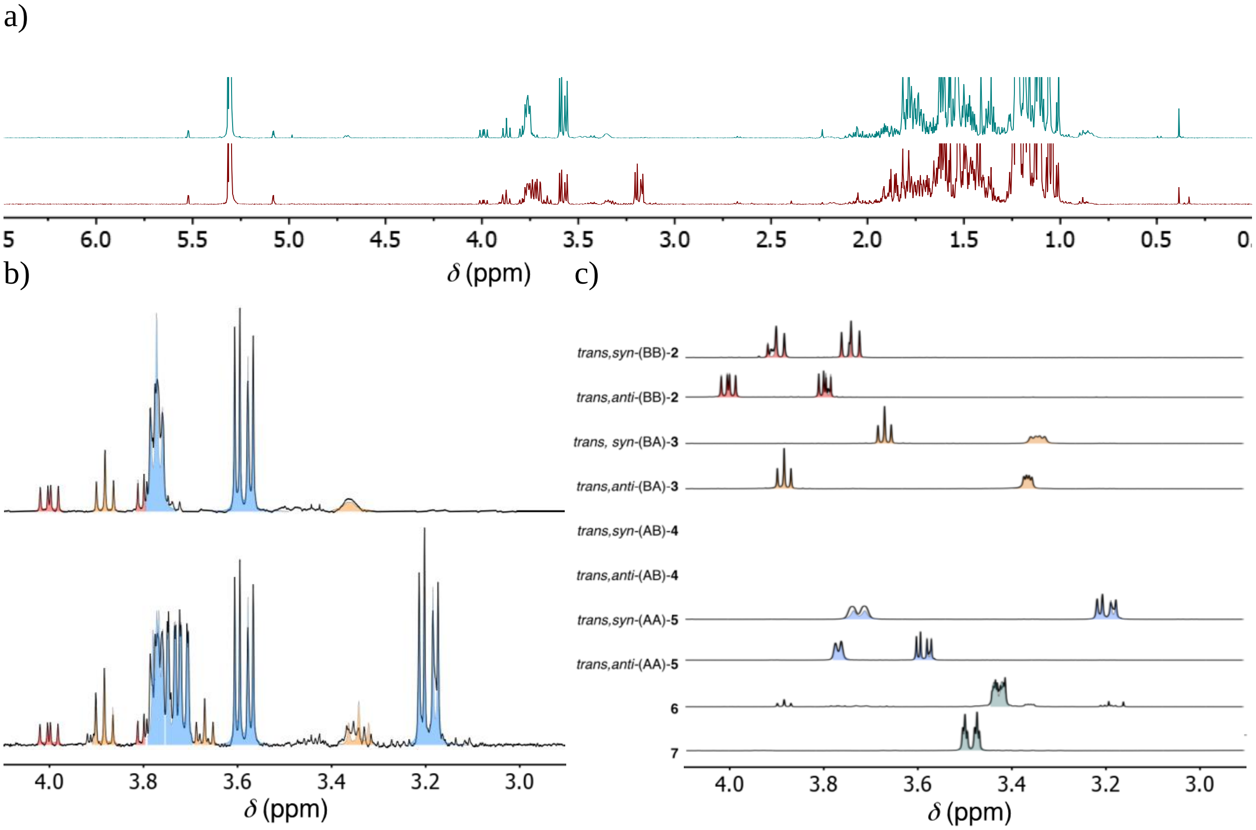

Figure S16. Crude ^{1}H NMR spectra analysis of the epoxide-opening cascade cyclization of *trans*-**1** (bottom) and *trans*,*anti*-**1** (top) catalyzed by pnictogen-bonding catalyst **9**. a) Full ^{1}H NMR spectra. b) Zoomed ^{1}H NMR spectra. c) Decoded NMR fingerprint region for products of *trans* diepoxide substrates.

a)

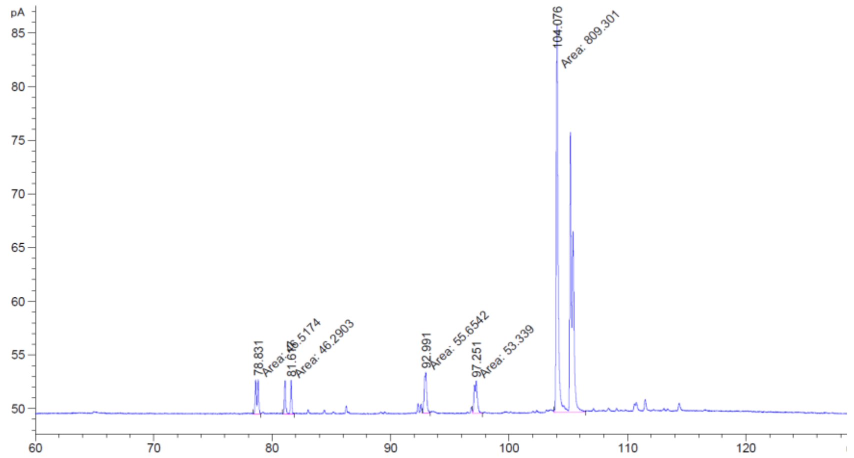

Product	Ret. time (min)	Area (pA*s)	Area (%)
BB	78.831	46.5	4.6
BB	81.617	46.3	4.6
BA	92.991	55.7	5.5
BA	97.251	53.3	5.3
AA	104.076	809.3	80.0

b)

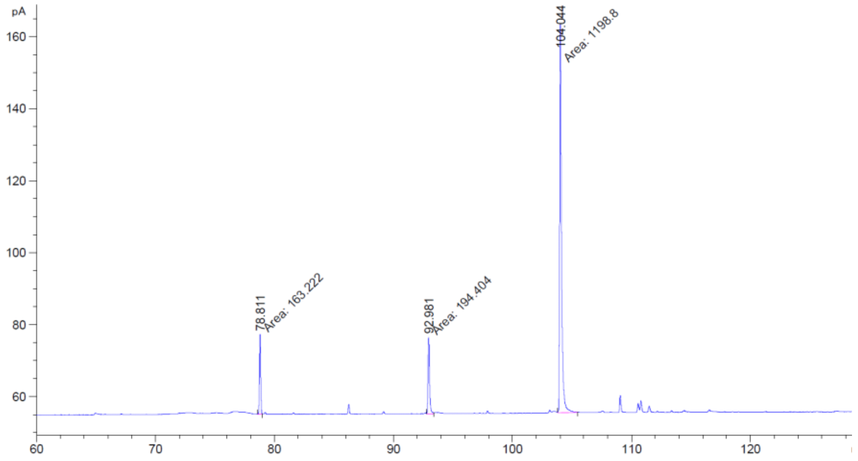

Peak	Ret. time (min)	Area (pA*s)	Area (%)
BB	78.8	163.2	10.5
BA	93.0	194.4	12.5
AA	104.0	1198.8	77.0

Figure S17. GC analysis of epoxides a) *trans*-**1** and b) *trans*,*anti*-**1** opening catalyzed by pnictogen-bonding catalyst **9**.

4.2.4. π-Acidic catalyst 10

To a solution of *trans* diepoxide substrates (1.5 M) in CD_2Cl_2 was added catalyst **10** (10 mol%), then the mixture was stirred at rt. The reaction was monitored by ^{1}H NMR spectroscopy and stopped after 8 days when the complete consumption of the starting material was observed. An aliquot (50 µL) of the resulting solution was filtered over a filter tip using diethyl ether as the eluent and the resulting sample was subjected to GC-FID analysis (Conditions are described in the Materials and methods section).

a)

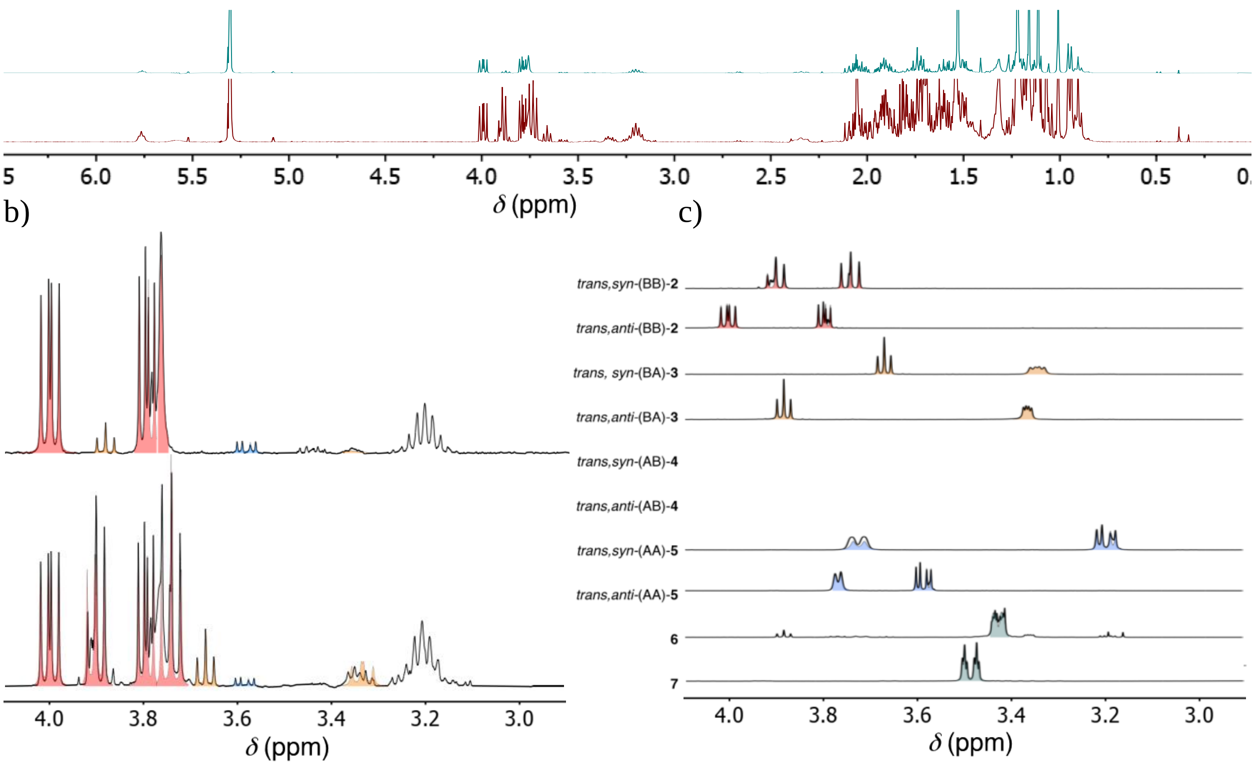

Figure S18. Crude ^{1}H NMR spectra analysis of the epoxide-opening cascade cyclization of *trans*-**1** (bottom) and *trans*,*anti*-**1** (top) catalyzed by π-acidic catalyst **10**. a) Full ^{1}H NMR spectra. b) Zoomed ^{1}H NMR spectra. c) Decoded NMR fingerprint region for products of *trans* diepoxide substrates.

a)

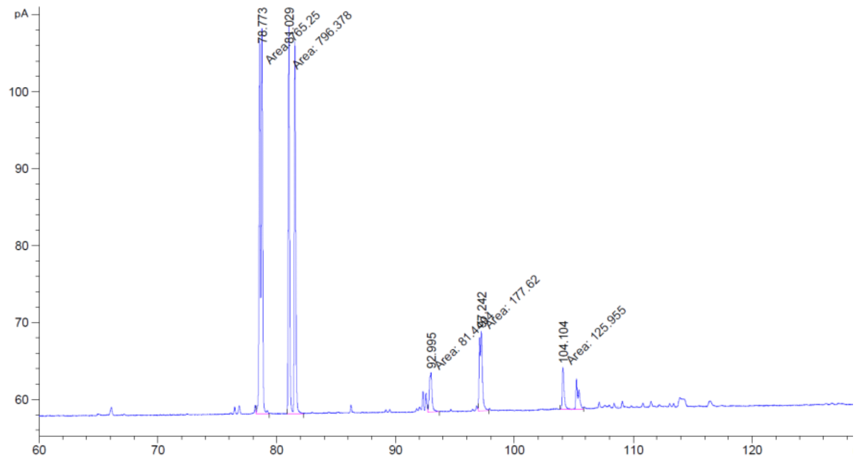

Product	Ret. time (min)	Area (pA*s)	Area (%)
BB	78.773	765.2	39.3
BB	81.029	796.4	40.9
BA	92.995	81.4	4.2
BA	97.242	177.6	9.1
AA	104.104	126.0	6.5

b)

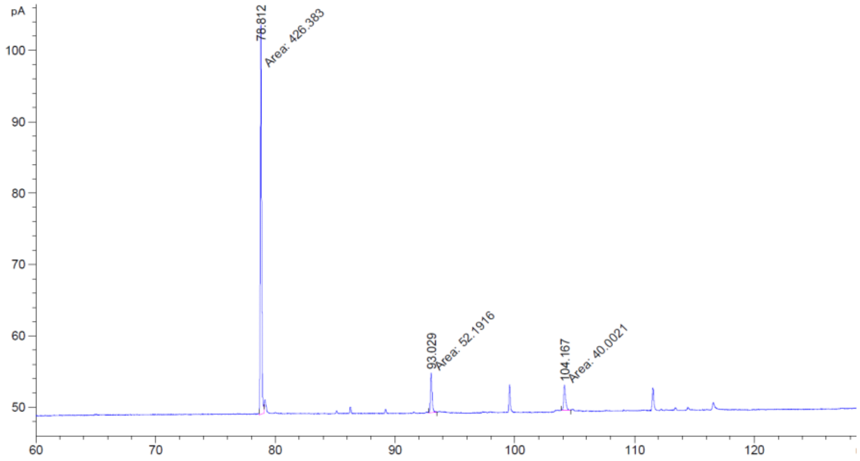

Product	Ret. time (min)	Area (pA*s)	Area (%)
BB	78.812	426.4	82.2
BA	93.029	52.2	10.1
AA	104.167	40.0	7.7

Figure S19. GC analysis of epoxides a) *trans*-**1** and b) *trans*,*anti*-**1** opening catalyzed by anion-π catalyst **10**.

5. Kinetics analysis

*Procedures for anion-π catalyst **10**.*

General procedure A. To a solution of *cis*-**1**, *cis,syn*-**1**, *trans*-**1** or *trans,anti*-**1** diepoxide substrate (18.1 mg, 75 µmol) in CD_2Cl_2 (50 µL) was added catalyst **10** (8.4 mg, 7.5 µmol), then the mixture was stirred at rt. The reaction was monitored by ^{1}H NMR spectroscopy.

Kinetic studies. Concentrations of products were estimated from the consumption of the substrate and were plotted against time. Here pseudo-first-order conditions are assumed for the analysis of the autocatalysis,[S9] and the reaction rate (r) can be expressed as

$$r = k_1[\mathrm{R}] + k_2[\mathrm{R}][\mathrm{P}] \qquad \text{(S1)}$$

where k_1 and k_2 are the rate constants corresponding to the non(auto)catalytic and the (auto)catalytic mechanisms, respectively. Assuming first order in both reactant (R) and autocatalytic product (P), and

$$[\mathrm{P}] = [\mathrm{R}]_0 - [\mathrm{R}] \qquad \text{(S2)}$$

then,

$$[\mathrm{P}] = [\mathrm{R}]_0 \times \left(1 - \frac{b+k_1}{b+k_1 \exp{(k_1+b)t}}\right) \qquad \text{(S3)}$$[S10]

where,

$$b = [\mathrm{R}]_0 k_2 \qquad \text{(S4)}$$

The rate constants k_1 and k_2 were obtained by fitting the data to the equations (S3) and (S4). The substrate half-lifetimes (t_{50}) were obtained using Equation (S5).

$$t_{50} = \ln(b/k_1+2)/(b+k_1) \qquad \text{(S5)}$$

Procedures for AcOH.

General procedure B. To a solution of *cis*-**1**, *cis,syn*-**1**, *trans*-**1** or *trans,anti*-**1** diepoxide substrate (18.1 mg, 75 μmol) in CD_2Cl_2 (600 μL) was added AcOH (21.4 μL, 374 μmol), then the mixture was stirred at 40 ºC. The reaction was monitored by ^{1}H NMR spectroscopy.

Kinetic studies. The pseudo-first-order rate constant (*k*) was estimated by fitting the data to the Equation (S6):

$$[P] = [R]_0 - ([R]_0 - [P]_0)\cdot\exp(-kt) \qquad \text{(S6)}$$

[P] starts at $[P]_0 = 0$, then goes up to $[R]_0$ with one phase. The rate constants *k* was obtained by fitting the data to Equation (S6). The substrate half-lifetimes (t_{50}) were obtained using Equation (S7).

$$t_{50} = \ln[2\cdot([R]_0 - [P]_0)/[R]_0]/k \qquad \text{(S7)}$$

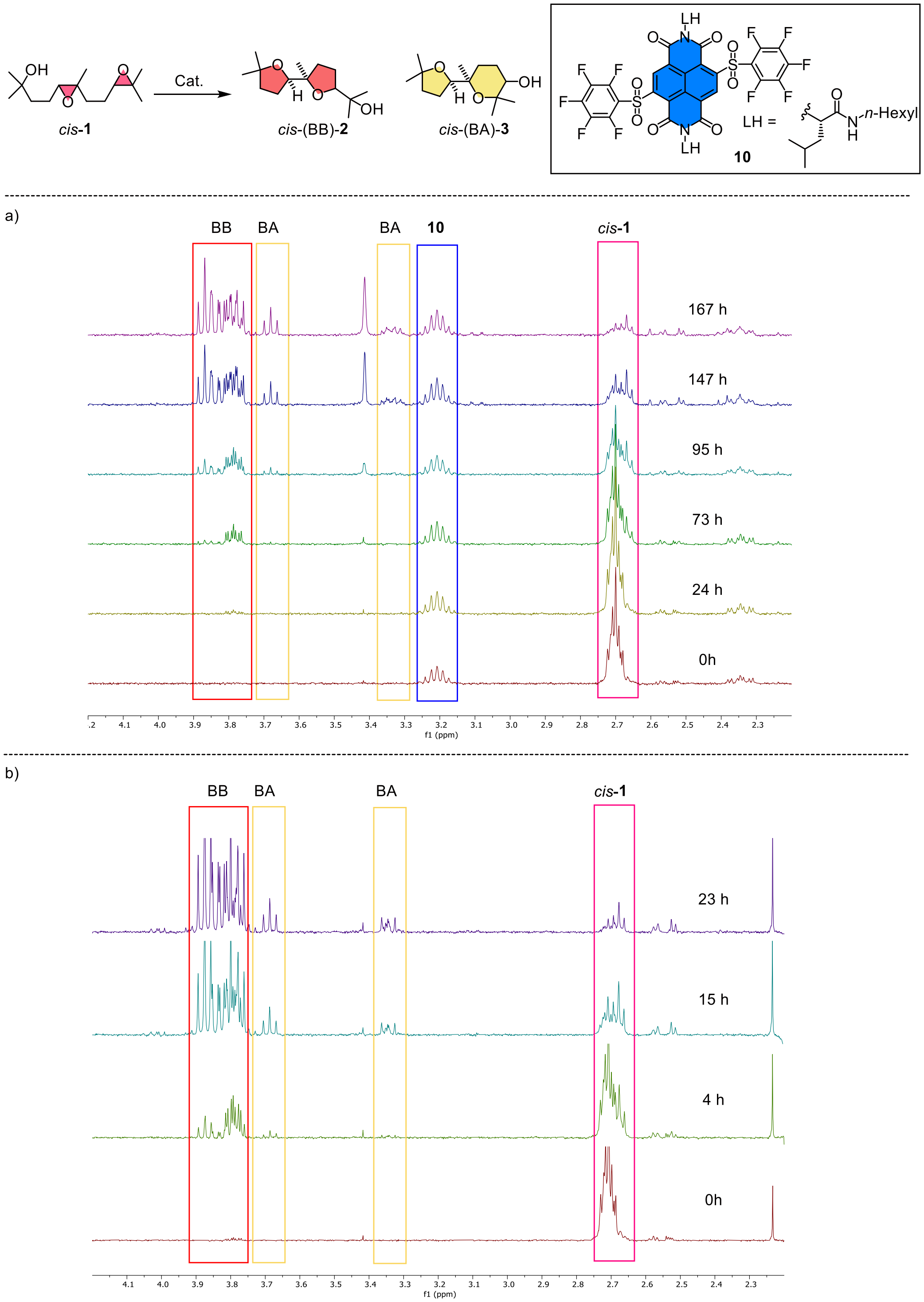

Figure S20. Kinetic studies of *cis*-**1** with a) anion-π catalyst **10** and b) Brønsted acid catalyst. AcOH.

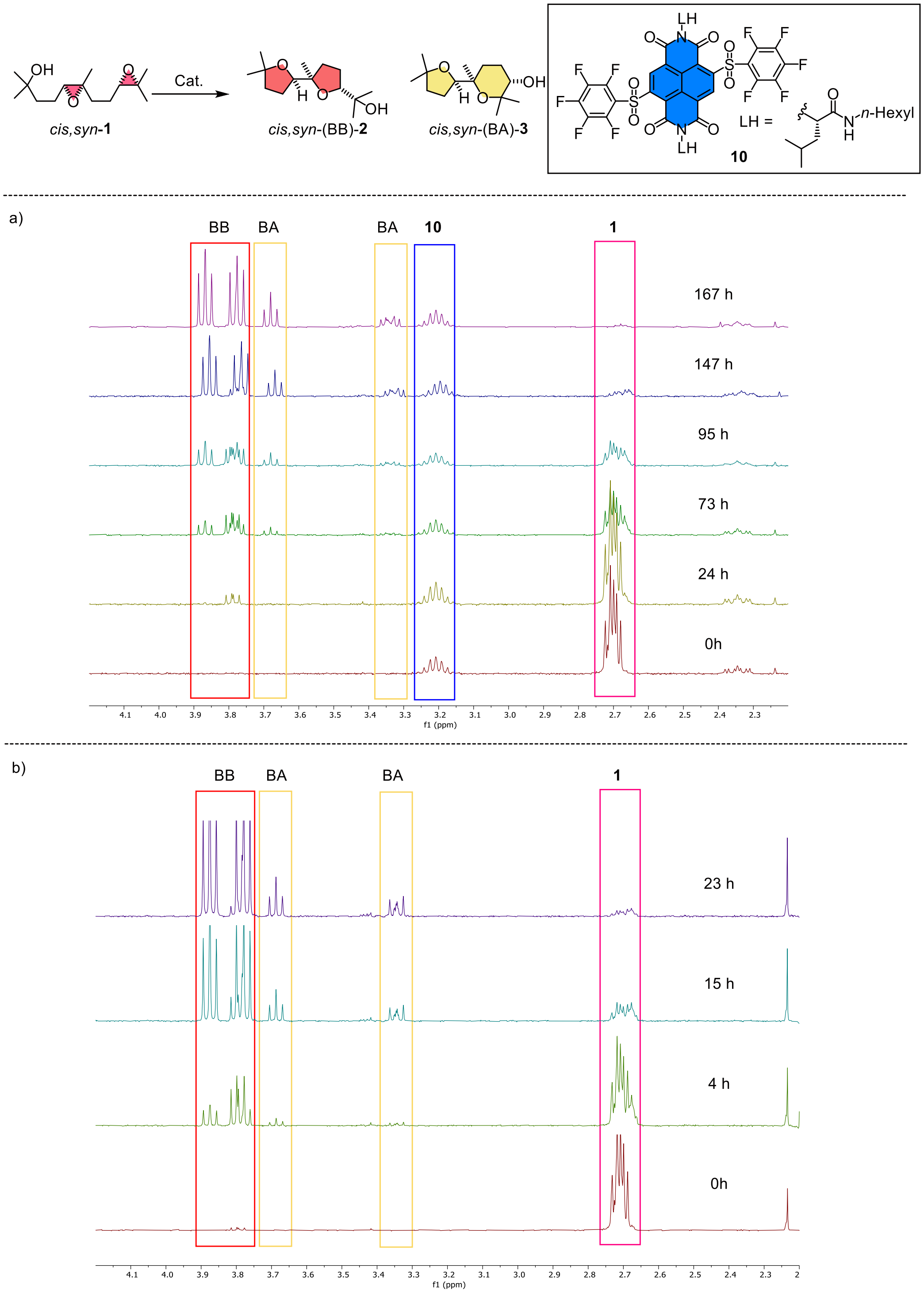

Figure S21. Kinetic studies of *cis*,*syn*-**1** with a) anion-π catalyst **10** and b) Brønsted acid catalyst AcOH.

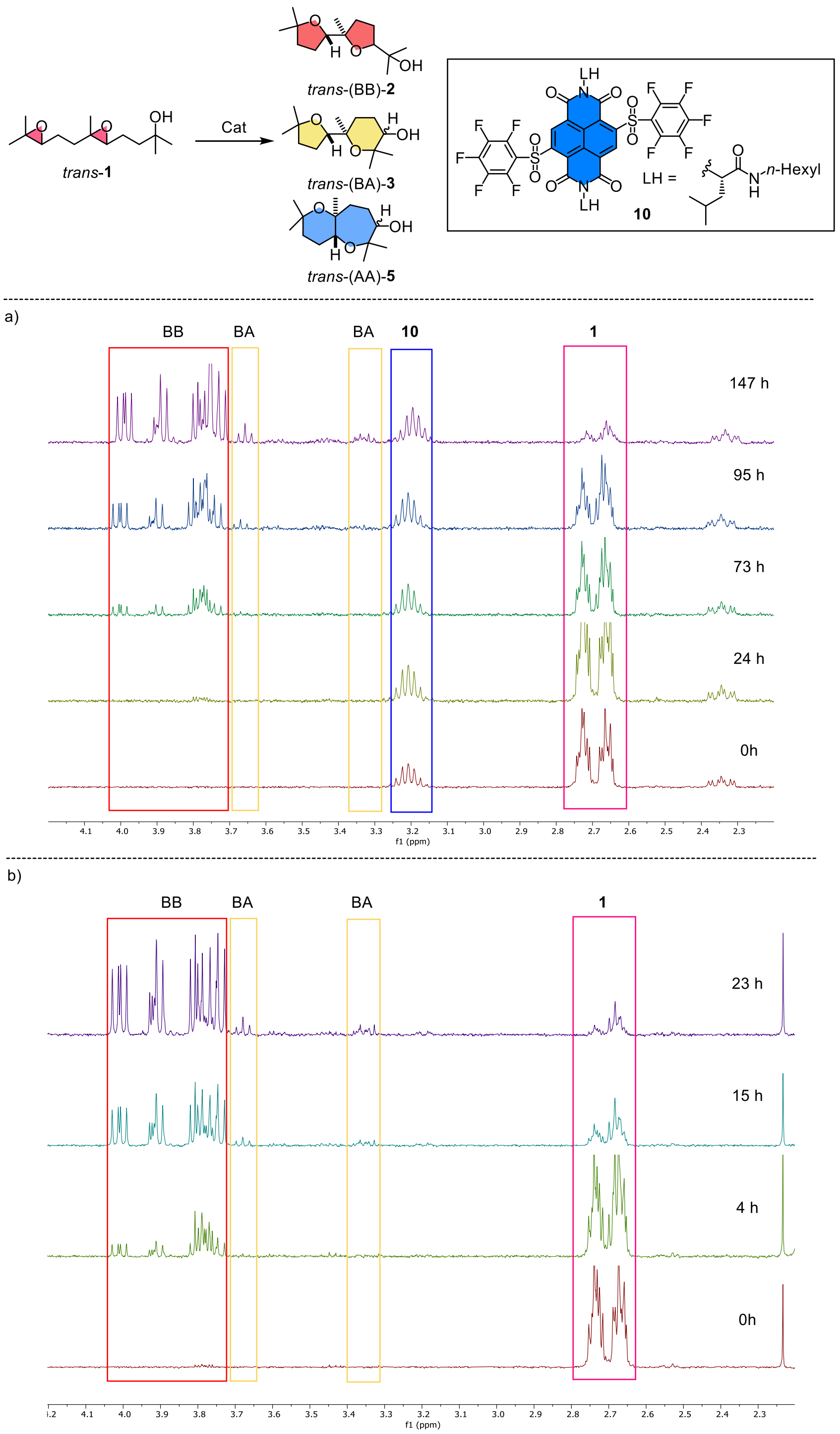

Figure S22. Kinetic studies of *trans*-**1** with a) anion-π catalyst **10** and b) Brønsted acid catalyst AcOH.

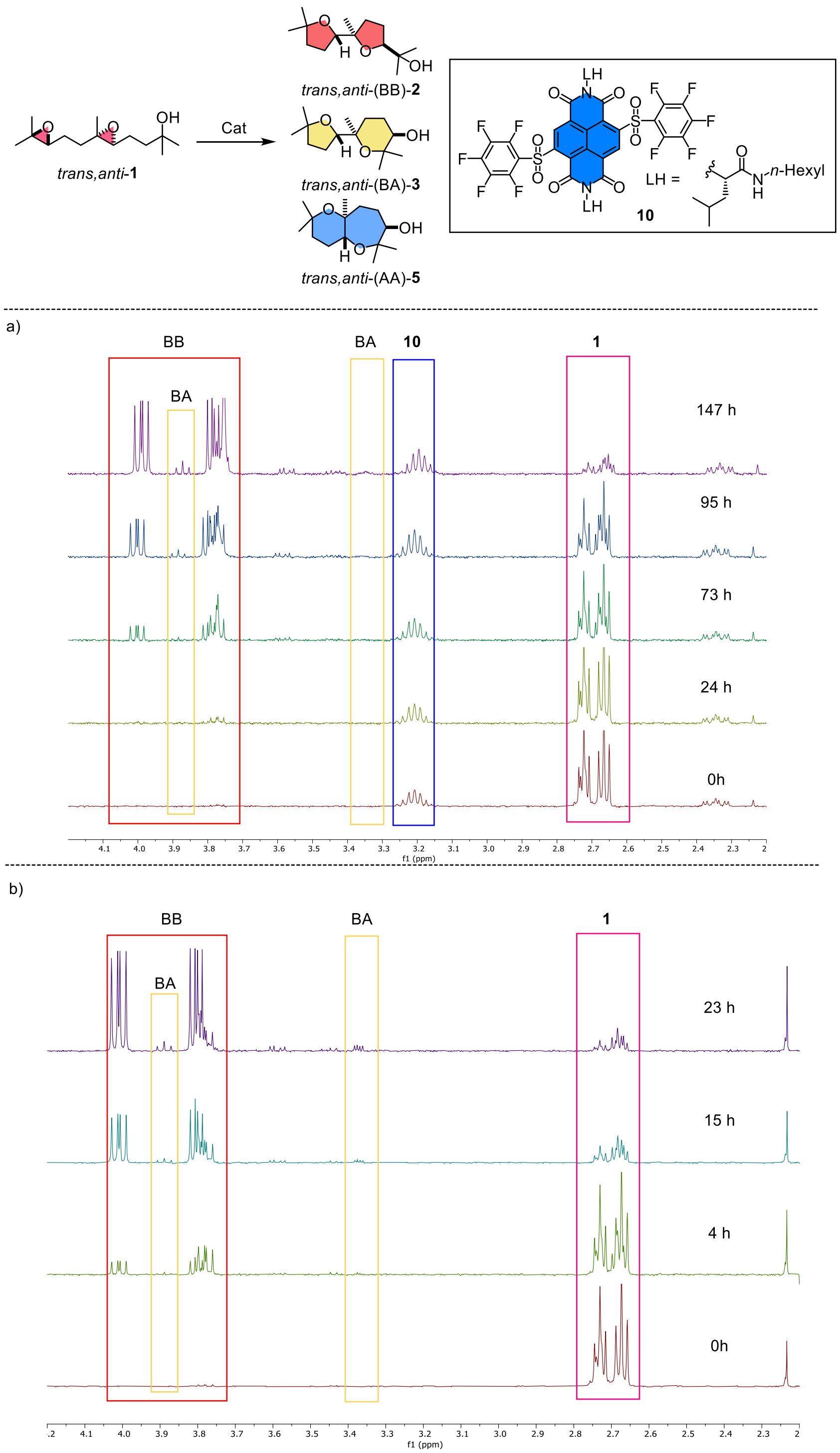

Figure S23. Kinetic studies of *trans,anti*-**1** with a) anion-π catalyst **10** and b) Brønsted acid catalyst AcOH.

6. NMR spectra

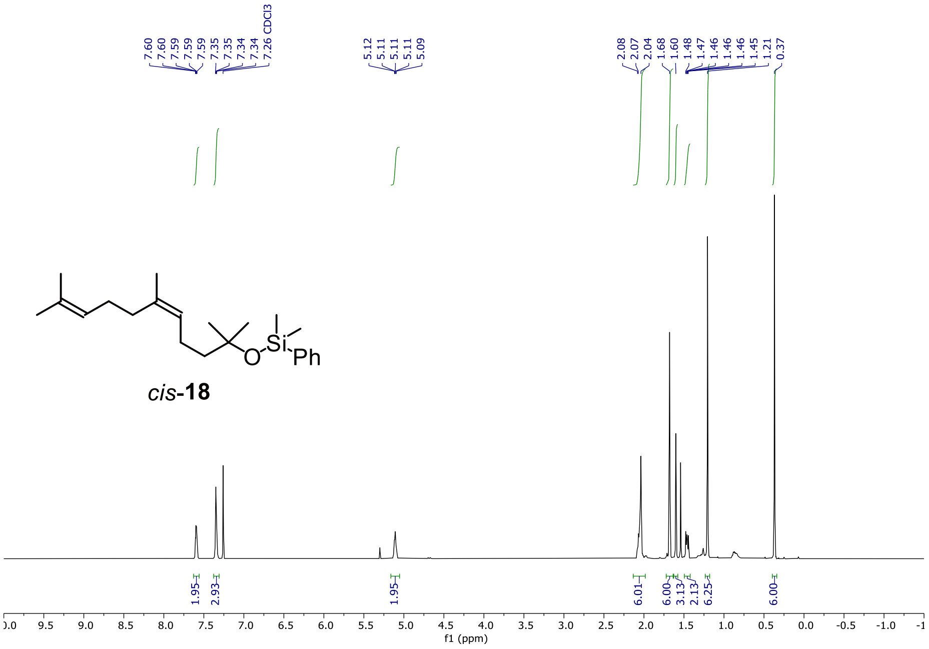

Figure S24. 500 MHz ^{1}H NMR spectrum of *cis*-**18** in $CDCl_3$.

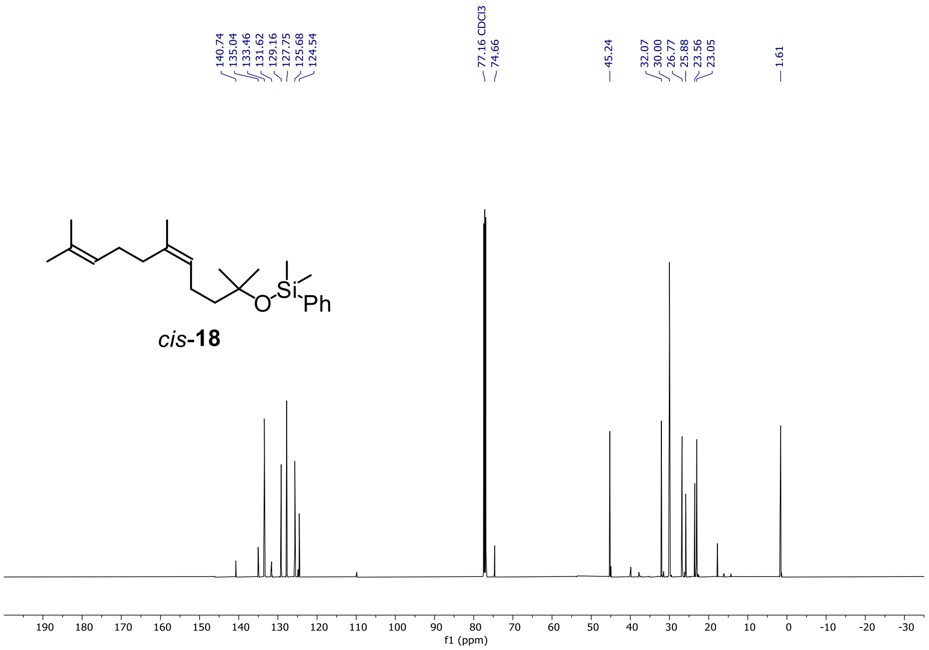

Figure S25. 125 MHz ^{13}C NMR spectrum of *cis*-**18** in $CDCl_3$.

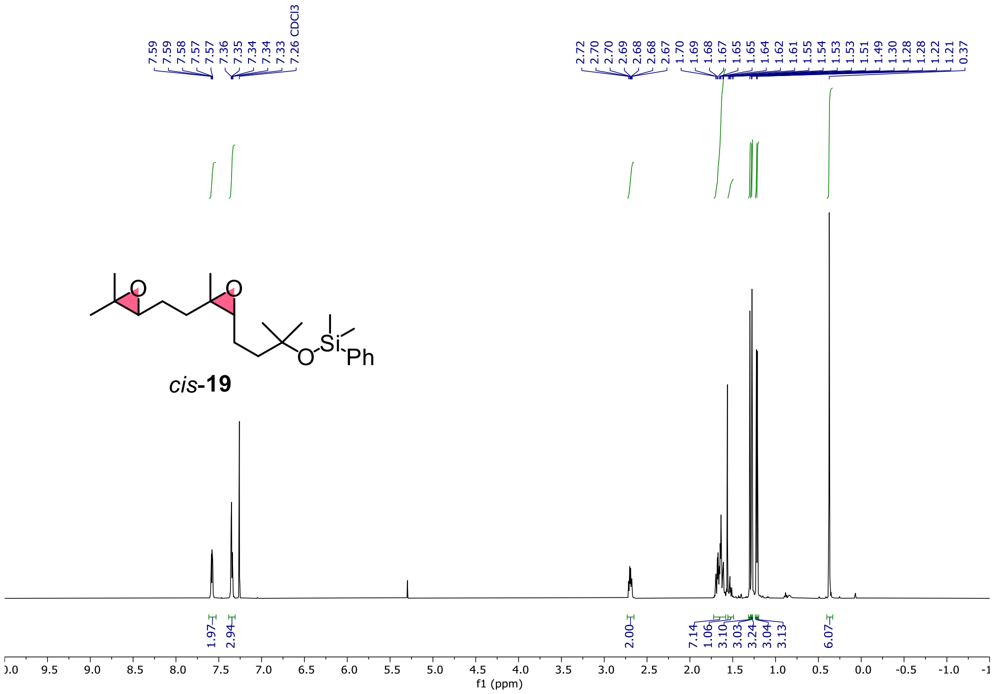

Figure S26. 500 MHz ^{1}H NMR spectrum of *cis*-**19** in $CDCl_3$.

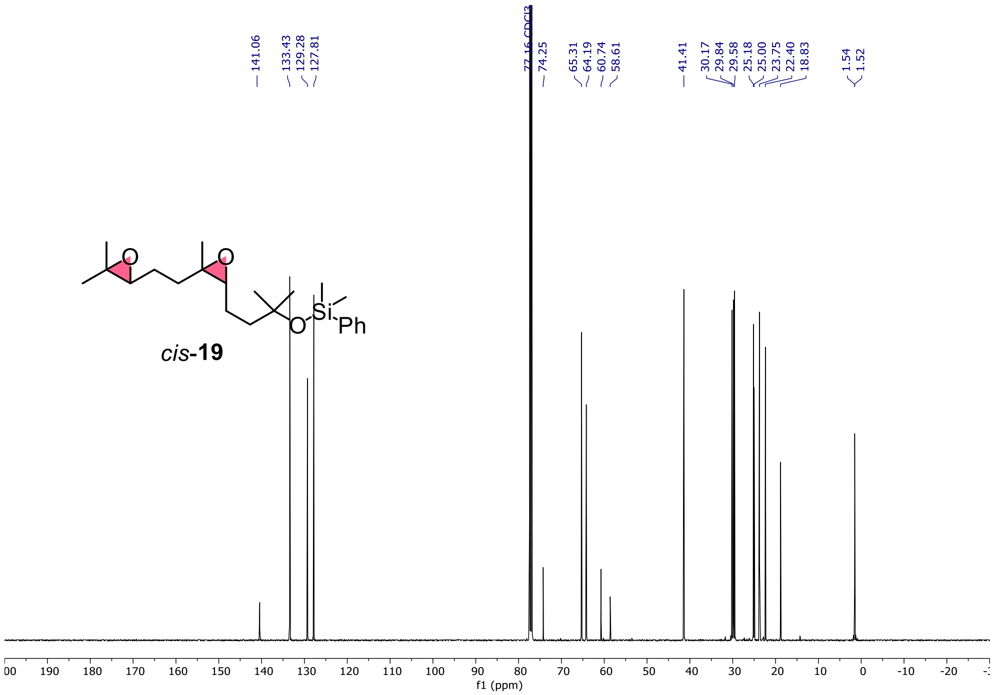

Figure S27. 125 MHz ^{13}C NMR spectrum of *cis*-**19** in $CDCl_3$.

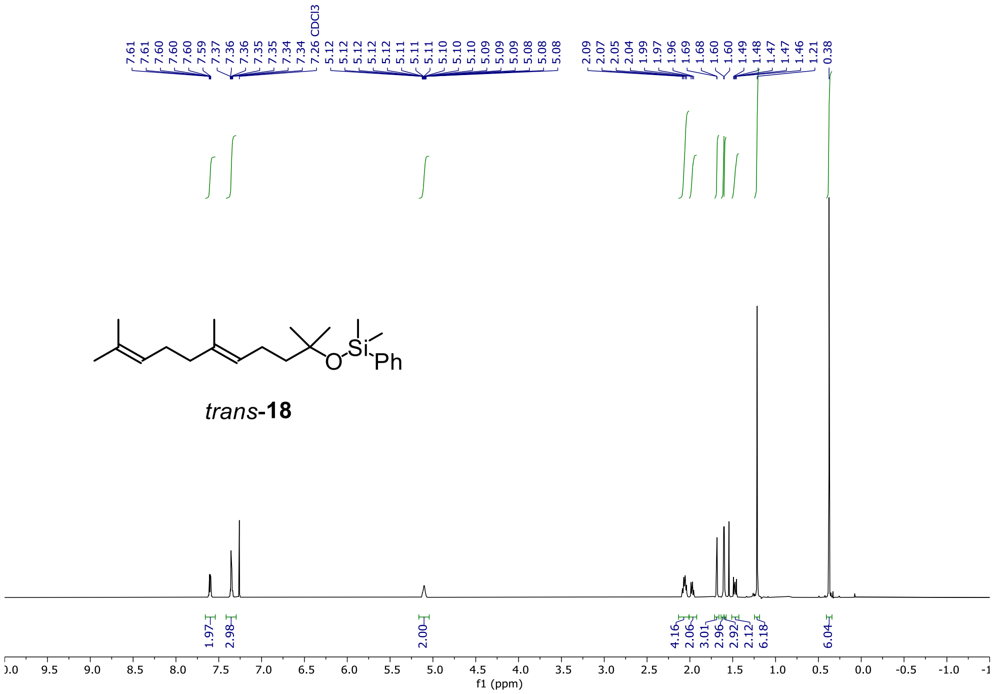

Figure S28. 500 MHz ^{1}H NMR spectrum of *trans*-**18** in $CDCl_3$.

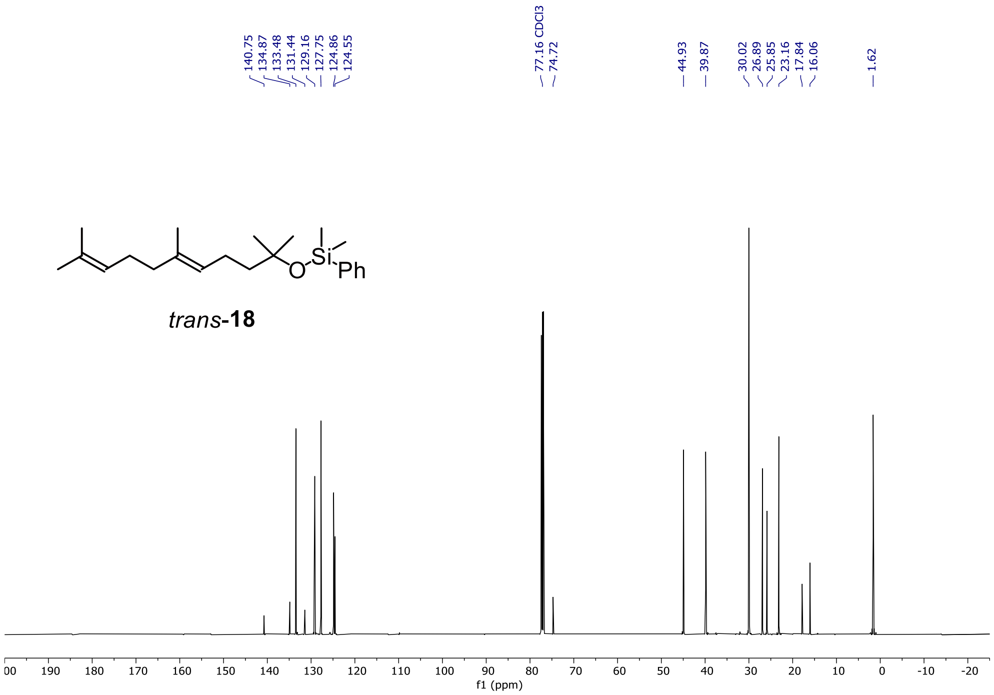

Figure S29. 125 MHz ^{13}C NMR spectrum of *trans*-**18** in $CDCl_3$.

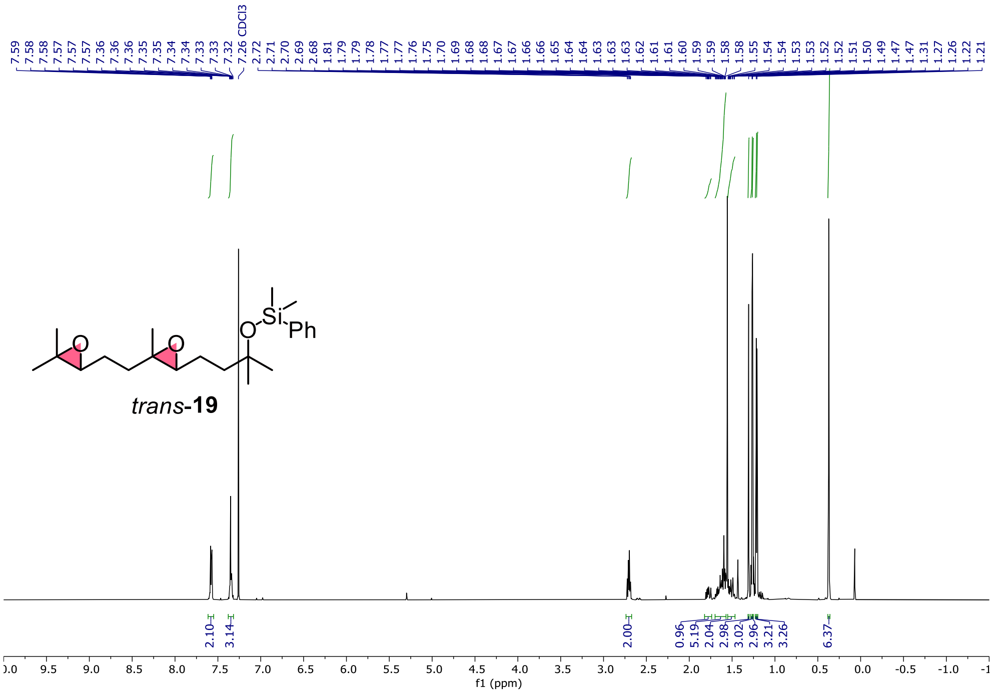

Figure S30. 500 MHz ^{1}H NMR spectrum of *trans*-**19** in $CDCl_3$.

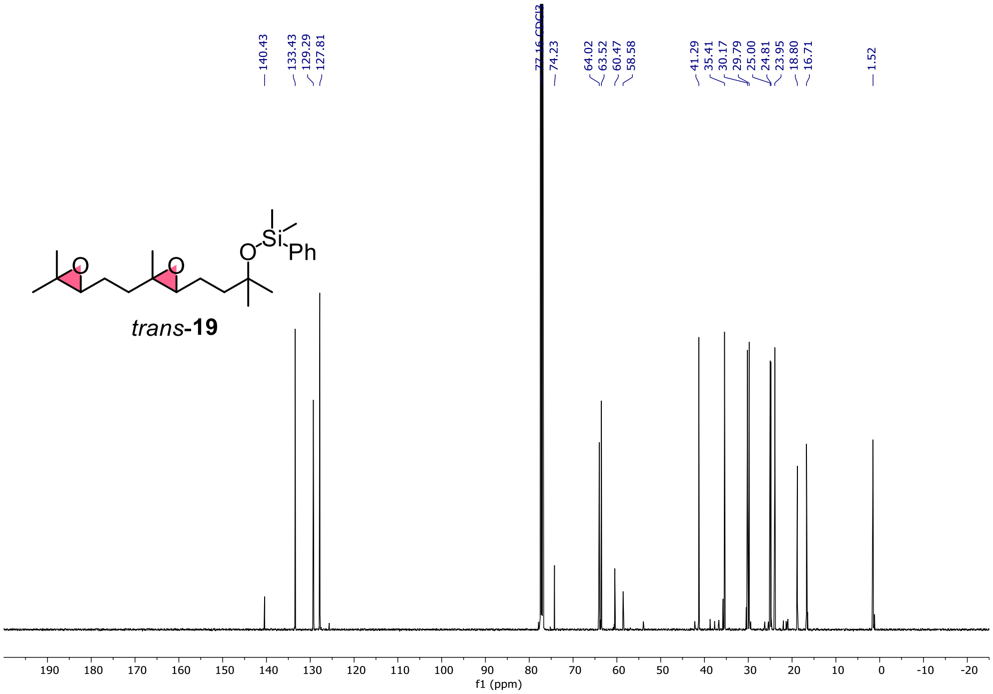

Figure S31. 125 MHz ^{13}C NMR spectrum of *trans*-**19** in $CDCl_3$.

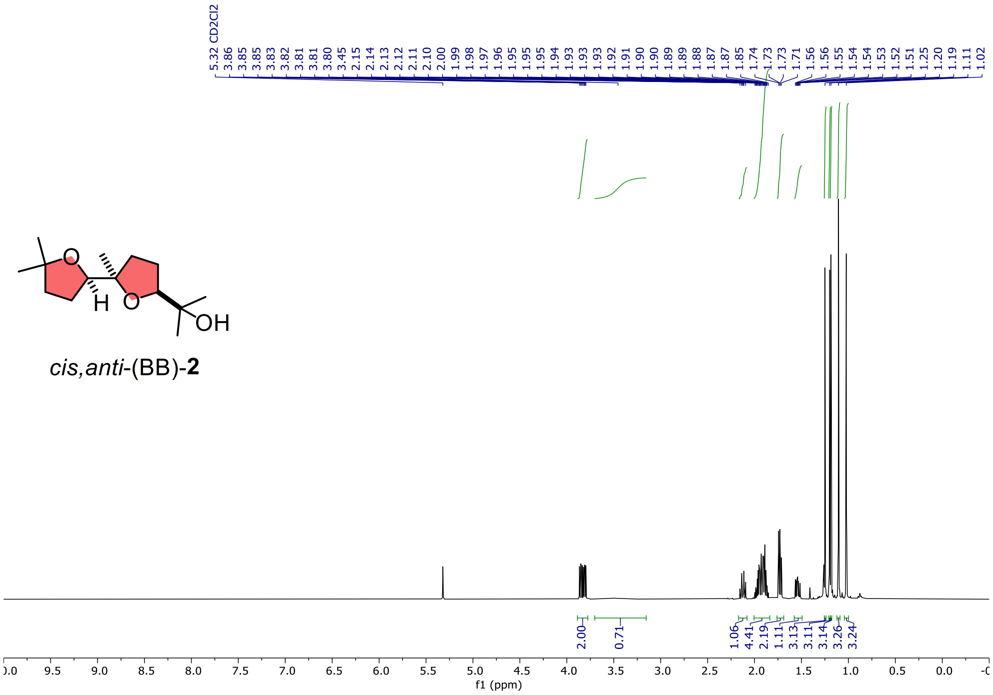

Figure S32. 500 MHz ^{1}H NMR spectrum of *cis,anti*-(BB)-**2** in CD_2Cl_2.

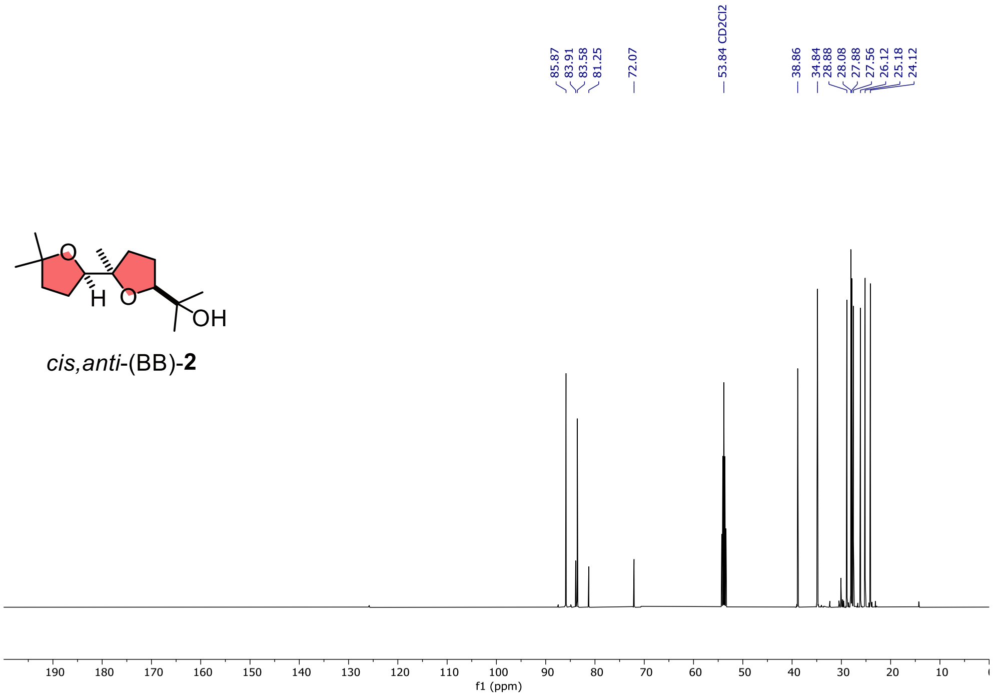

Figure S33. 125 MHz ^{13}C NMR spectrum of *cis,anti*-(BB)-**2** in CD_2Cl_2.

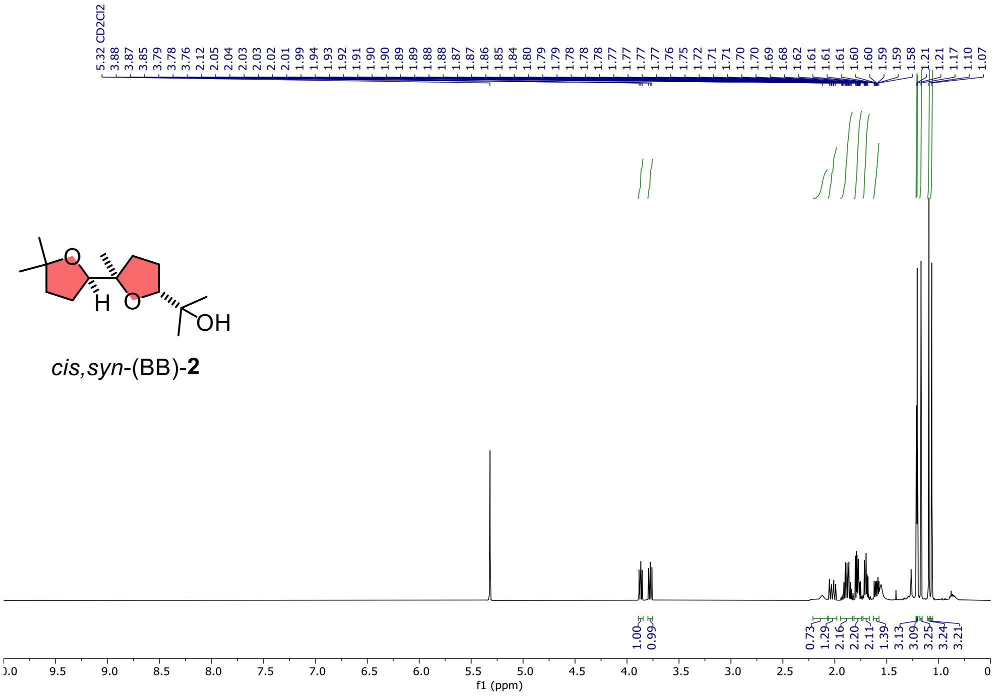

Figure S34. 500 MHz ^{1}H NMR spectrum of *cis*,*syn*-(BB)-**2** in CD_2Cl_2.

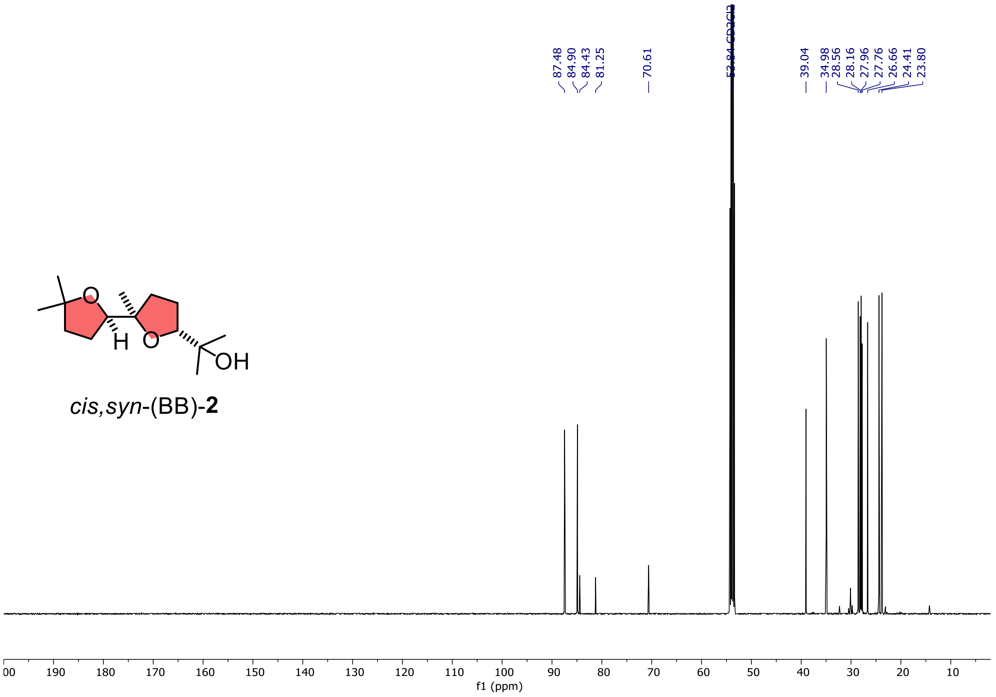

Figure S35. 125 MHz ^{13}C NMR spectrum of *cis*,*syn*-(BB)-**2** in CD_2Cl_2.

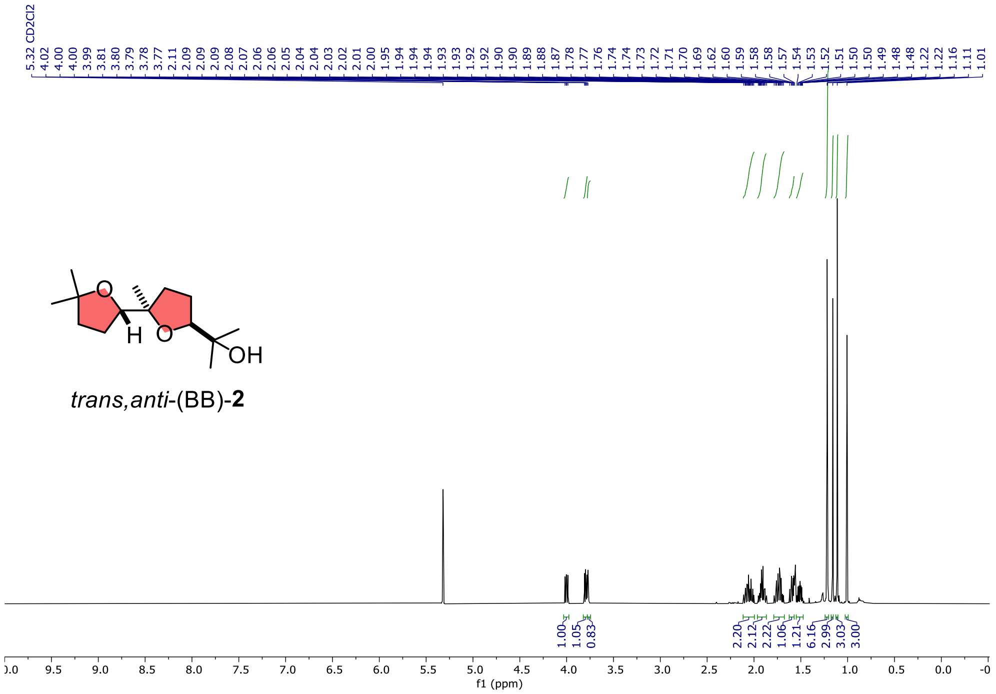

Figure S36. 500 MHz ^{1}H NMR spectrum of *trans,anti*-(BB)-**2** in CD_2Cl_2.

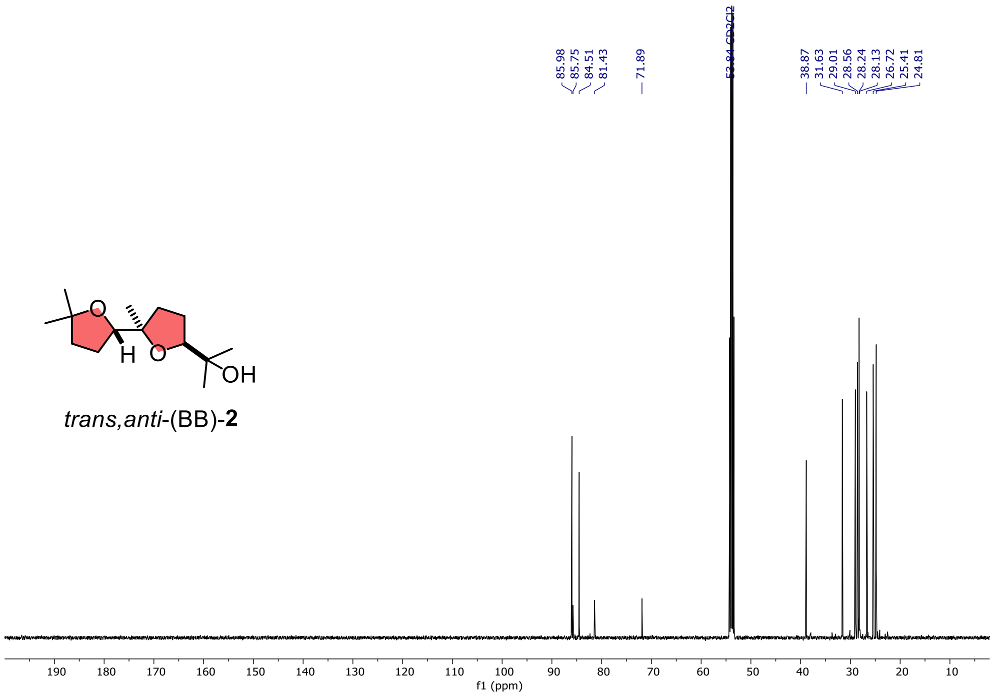

Figure S37. 125 MHz ^{13}C NMR spectrum of *trans,anti*-(BB)-**2** in CD_2Cl_2.

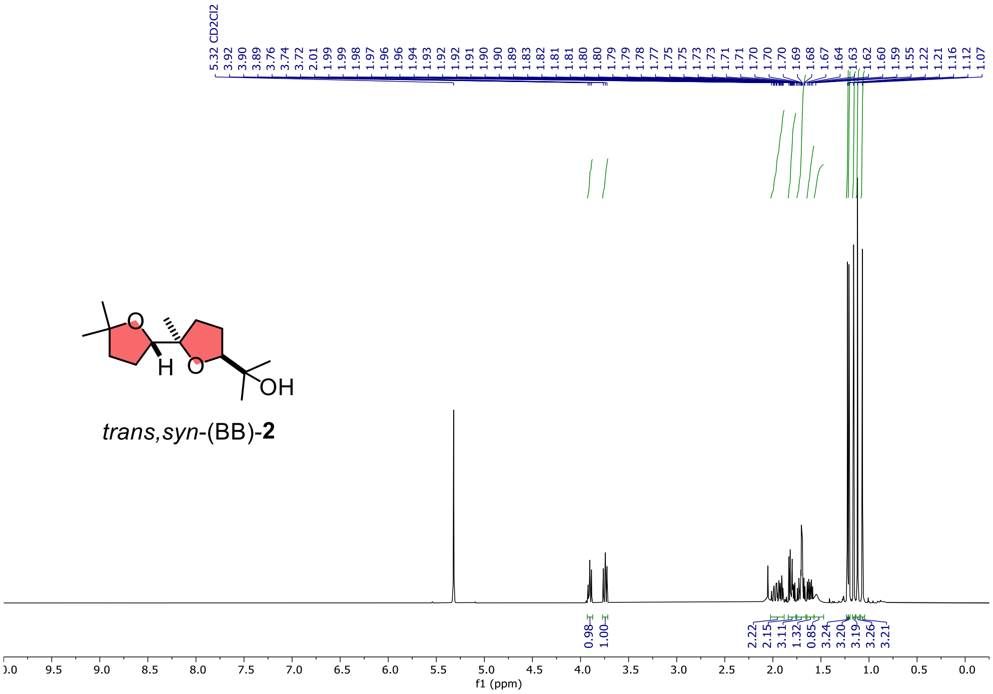

Figure S38. 400 MHz ^{1}H NMR spectrum of *trans*,*syn*-(BB)-**2** in CD_2Cl_2.

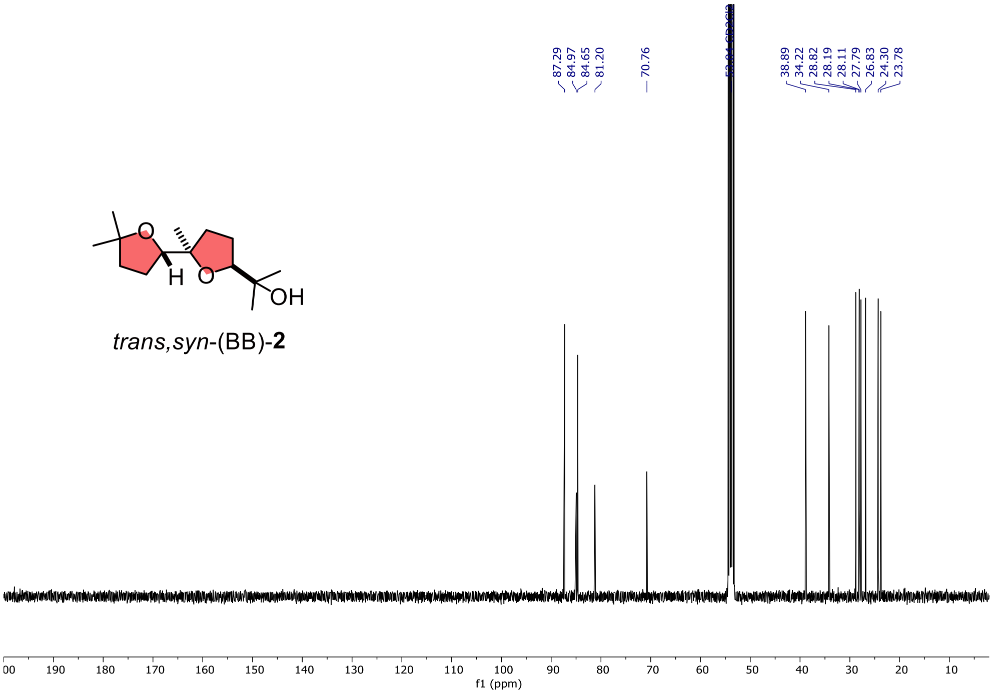

Figure S39. 100 MHz ^{13}C NMR spectrum of *trans*,*syn*-(BB)-**2** in CD_2Cl_2.

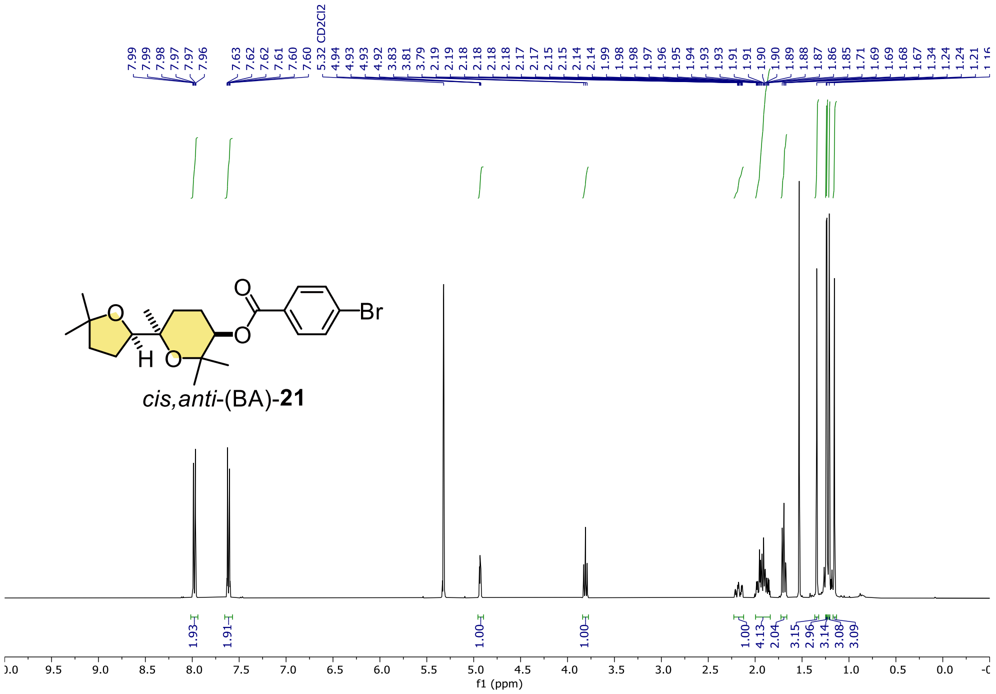

Figure S40. 400 MHz ^{1}H NMR spectrum of *cis*,*anti*-(BA)-**21** in CD_2Cl_2.

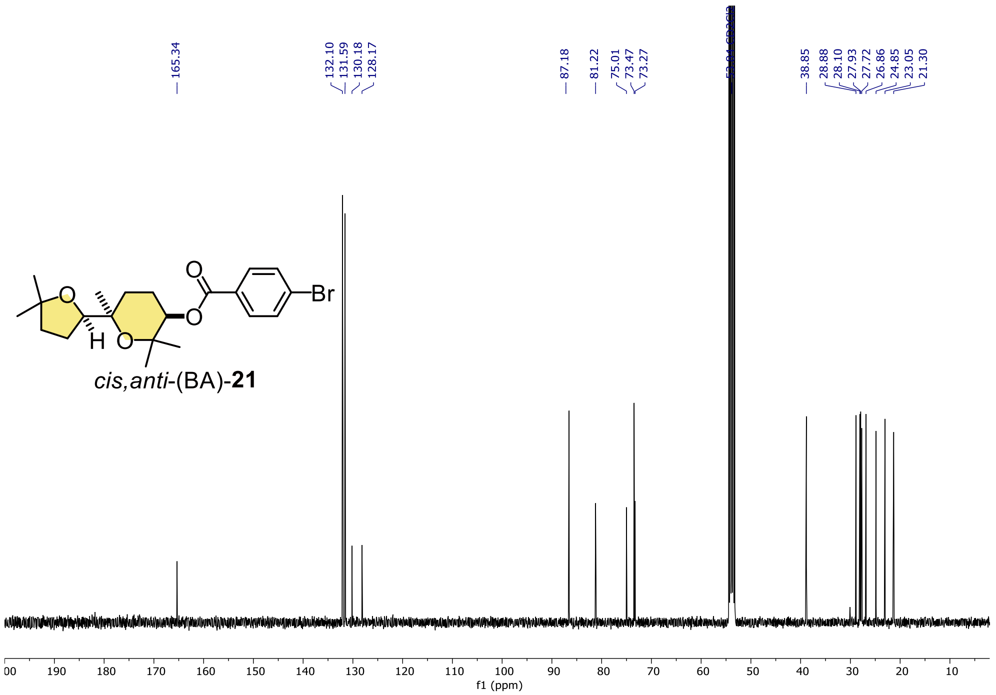

Figure S41. 100 MHz ^{13}C NMR spectrum of spectrum of *cis*,*anti*-(BA)-**21** in CD_2Cl_2.

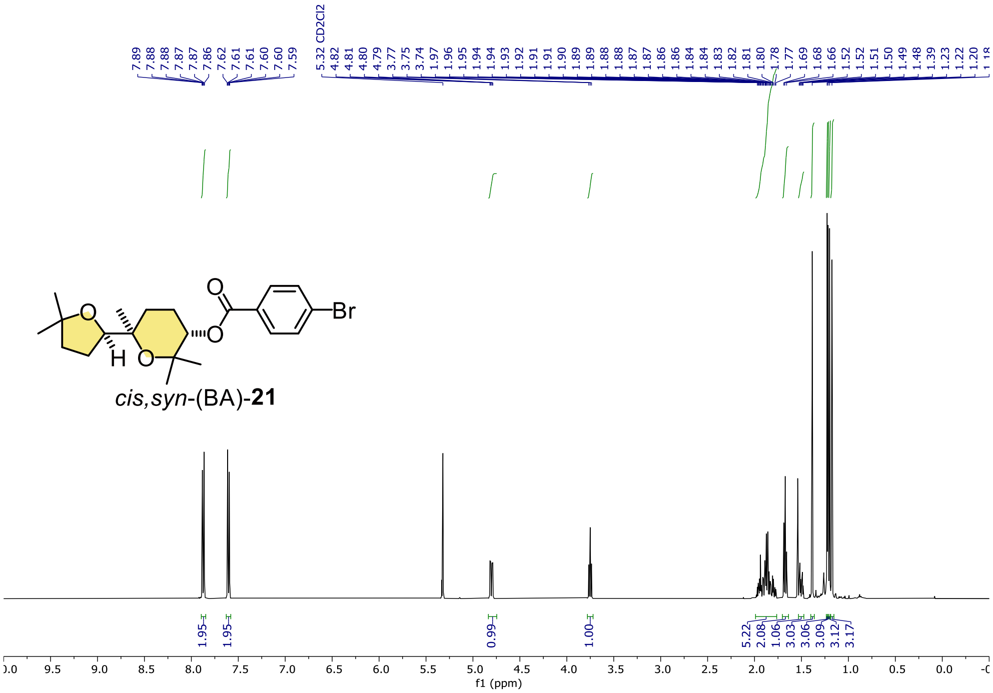

Figure S42. 500 MHz ^{1}H NMR spectrum of *cis*,*syn*-(BA)-**21** in CD_2Cl_2.

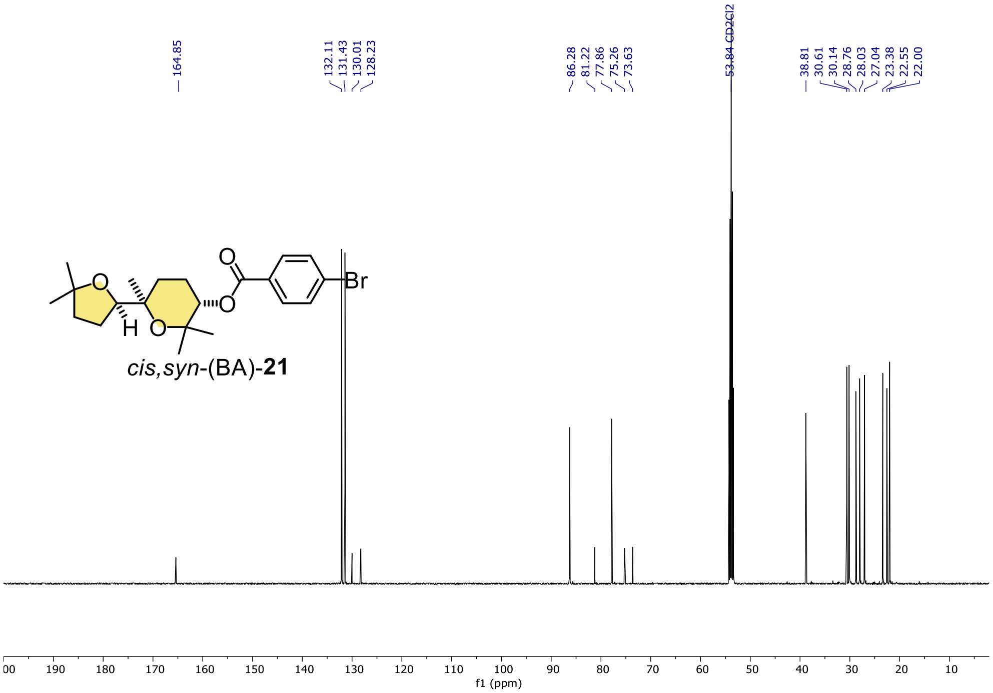

Figure S43. 125 MHz ^{13}C NMR spectrum of *cis*,*syn*-(BA)-**21** in CD_2Cl_2.

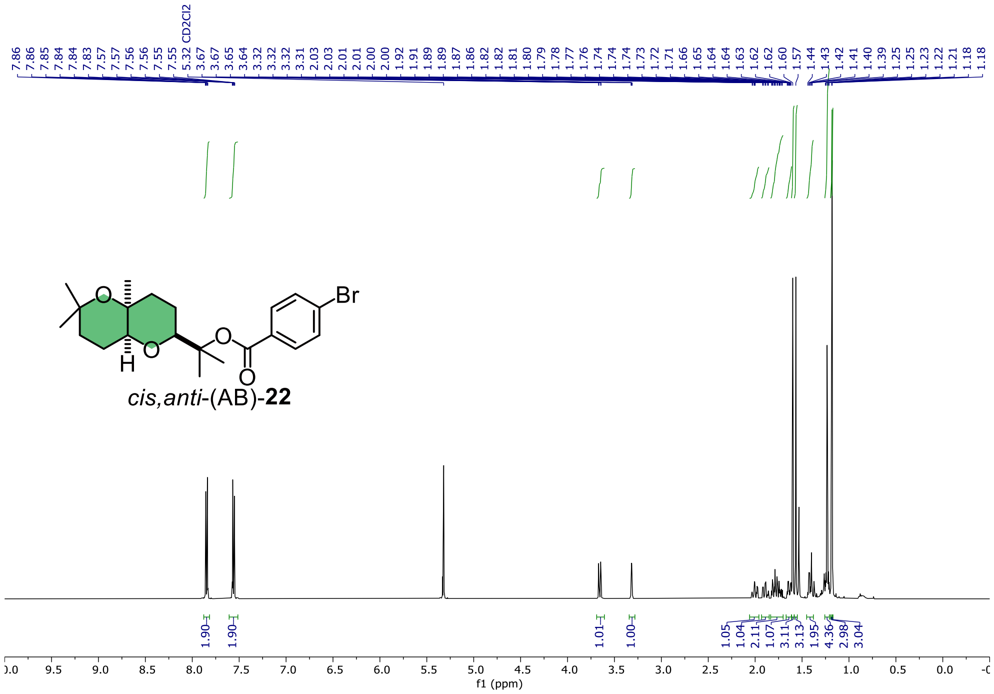

Figure S44. 500 MHz spectrum of *cis,anti*-(AB)-**22** in CD_2Cl_2.

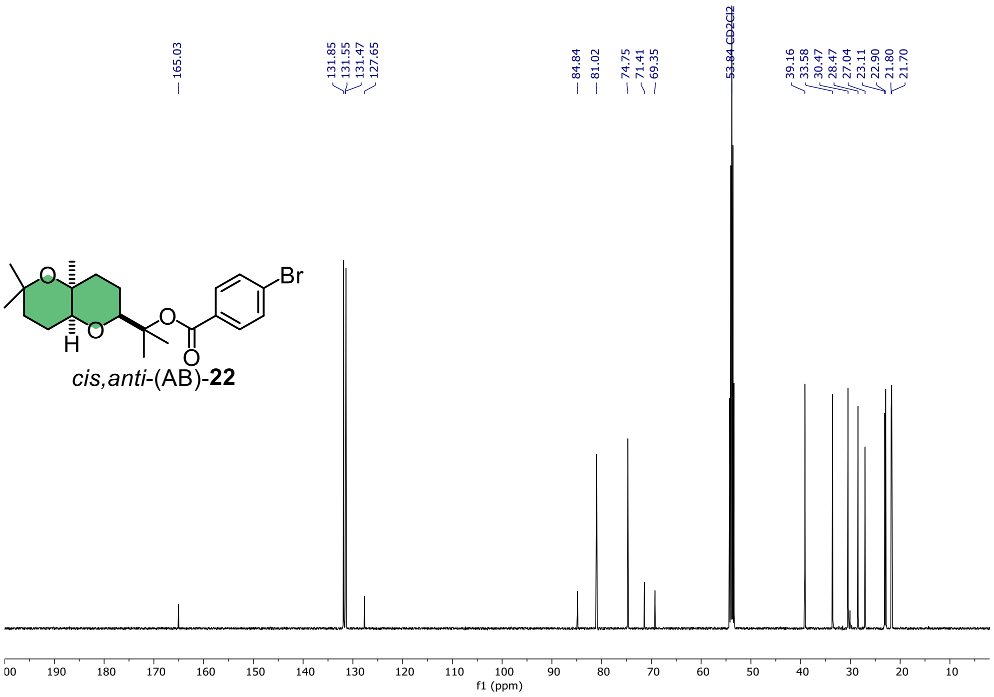

Figure S45. 125 MHz ^{13}C NMR spectrum of *cis,anti*-(AB)-**22** in CD_2Cl_2.

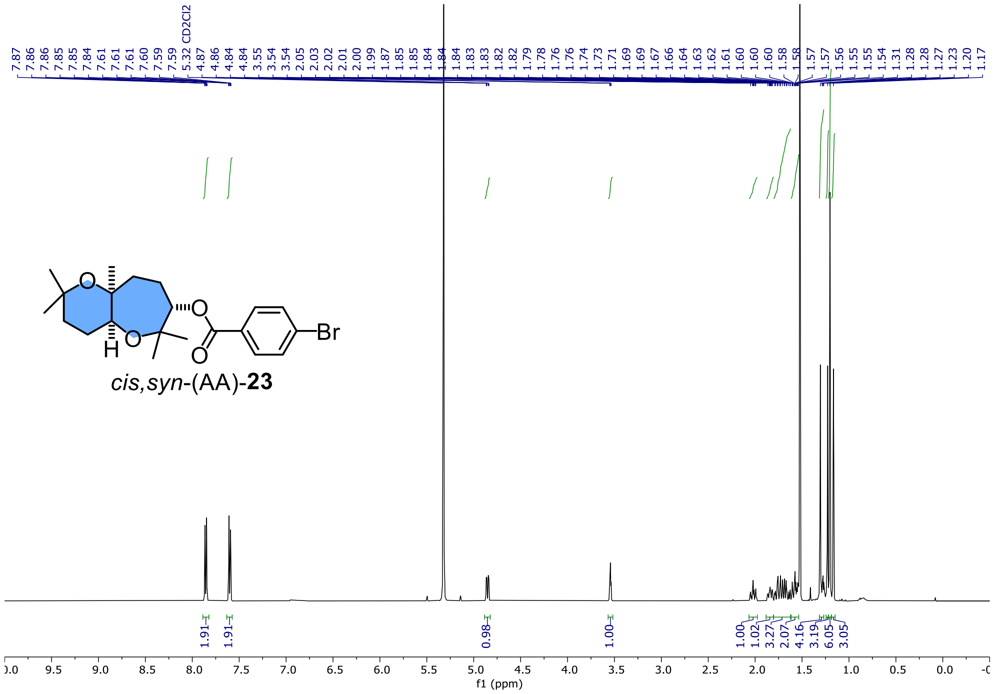

Figure S46. 500 MHz ^{1}H NMR spectrum of *cis*,*syn*-(AA)-**23** in CD_2Cl_2.

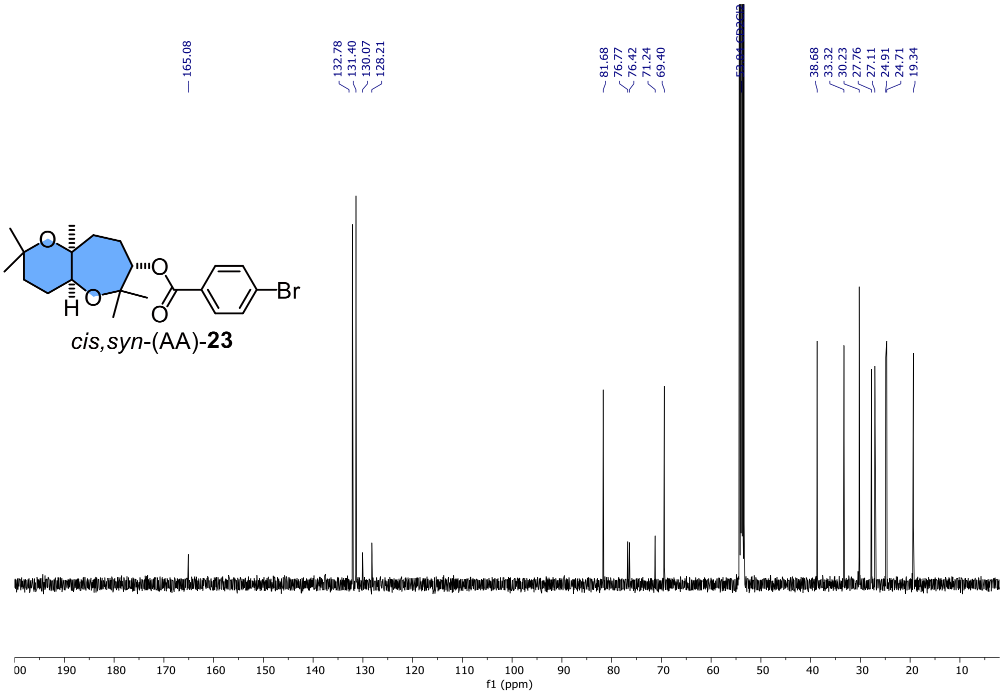

Figure S47. 125 MHz ^{13}C NMR spectrum of *cis*,*syn*-(AA)-**23** in CD_2Cl_2.

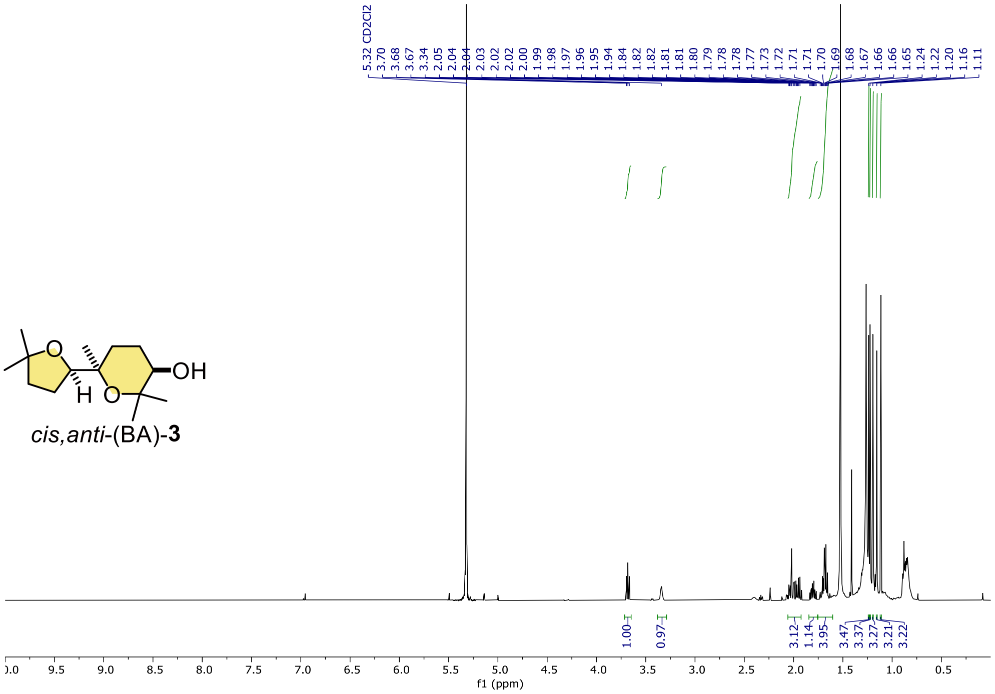

Figure S48. 500 MHz ^{1}H NMR spectrum of *cis,anti*-(BA)-**3** in CD_2Cl_2.

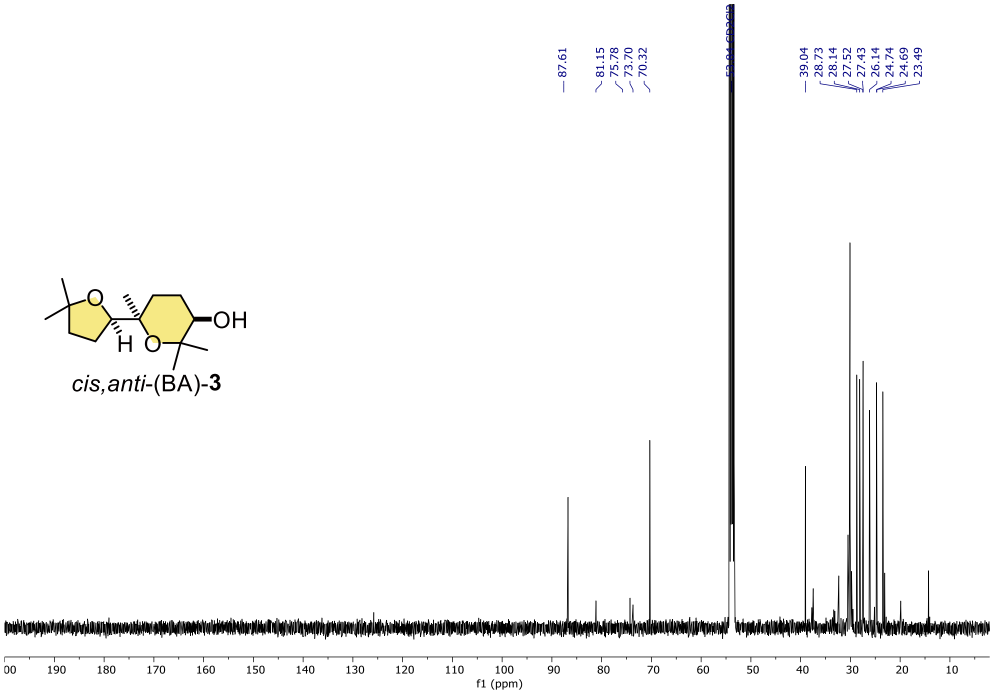

Figure S49. 125 MHz ^{13}C NMR spectrum of *cis,anti*-(BA)-**3** in CD_2Cl_2.

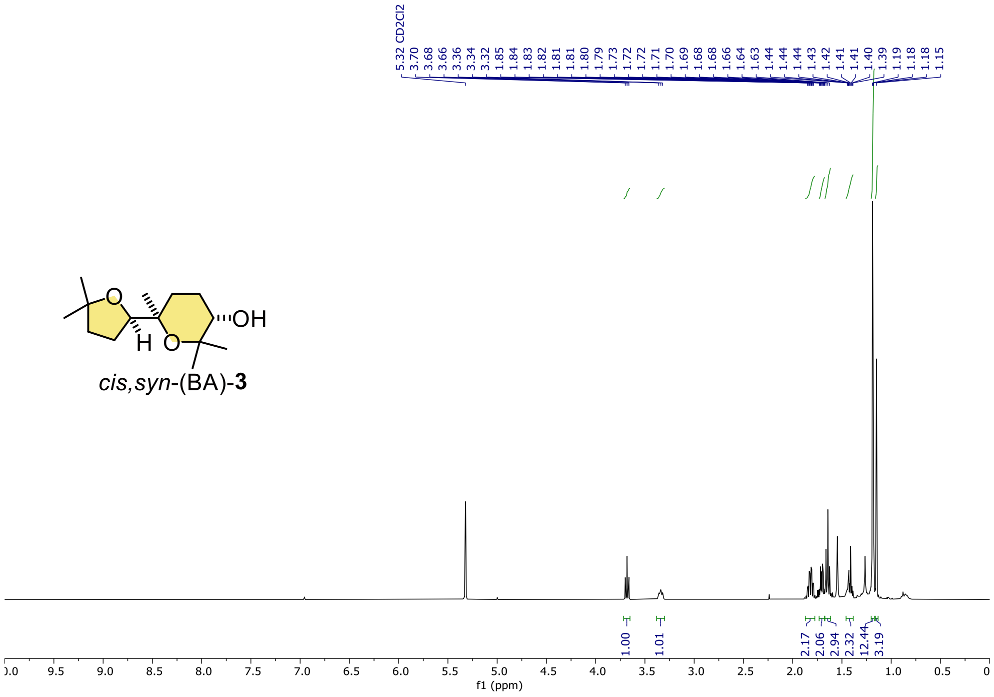

Figure S50. 400 MHz ^{1}H NMR spectrum of *cis,syn*-(BA)-**3** in CD_2Cl_2.

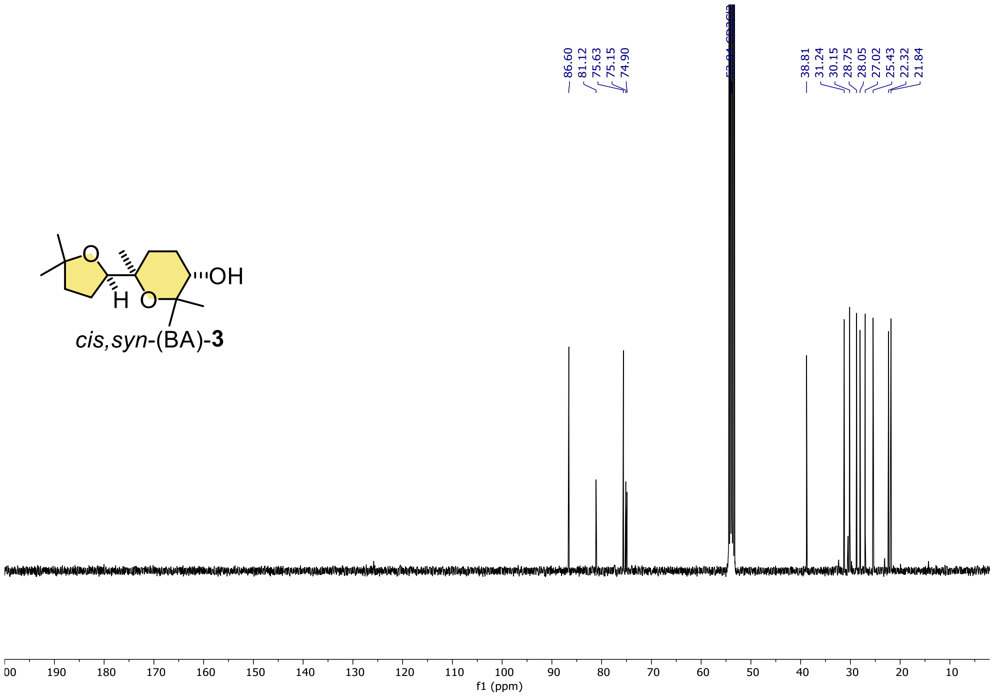

Figure S51. 100 MHz ^{13}C NMR spectrum of *cis,syn*-(BA)-**3** in CD_2Cl_2.

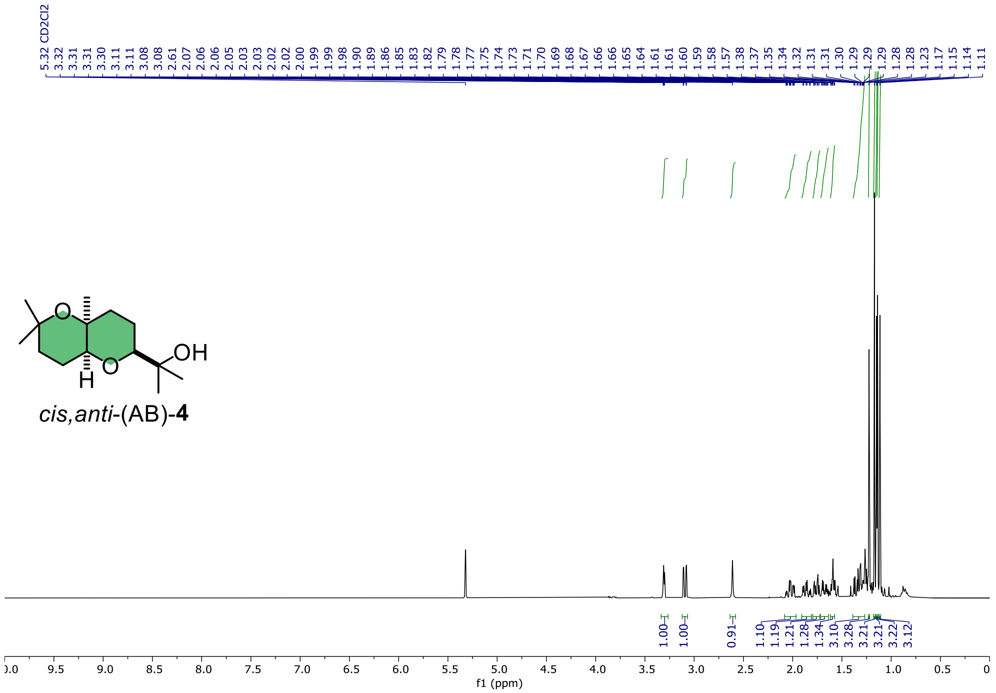

Figure S52. 400 MHz ^{1}H NMR spectrum of *cis*,*anti*-(AB)-**4** in CD_2Cl_2.

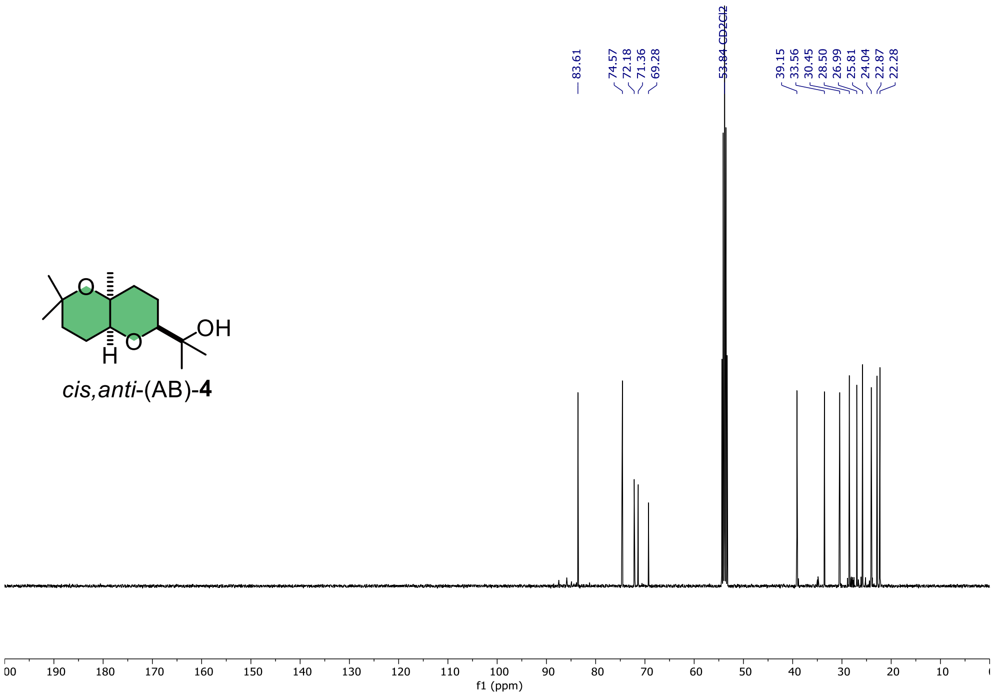

Figure S53. 100 MHz ^{13}C NMR spectrum of *cis*,*anti*-(AB)-**4** in CD_2Cl_2.

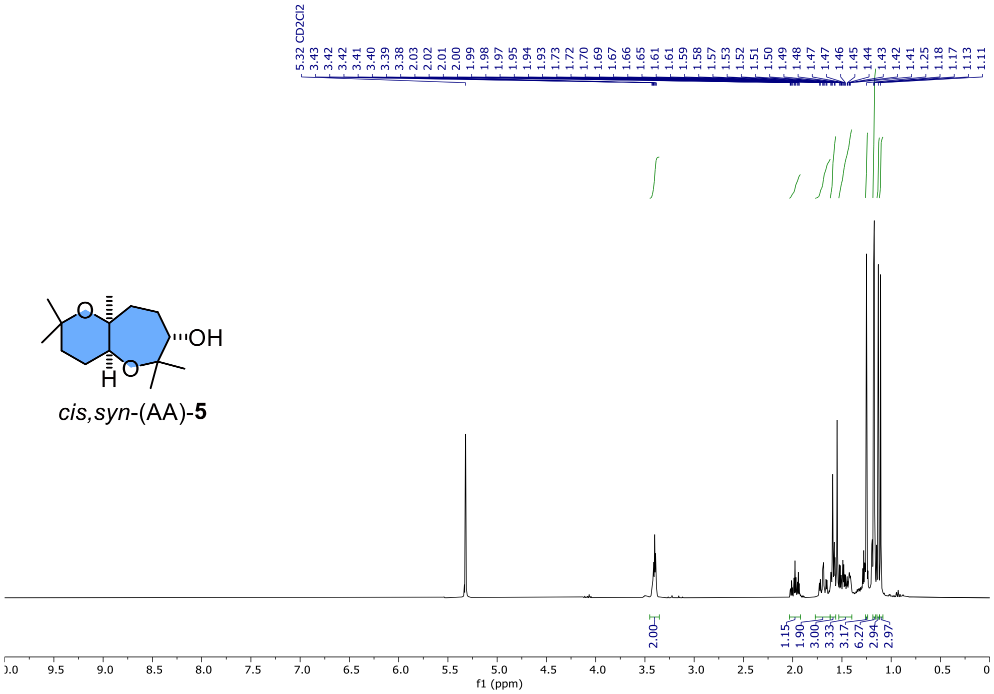

Figure S54. 400 MHz ^{1}H NMR spectrum of *cis,syn*-(AA)-**5** in CD_2Cl_2.

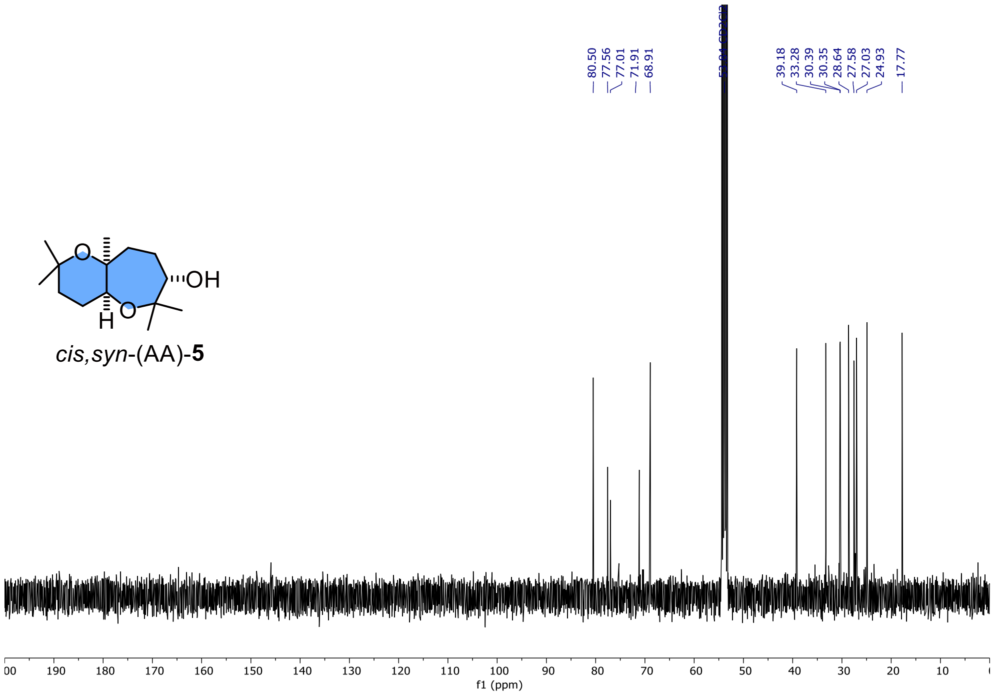

Figure S55. 100 MHz ^{13}C NMR spectrum of *cis,syn*-(AA)-**5** in CD_2Cl_2.

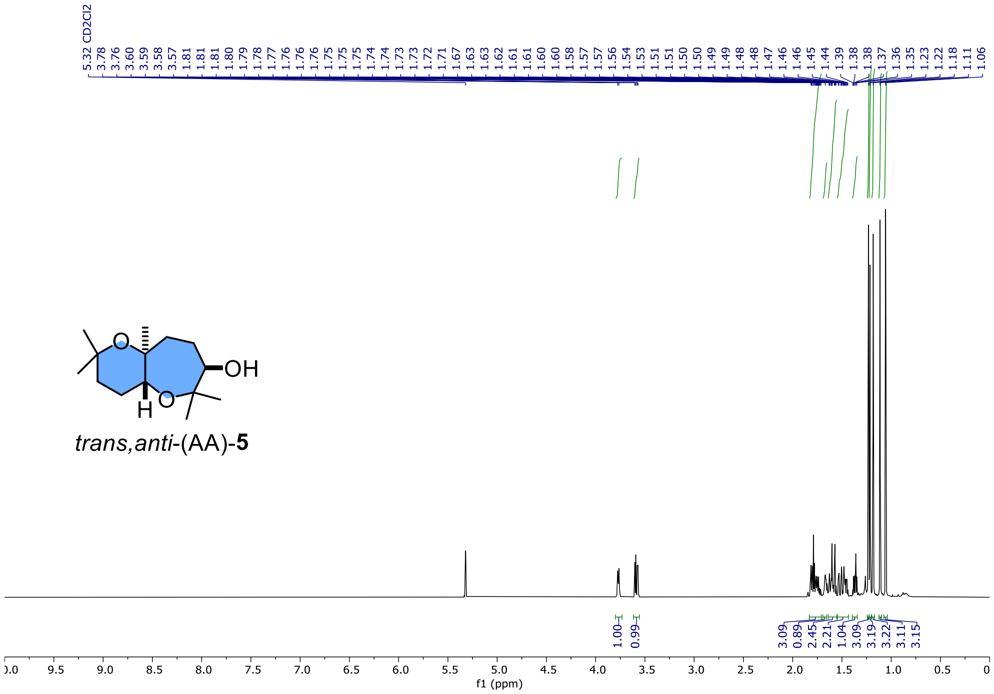

Figure S56. 500 MHz ^{1}H NMR spectrum of *trans,anti*-(AA)-**5** in CD_2Cl_2.

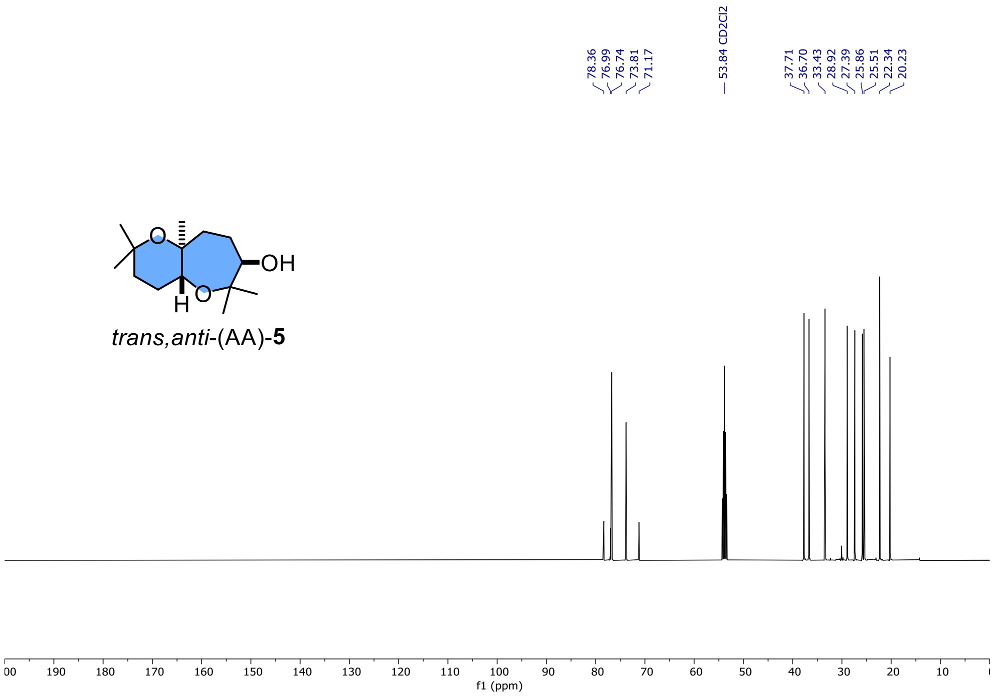

Figure S57. 125 MHz ^{13}C NMR spectrum of *trans,anti*-(AA)-**5** in CD_2Cl_2.

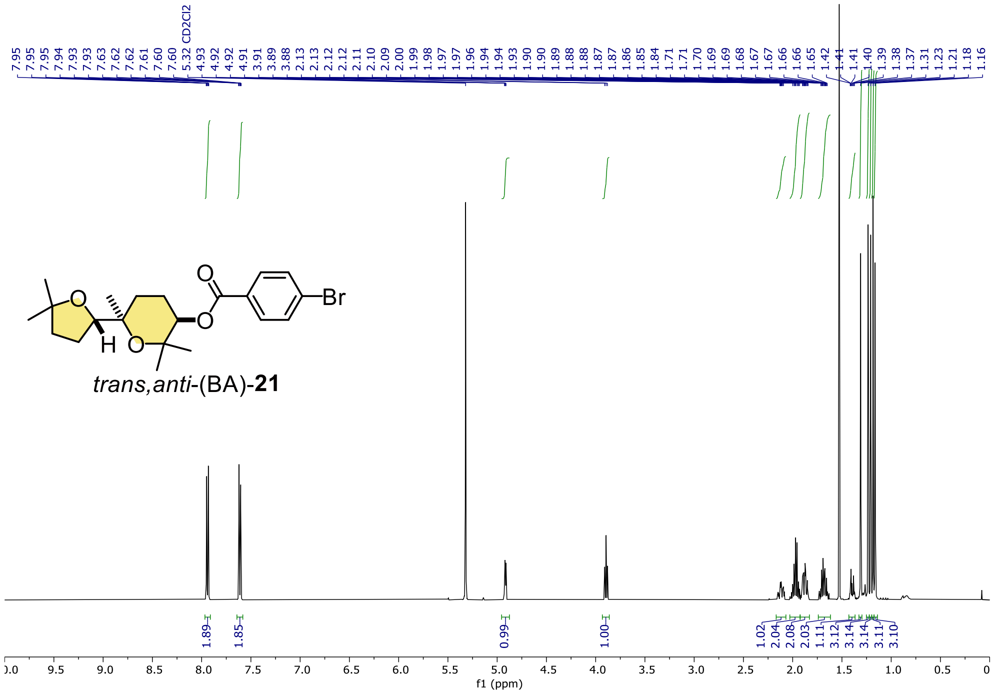

Figure S58. 500 MHz ^{1}H NMR spectrum of *trans*,*anti*-(BA)-**21** in CD_2Cl_2.

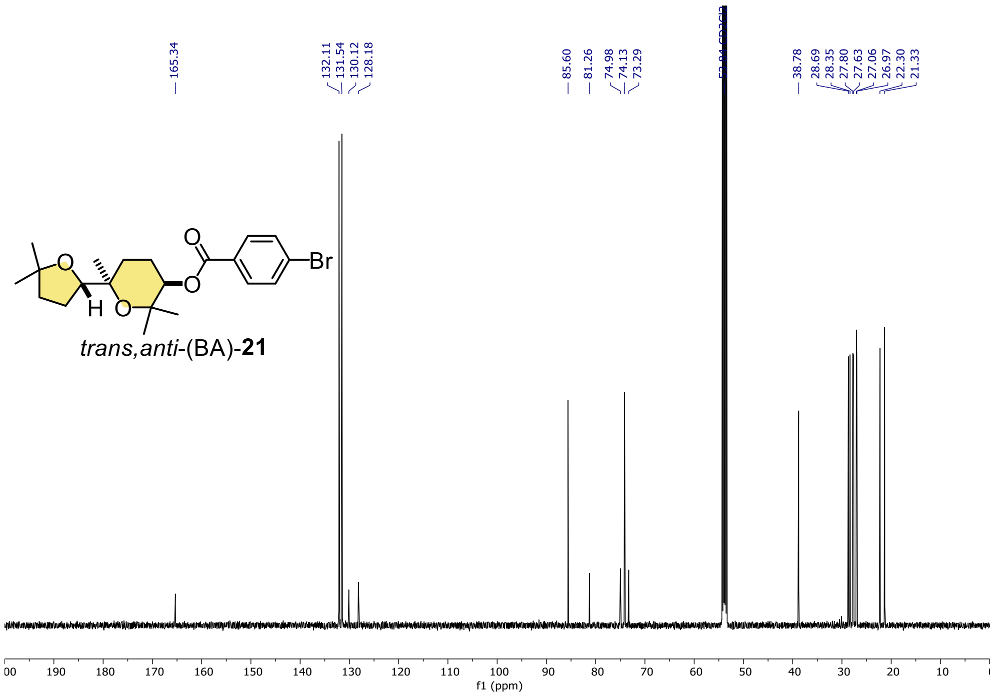

Figure S59. 125 MHz ^{13}C NMR spectrum of *trans*,*anti*-(BA)-**21** in CD_2Cl_2.

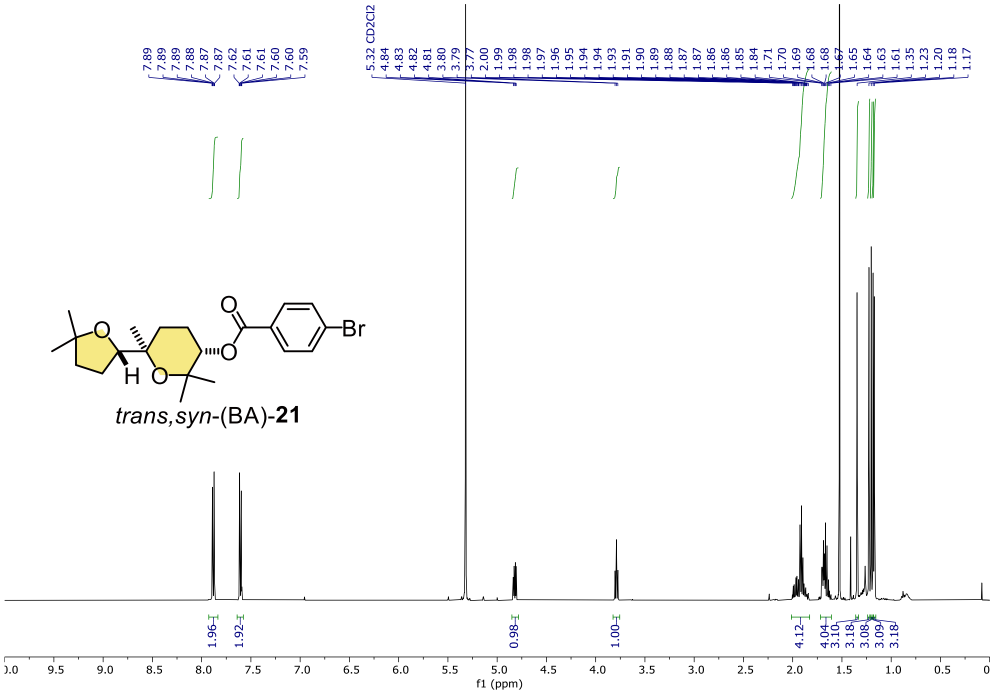

Figure S60. 500 MHz ^{1}H NMR spectrum of *trans*,*syn*-(BA)-**21** in CD_2Cl_2.

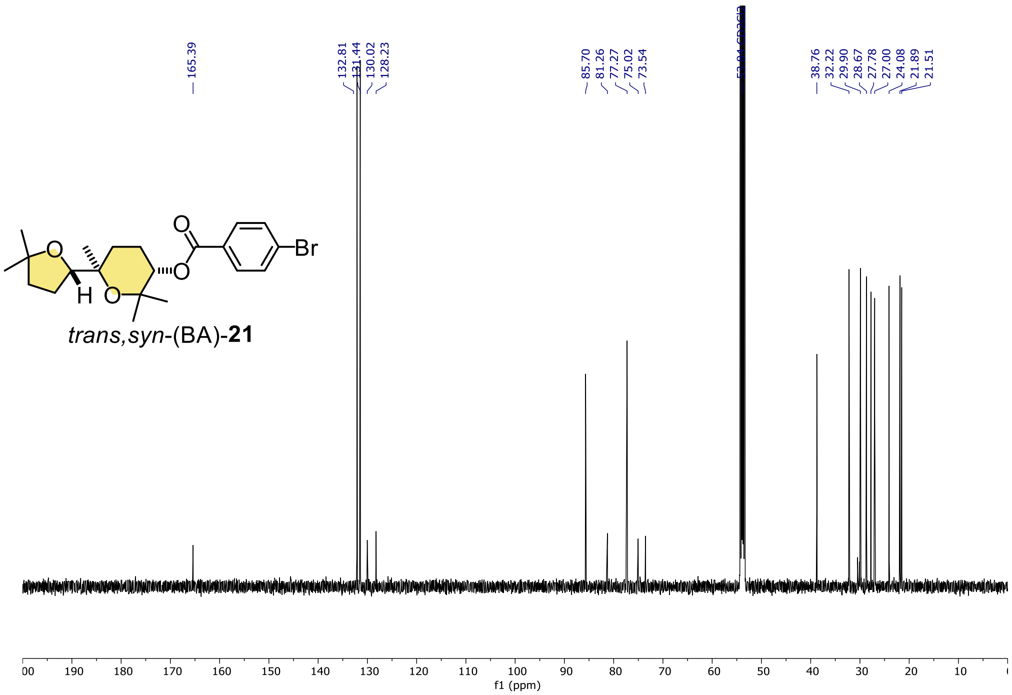

Figure S61. 125 MHz ^{13}C NMR spectrum of *trans*,*syn*-(BA)-**21** in CD_2Cl_2.

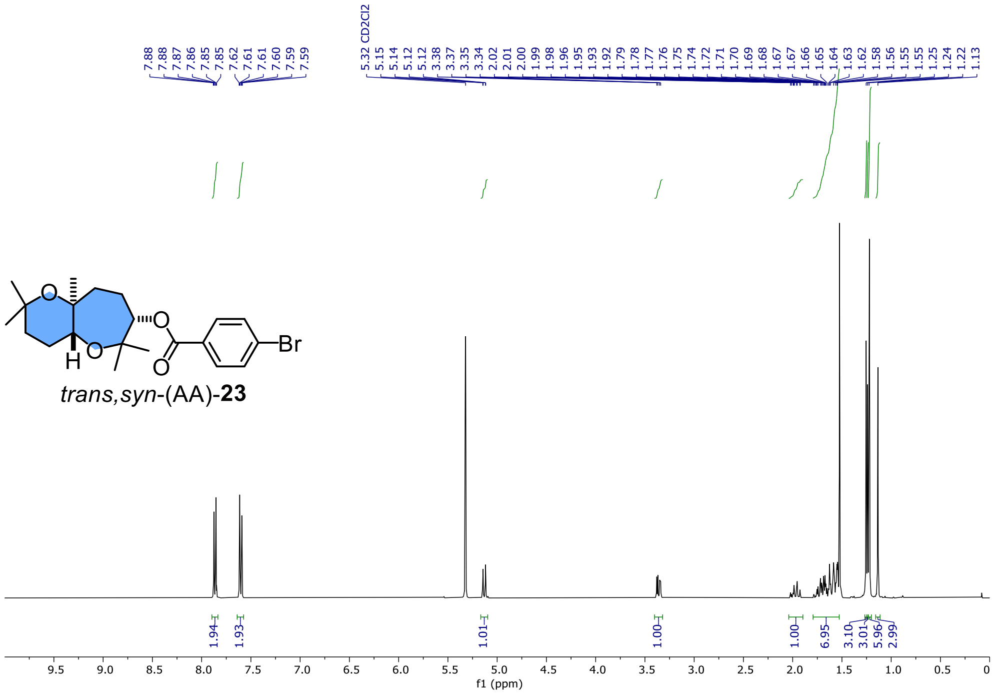

Figure S62. 400 MHz ^{1}H NMR spectrum of *trans,syn*-(AA)-**23** in CD_2Cl_2.

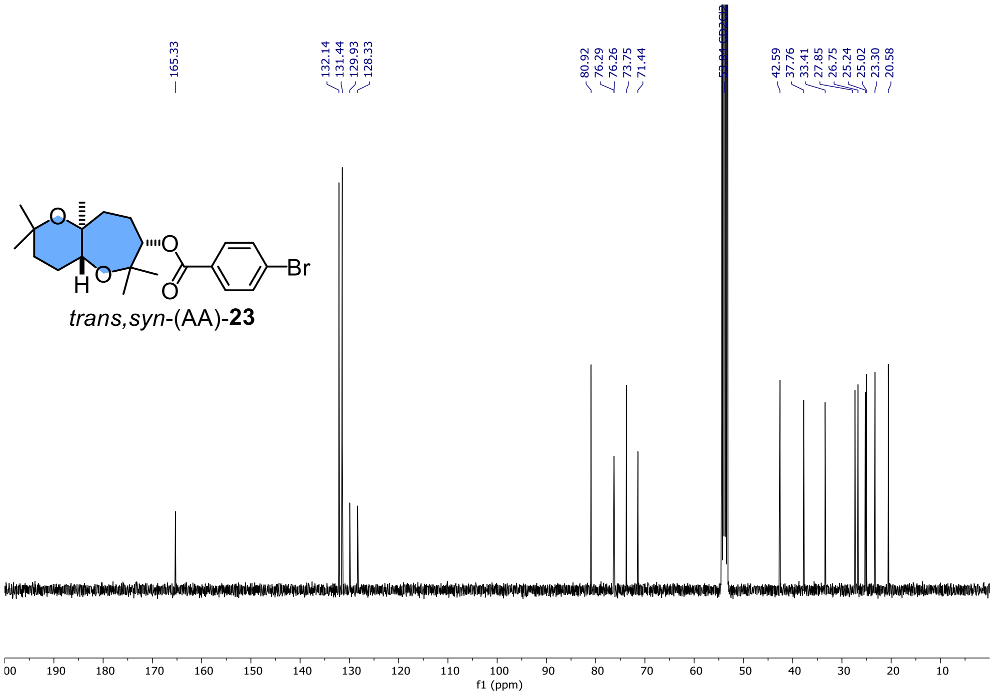

Figure S63. 100 MHz ^{13}C NMR spectrum of *trans,syn*-(AA)-**23** in CD_2Cl_2.

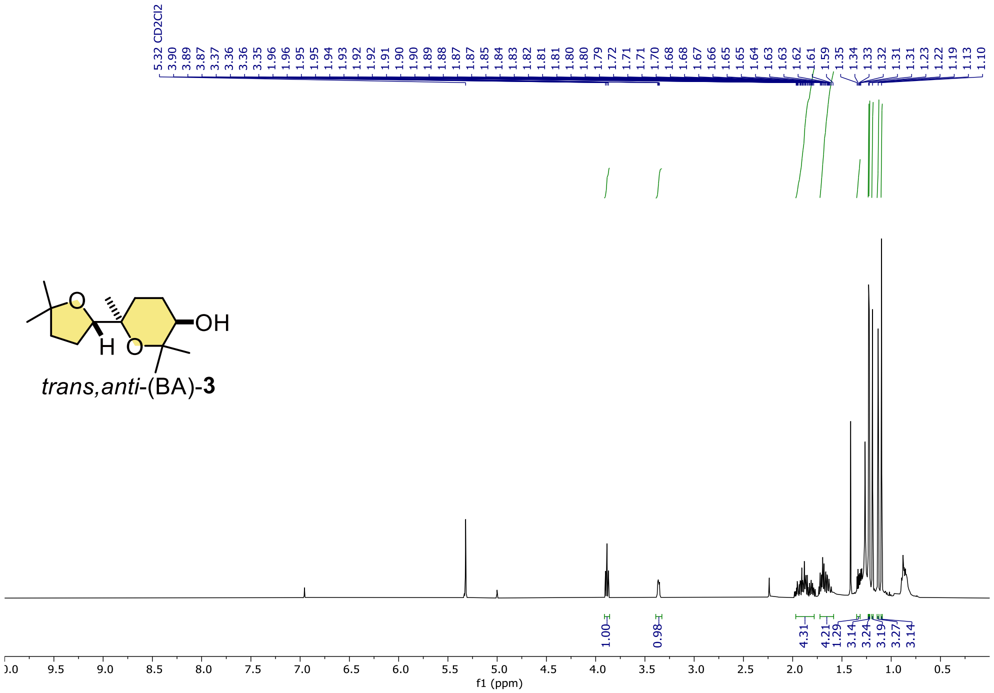

Figure S64. 500 MHz ^{1}H NMR spectrum of *trans,anti*-(BA)-**3** in CD_2Cl_2.

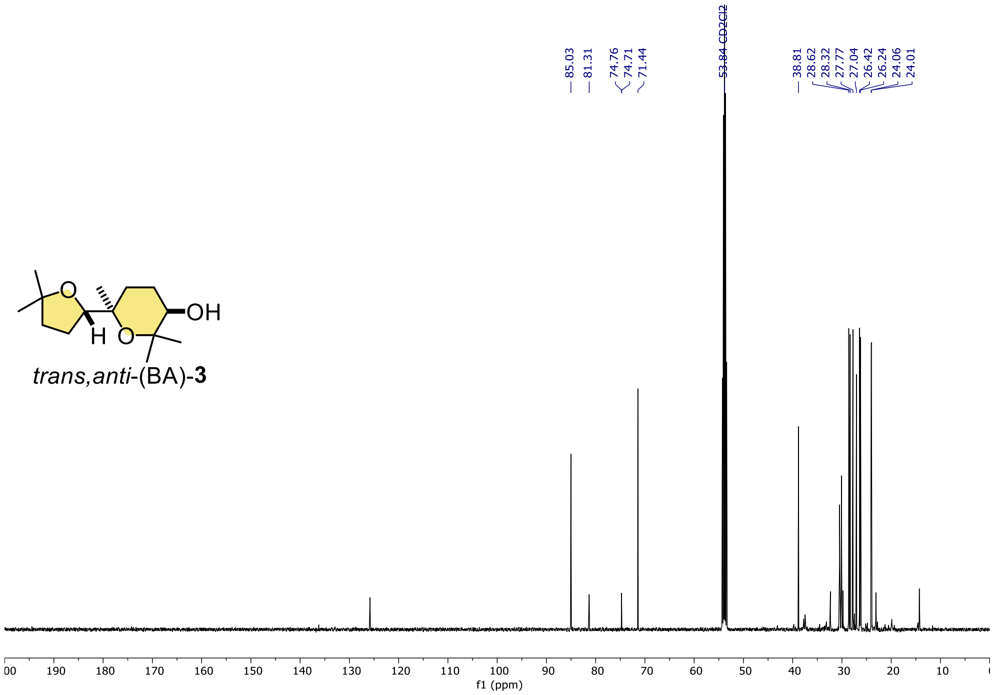

Figure S65. 125 MHz ^{13}C NMR spectrum of *trans,anti*-(BA)-**3** in CD_2Cl_2.

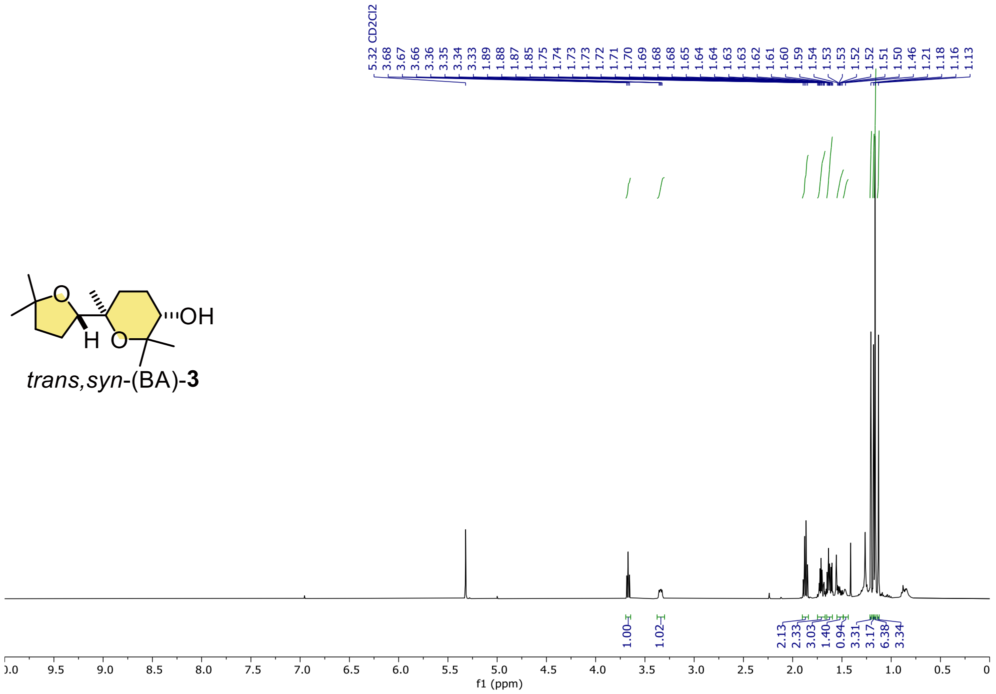

Figure S66. 500 MHz ^{1}H NMR spectrum of *trans*,*syn*-(BA)-**3** in CD_2Cl_2.

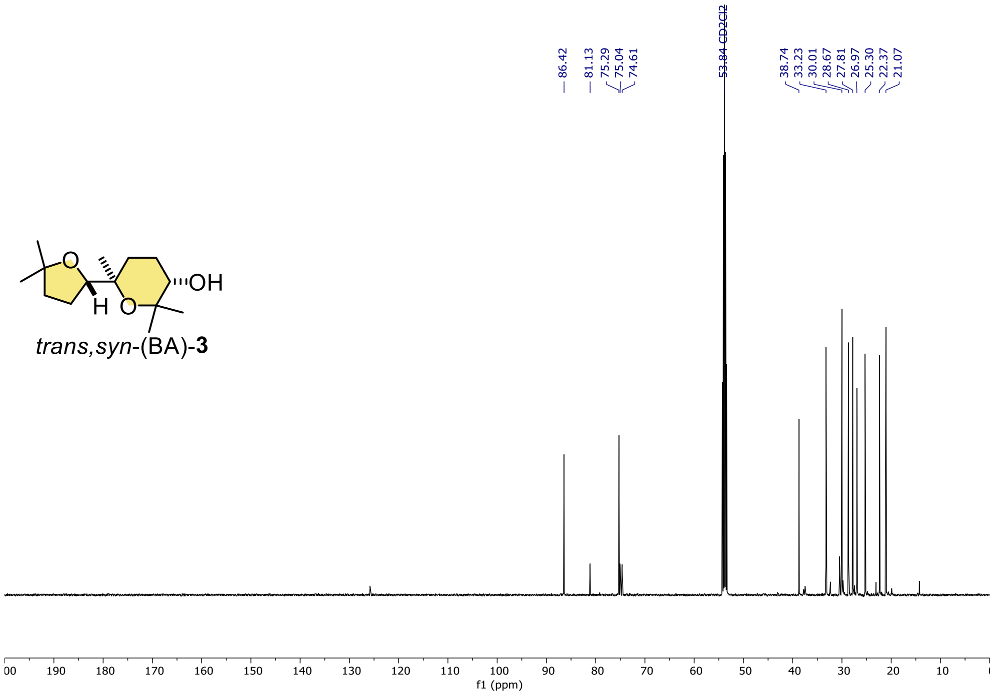

Figure S67. 125 MHz ^{13}C NMR spectrum of *trans*,*syn*-(BA)-**3** in CD_2Cl_2.

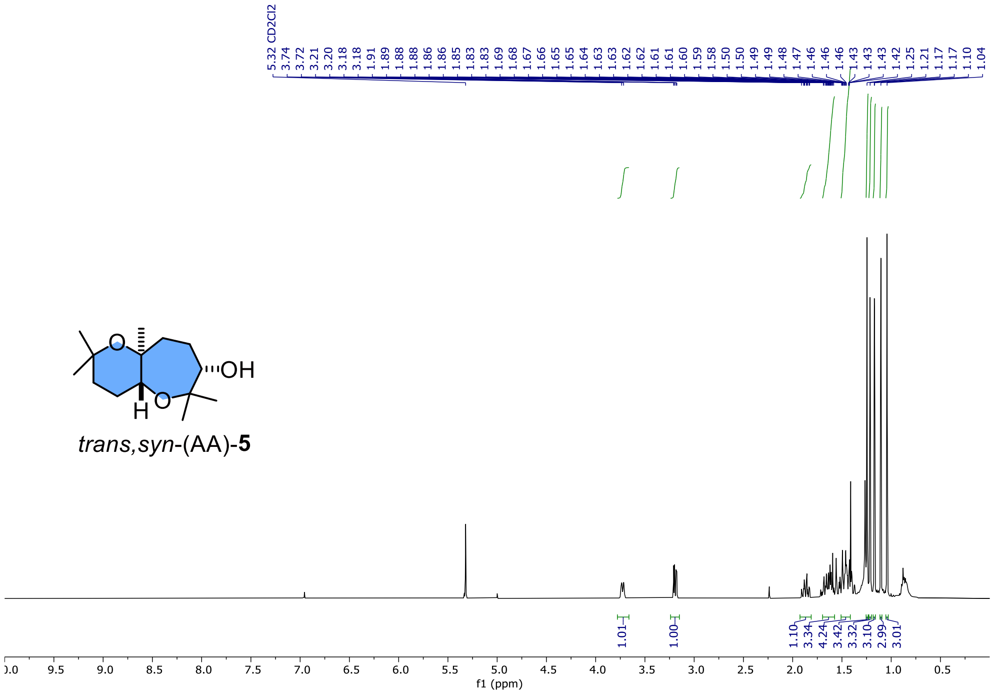

Figure S68. 500 MHz ^{1}H NMR spectrum of *trans,syn*-(AA)-**5** in CD_2Cl_2.

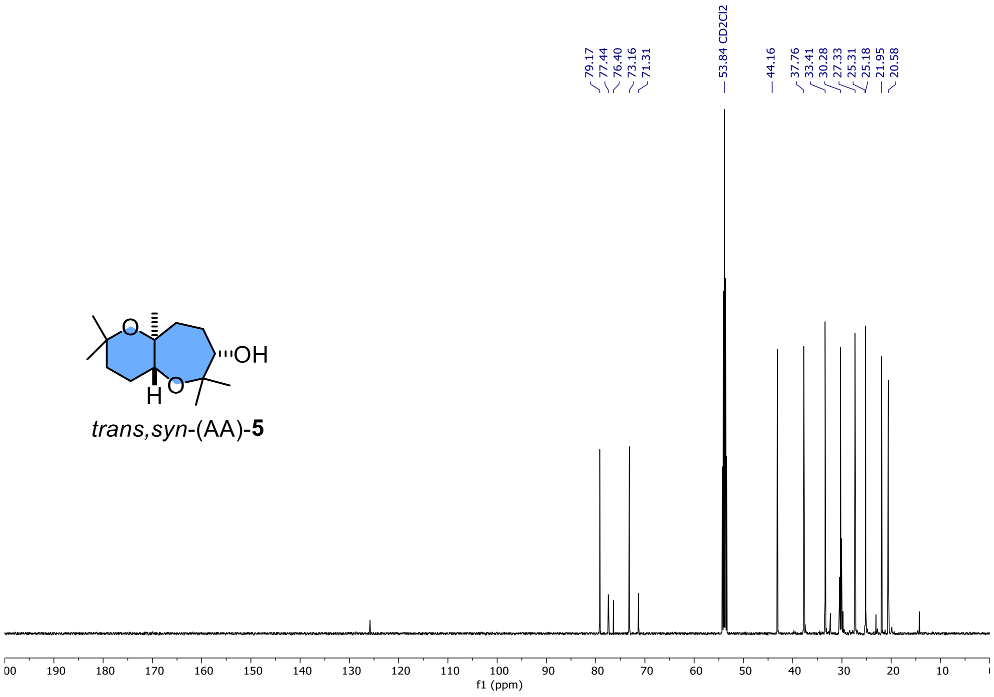

Figure S69. 125 MHz ^{13}C NMR spectrum of *trans,syn*-(AA)-**5** in CD_2Cl_2.

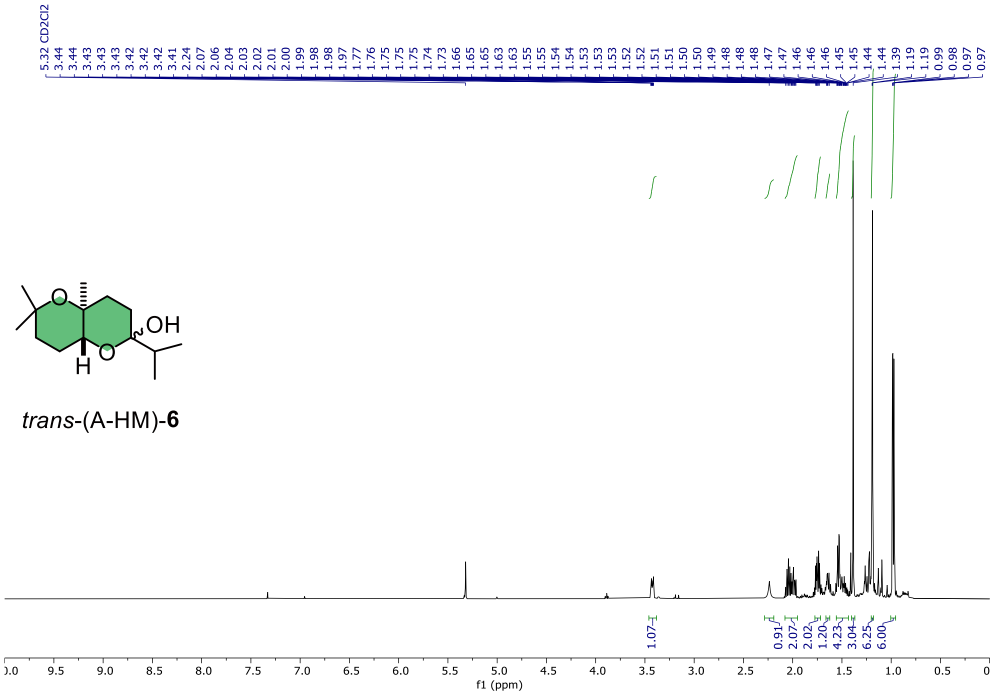

Figure S70. 500 MHz ^{1}H NMR spectrum of *trans*-(A-HM)-**6** in CD_2Cl_2.

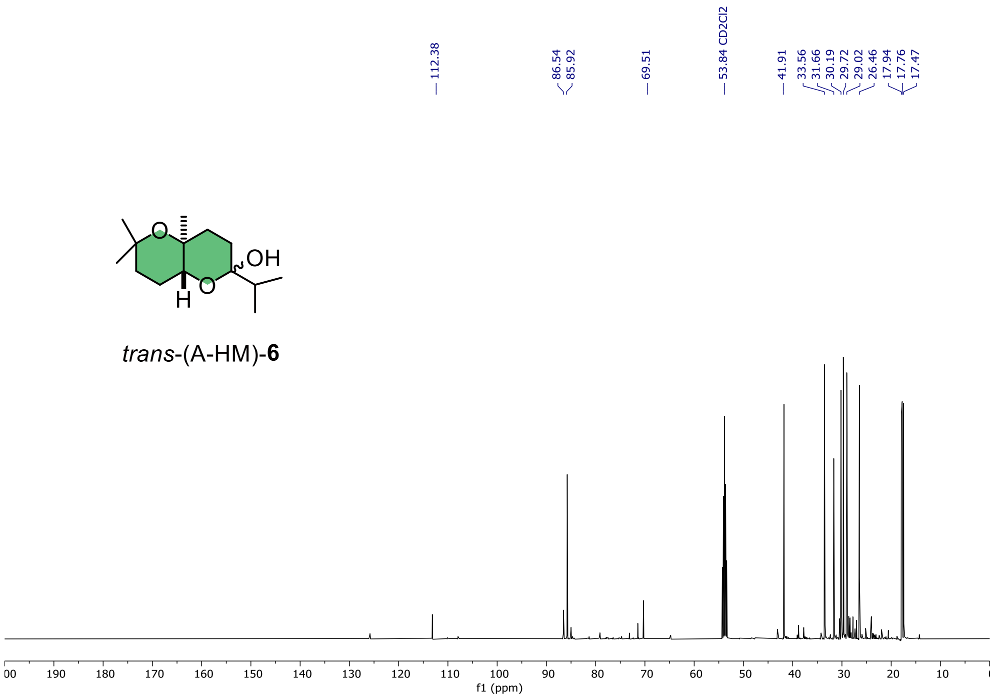

Figure S71. 125 MHz ^{13}C NMR spectrum of *trans*-(A-HM)-**6** in CD_2Cl_2.

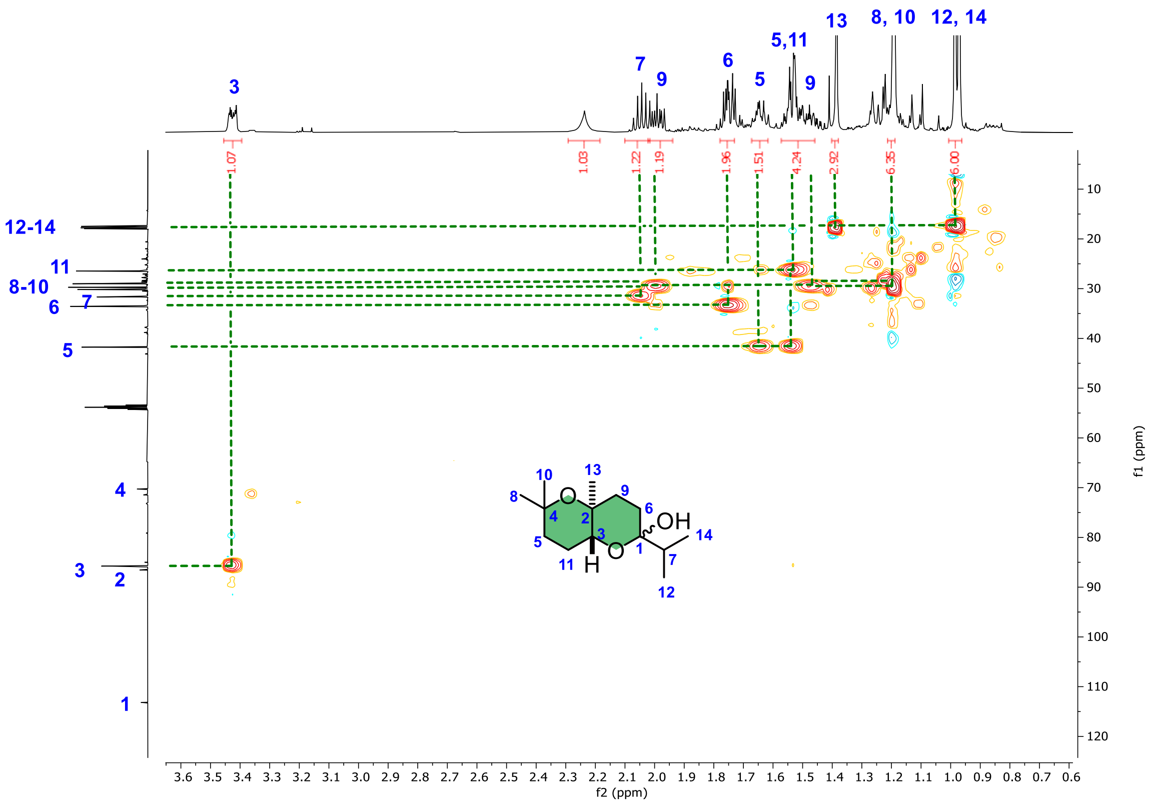

Figure S72. HSQC spectrum of *trans*-(A-HM)-**6** in CD_2Cl_2.

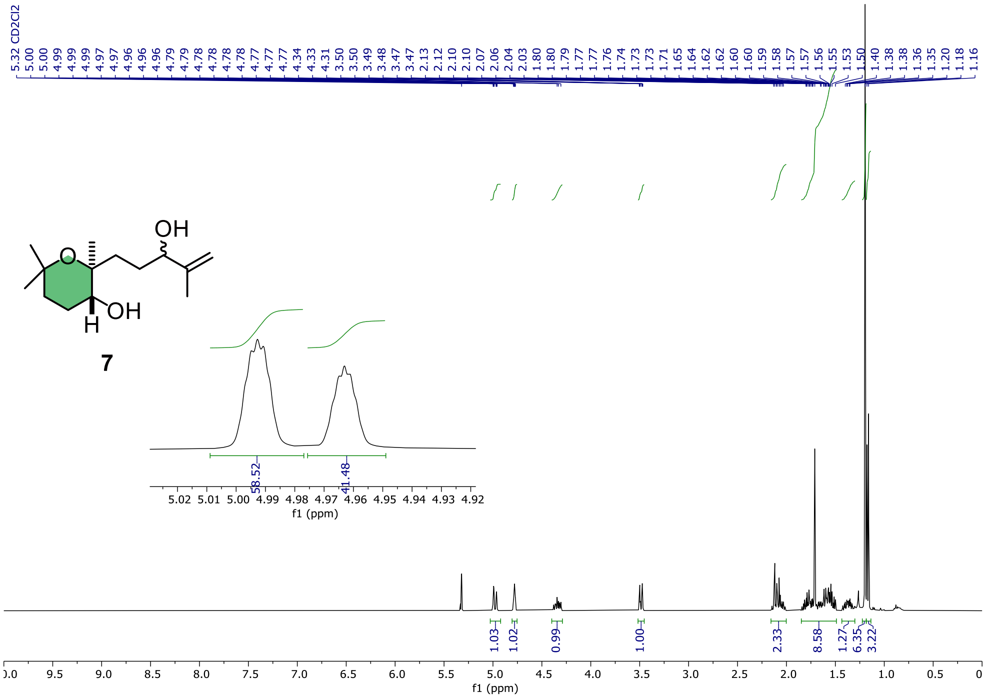

Figure S73. 400 MHz ^{1}H NMR spectrum of **7** in CD_2Cl_2.

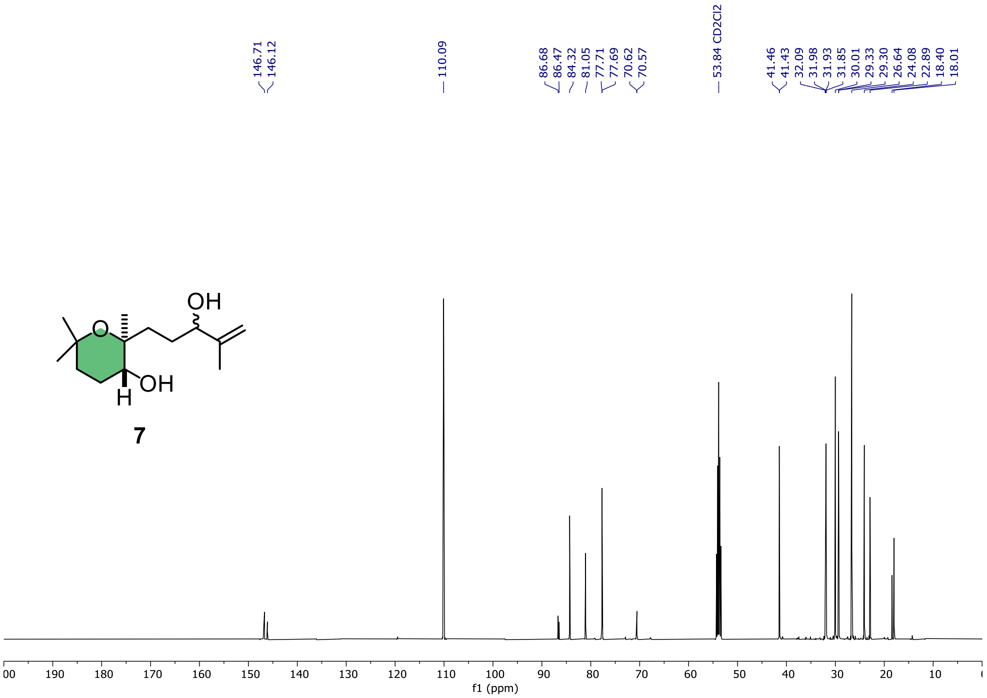

Figure S74. 100 MHz ^{13}C NMR spectrum of **7** in CD_2Cl_2.

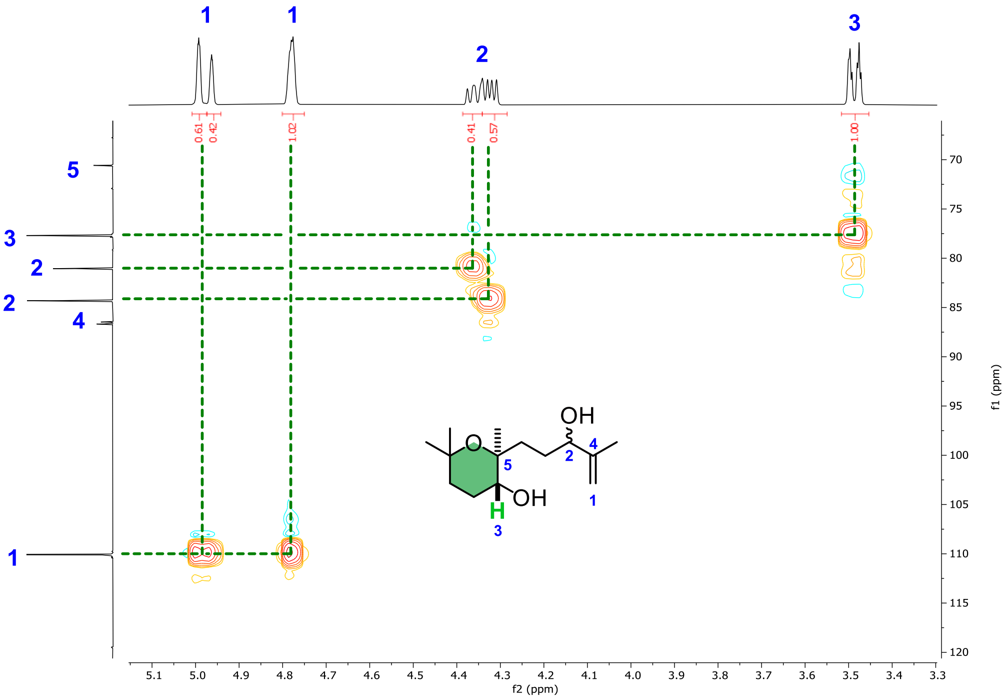

Figure S75. Zoomed HSQC spectrum of **7** in CD_2Cl_2.

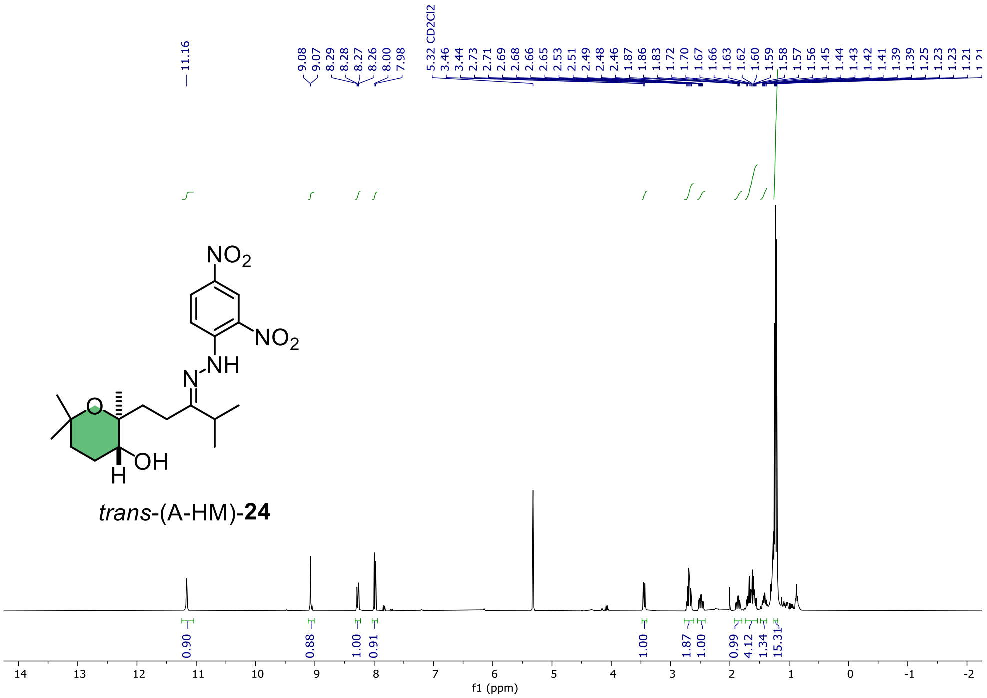

Figure S76. 400 MHz ^{1}H NMR spectrum of *trans*-(A-HM)-**24** in CD_2Cl_2.

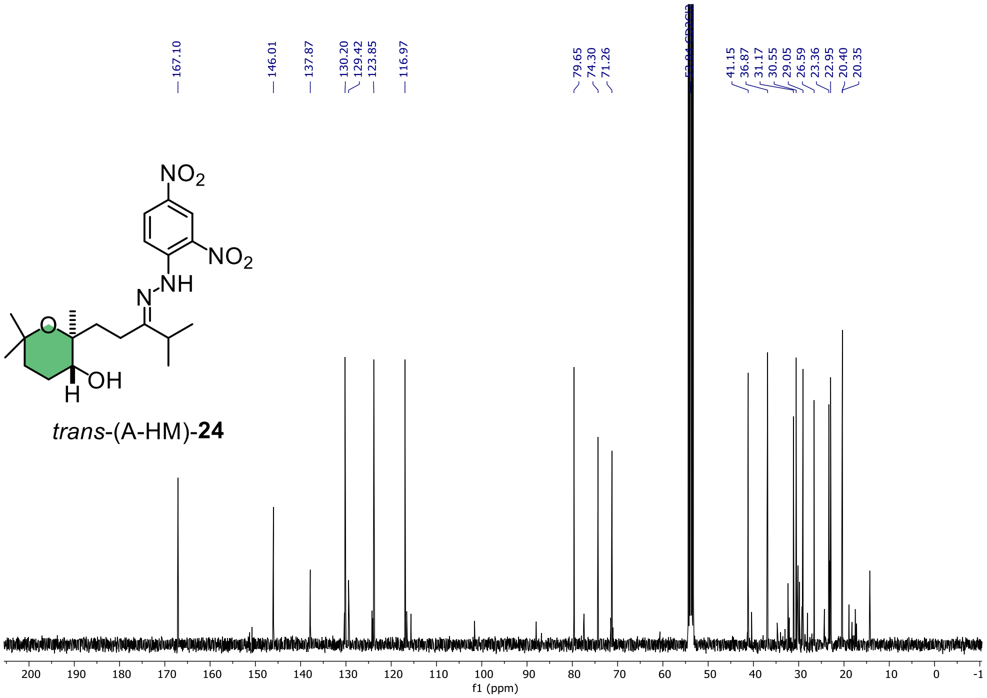

Figure S77. 100 MHz ^{13}C NMR spectrum of *trans*-(A-HM)-**24** in CD_2Cl_2.

7. X-ray crystallography

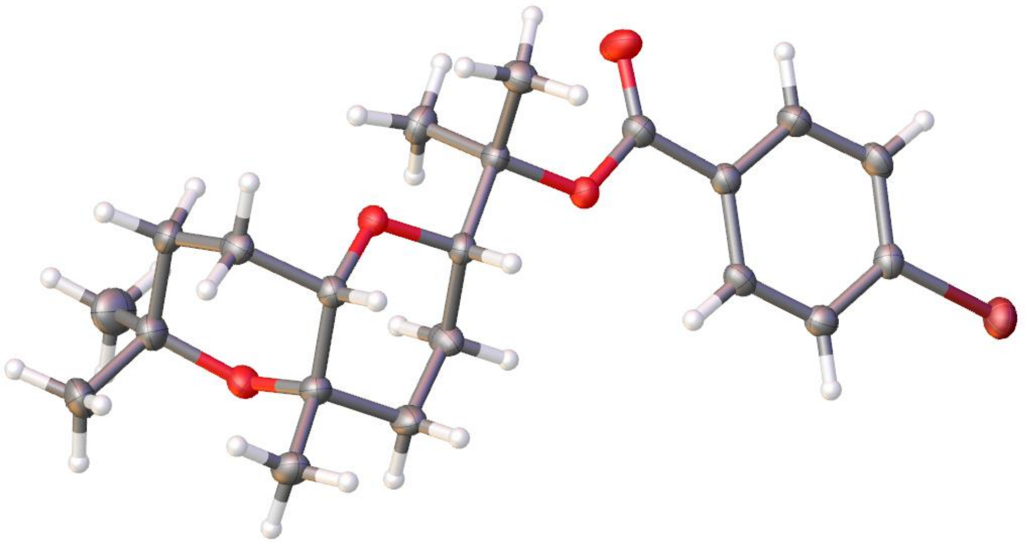

Figure S78. *cis*,*anti*-(AB)-**22**: View of the molecule (displacement ellipsoids drawn at 50 percent probability level).

Table S3 Crystal data and structure refinement for *cis*,*anti*-(AB)-**22**.

CCDC number	2176606	
Empirical formula	$C_{21}H_{29}BrO_4$	
Formula weight	425.35	
Temperature	119.99(10) K	
Wavelength	1.54184 Å	
Crystal system	Monoclinic	
Space group	P 1 21/c 1	
Unit cell dimensions	a = 14.0432(5) Å	α = 90°
	b = 7.0309(3) Å	β = 100.447(4)°

	c = 21.1186(8) Å γ = 90°
Volume	2050.61(14) $Å^3$
Z	4
Density (calculated)	1.378 Mg/m^3
Absorption coefficient	2.911 mm^{-1}
F(000)	888
Crystal size	0.451 x 0.035 x 0.026 mm^3
Theta range for data collection	3.200 to 74.569°.
Index ranges	-17<=h<=16, -8<=k<=7, -25<=l<=26
Reflections collected	30520
Independent reflections	4126 [R(int) = 0.0285]
Completeness to theta = 67.684°	100.0 %
Absorption correction	Gaussian
Max. and min. transmission	1.000 and 0.435
Refinement method	Full-matrix least-squares on F^2
Data / restraints / parameters	4126 / 0 / 240
Goodness-of-fit on F^2	1.072
Final R indices [I>2sigma(I)]	R1 = 0.0264, wR2 = 0.0677
R indices (all data)	R1 = 0.0292, wR2 = 0.0691
Extinction coefficient	n/a
Largest diff. peak and hole	0.298 and -0.480 e.$Å^{-3}$

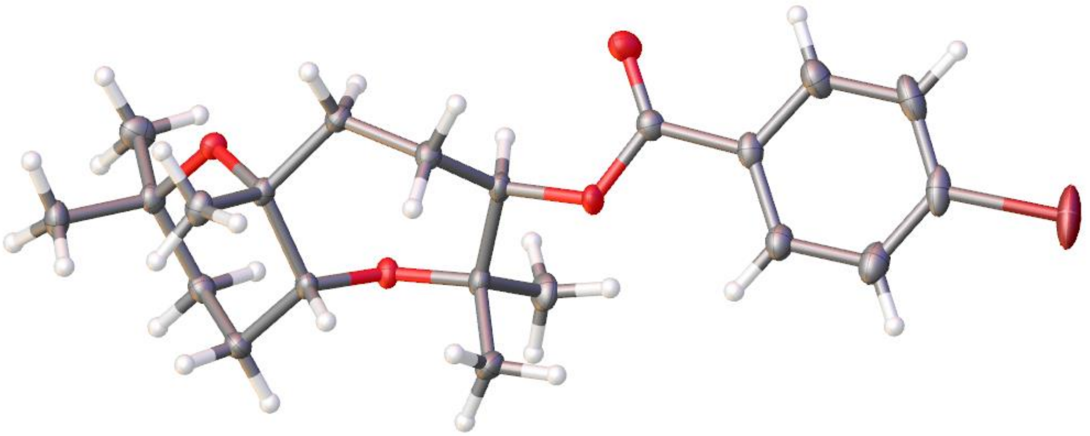

Figure S79. *cis,syn*-(AA)-**23**: View of the molecule (displacement ellipsoids drawn at 50 percent probability level).

Table S4 Crystal data and structure refinement for *cis,syn*-(AA)-**23**.

CCDC number	2176601	
Empirical formula	$C_{21}H_{29}BrO_4$	
Formula weight	425.35	
Temperature	129(14) K	
Wavelength	1.54184 Å	
Crystal system	Triclinic	
Space group	P-1	
Unit cell dimensions	a = 6.36017(18) Å	α = 91.523(4)°
	b = 7.8529(2) Å	β = 91.016(4)°
	c = 20.1551(10) Å	γ = 93.880(2)°

Volume	1003.82(6) Å^3
Z	2
Density (calculated)	1.407 Mg/m^3
Absorption coefficient	2.973 mm^{-1}
F(000)	444
Crystal size	0.247 x 0.171 x 0.045 mm^3
Theta range for data collection	2.193 to 74.639°.
Index ranges	-7<=h<=4, -9<=k<=9, -24<=l<=24
Reflections collected	20722
Independent reflections	3968 [R(int) = 0.0412]
Completeness to theta = 67.684°	99.8 %
Absorption correction	Gaussian
Max. and min. transmission	1.000 and 0.415
Refinement method	Full-matrix least-squares on F^2
Data / restraints / parameters	3968 / 0 / 240
Goodness-of-fit on F^2	1.033
Final R indices [I>2sigma(I)]	R1 = 0.0344, wR2 = 0.0768
R indices (all data)	R1 = 0.0375, wR2 = 0.0788
Extinction coefficient	n/a
Largest diff. peak and hole	0.994 and -1.163 e.Å^{-3}

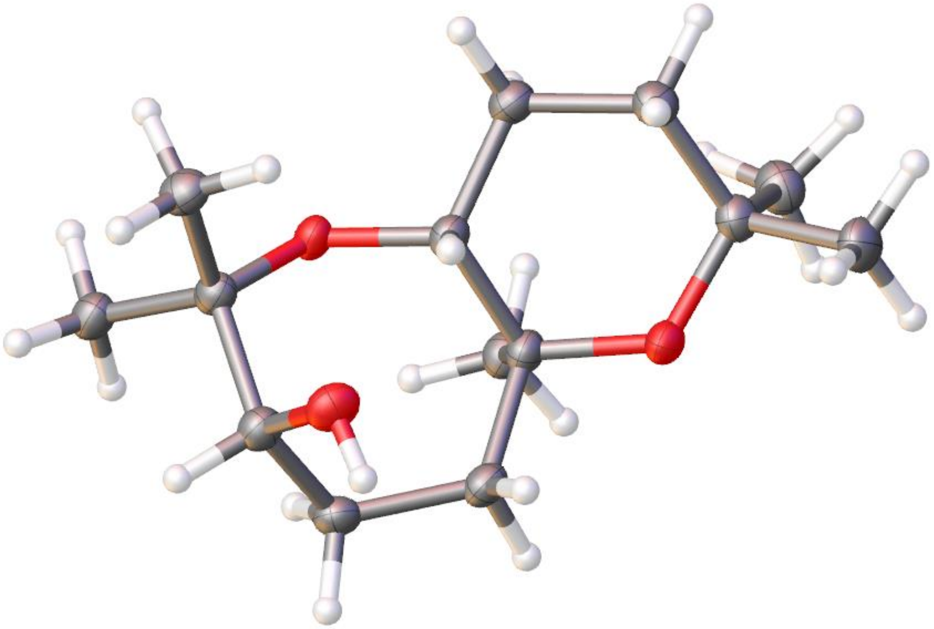

Figure S80. *trans,anti*-(AA)-**5**. View of the molecule (displacement ellipsoids drawn at 50 percent probability level)

Table S5 Crystal data and structure refinement for *trans,anti*-(AA)-**5**.

CCDC number	2176603	
Empirical formula	$C_{14}H_{26}O_3$	
Formula weight	242.35	
Temperature	119.99(10) K	
Wavelength	1.54184 Å	
Crystal system	Monoclinic	
Space group	P 1 21/n 1	
Unit cell dimensions	a = 5.8614(2) Å	α = 90°
	b = 15.5412(7) Å	β = 90.764(4)°

	c = 14.8746(7) Å	γ = 90°
Volume	1354.85(10) $Å^3$	
Z	4	
Density (calculated)	1.188 Mg/m^3	
Absorption coefficient	0.646 mm^{-1}	
F(000)	536	
Crystal size	0.083 x 0.041 x 0.023 mm^3	
Theta range for data collection	4.114 to 74.876°.	
Index ranges	-3<=h<=7, -19<=k<=19, -18<=l<=18	
Reflections collected	18499	
Independent reflections	2728 [R(int) = 0.0827]	
Completeness to theta = 67.684°	99.8 %	
Absorption correction	Semi-empirical from equivalents	
Max. and min. transmission	1.00000 and 0.49864	
Refinement method	Full-matrix least-squares on F^2	
Data / restraints / parameters	2728 / 1 / 162	
Goodness-of-fit on F^2	1.088	
Final R indices [I>2sigma(I)]	R1 = 0.0735, wR2 = 0.1494	
R indices (all data)	R1 = 0.0840, wR2 = 0.1561	
Extinction coefficient	n/a	
Largest diff. peak and hole	0.254 and -0.257 e.$Å^{-3}$	

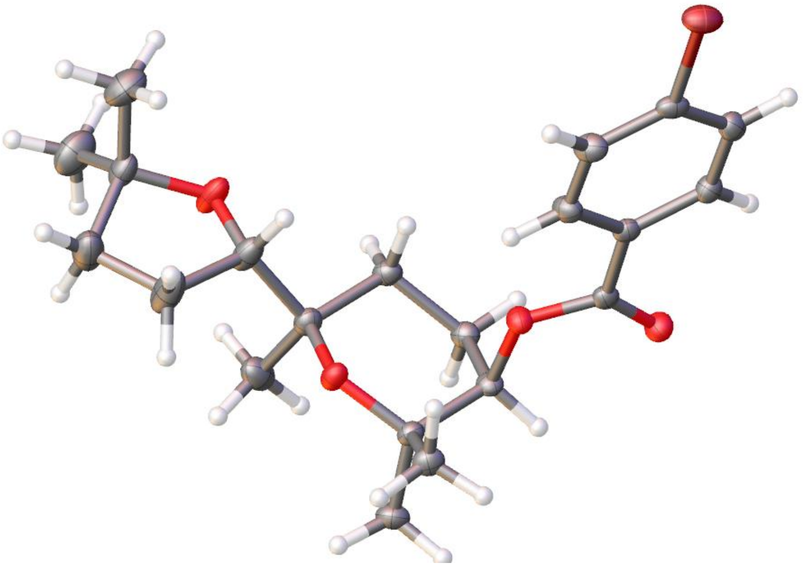

Figure S81. *trans*,*anti*-(BA)-**21**. View of the molecule (displacement ellipsoids drawn at 50 percent probability level).

Table S6 Crystal data and structure refinement for *trans*,*anti*-(BA)-**21**.

CCDC number	2176605	
Empirical formula	$C_{21}H_{29}BrO_4$	
Formula weight	425.35	
Temperature	120.01(10) K	
Wavelength	1.54184 Å	
Crystal system	Triclinic	
Space group	P-1	
Unit cell dimensions	a = 7.44418(10) Å	α = 69.1422(14)°
	b = 11.53654(18) Å	β = 81.6780(12)°

	c = 12.84945(19) Å γ = 84.3027(12)°
Volume	1018.98(3) $Å^3$
Z	2
Density (calculated)	1.386 Mg/m^3
Absorption coefficient	2.929 mm^{-1}
F(000)	444
Crystal size	0.33 x 0.26 x 0.09 mm^3
Theta range for data collection	3.707 to 74.780°.
Index ranges	-9<=h<=7, -14<=k<=14, -15<=l<=15
Reflections collected	22323
Independent reflections	4059 [R(int) = 0.0242]
Completeness to theta = 67.684°	99.9 %
Absorption correction	Analytical
Max. and min. transmission	0.786 and 0.465
Refinement method	Full-matrix least-squares on F^2
Data / restraints / parameters	4059 / 0 / 240
Goodness-of-fit on F^2	1.053
Final R indices [I>2sigma(I)]	R1 = 0.0247, wR2 = 0.0620
R indices (all data)	R1 = 0.0251, wR2 = 0.0623
Extinction coefficient	n/a
Largest diff. peak and hole	0.499 and -0.416 e.$Å^{-3}$

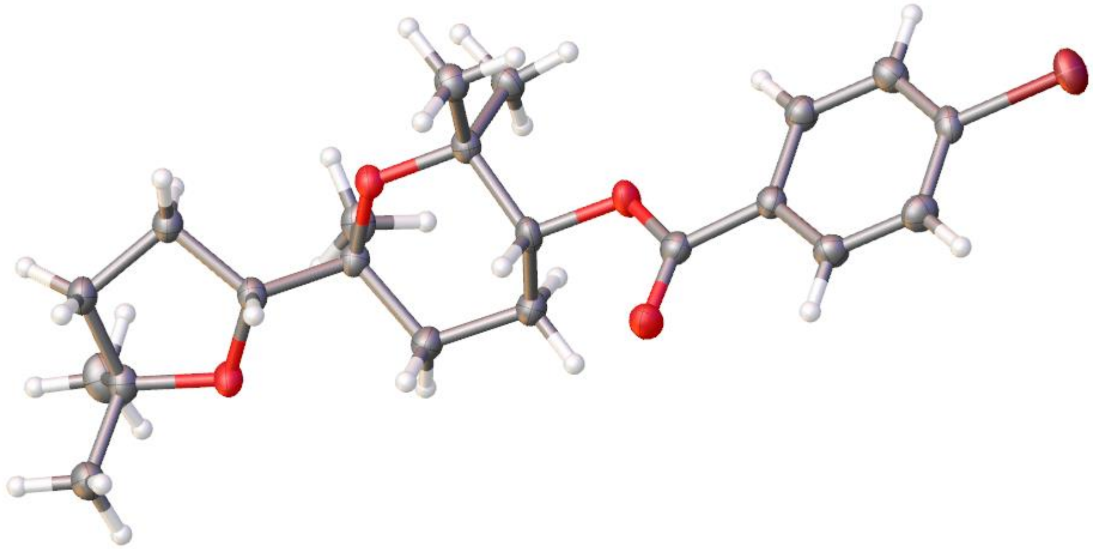

Figure S82. *trans*,*syn*-(BA)-**21**. View of the molecule (displacement ellipsoids drawn at 50 percent probability level).

Table S7 Crystal data and structure refinement for *trans*,*syn*-(BA)-**21**.

CCDC number	2176607	
Empirical formula	$C_{21}H_{29}BrO_4$	
Formula weight	425.35	
Temperature	119.99(10) K	
Wavelength	1.54184 Å	
Crystal system	Triclinic	
Space group	P-1	
Unit cell dimensions	a = 6.41587(11) Å	α = 94.139(2)°
	b = 12.0405(3) Å	β = 91.6023(17)°

	c = 13.3039(3) Å	γ = 101.2879(17)°
Volume	1004.29(4) $Å^3$	
Z	2	
Density (calculated)	1.407 Mg/m^3	
Absorption coefficient	2.972 mm^{-1}	
F(000)	444	
Crystal size	0.31 x 0.04 x 0.03 mm^3	
Theta range for data collection	3.334 to 74.508°.	
Index ranges	-4<=h<=7, -14<=k<=14, -16<=l<=16	
Reflections collected	14479	
Independent reflections	3964 [R(int) = 0.0303]	
Completeness to theta = 67.684°	99.9 %	
Absorption correction	Analytical	
Max. and min. transmission	0.928 and 0.638	
Refinement method	Full-matrix least-squares on F^2	
Data / restraints / parameters	3964 / 0 / 240	
Goodness-of-fit on F^2	1.060	
Final R indices [I>2sigma(I)]	R1 = 0.0361, wR2 = 0.0960	
R indices (all data)	R1 = 0.0412, wR2 = 0.0986	
Extinction coefficient	n/a	
Largest diff. peak and hole	0.447 and -0.839 e.$Å^{-3}$	

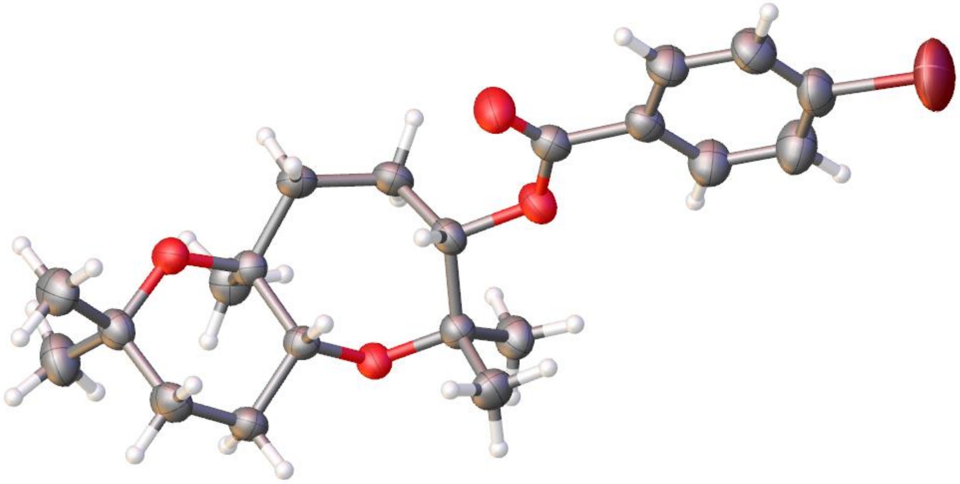

Figure S83. *trans*,*syn*-(AA)-**23**. View of the molecule (displacement ellipsoids drawn at 50 percent probability level).

Table S8 Crystal data and structure refinement for *trans*,*syn*-(AA)-**23**.

CCDC number	2176602	
Empirical formula	$C_{21}H_{29}BrO_4$	
Formula weight	425.35	
Temperature	296.0(3) K	
Wavelength	1.54184 Å	
Crystal system	Orthorhombic	
Space group	Pbca	
Unit cell dimensions	a = 10.6941(3) Å	α = 90°
	b = 8.2180(2) Å	β = 90°
	c = 46.3766(14) Å	γ = 90°

Volume	4075.8(2) Å^3
Z	8
Density (calculated)	1.386 Mg/m^3
Absorption coefficient	2.929 mm^{-1}
F(000)	1776
Crystal size	0.335 x 0.257 x 0.064 mm^3
Theta range for data collection	3.813 to 73.231°.
Index ranges	-12<=h<=10, -9<=k<=9, -56<=l<=56
Reflections collected	69471
Independent reflections	3965 [R(int) = 0.0305]
Completeness to theta = 67.684°	99.9 %
Absorption correction	Semi-empirical from equivalents
Max. and min. transmission	1.00000 and 0.60349
Refinement method	Full-matrix least-squares on F^2
Data / restraints / parameters	3965 / 0 / 240
Goodness-of-fit on F^2	1.051
Final R indices [I>2sigma(I)]	R1 = 0.0430, wR2 = 0.1083
R indices (all data)	R1 = 0.0444, wR2 = 0.1094
Extinction coefficient	n/a
Largest diff. peak and hole	0.782 and -0.872 e.Å^{-3}

8. Supplementary references

S1 M. Paraja, X. Hao and S. Matile, *Angew. Chem. Int. Ed.*, 2020, **59**, 15093–15097.

S2 A. Gini, M. Paraja, B. Galmés, C. Besnard, A. I. Poblador-Bahamonde, N. Sakai, A. Frontera and S. Matile, *Chem. Sci.*, 2020, **11**, 7086–7091.

S3 X. Zhang, X. Hao, L. Le, A.-T. Pham, J. López-Andarias, A. Frontera, N. Sakai and S. Matile, *J. Am. Chem. Soc.*, 2018, **140**, 17867–17871.

S4 J. M. Köster and K. Tiefenbacher, *ChemCatChem*, 2018, **10**, 2941–2944.

S5 S. Sakane, J. Fujiwara, K. Maruoka and H. Yamamoto, *Tetrahedron*, 1986, **42**, 2193–2201.

S6 P. Ondet, L. Lempenauer, E. Duñach and G. Lemière, *Org. Chem. Front.*, 2016, **3**, 999–1003.

S7 T. B. Towne and F. E. McDonald, *J. Am. Chem. Soc.*, 1997, **119**, 6022–6028.

S8 F.-X. Li, S.-J. Ren, P.-F. Li, P. Yang and J. Qu, *Angew. Chem. Int. Ed.*, 2020, **59**, 18473–18478.

S9 M.-P. Fernando and P.-B. Joaquin, *J. Chem. Educ.*, 1987, **64**, 925–927.

S10 L. M. Schwartz, *J. Chem. Educ.*, 1989, **66**, 677.
